# Discovery of Protease-Activated Receptor 4 (PAR4)-Tethered Ligand Antagonists Using Ultralarge Virtual Screening

**DOI:** 10.1021/acsptsci.3c00378

**Published:** 2024-03-21

**Authors:** Shannon T. Smith, Jackson B. Cassada, Lukas Von Bredow, Kevin Erreger, Emma M. Webb, Trevor A. Trombley, Jacob J. Kalbfleisch, Brian J. Bender, Irene Zagol-Ikapitte, Valerie M. Kramlinger, Jacob L. Bouchard, Sidnee G. Mitchell, Maik Tretbar, Brian K. Shoichet, Craig W. Lindsley, Jens Meiler, Heidi E. Hamm

**Affiliations:** † Department of Pharmacology, Vanderbilt University, Nashville, Tennessee 37232, United States; ‡ Department of Chemistry, Vanderbilt University, Nashville, Tennessee 37232, United States; § Warren Center for Neuroscience Drug Discovery, Nashville, Tennessee 37067, United States; ∥ Department of Pharmaceutical Chemistry, University of California San Francisco, San Francisco, California 94158, United States; ⊥ Institute for Drug Discovery, Leipzig University Medical School, Leipzig 04109, Germany

**Keywords:** G-protein-coupled receptors, protease-activated receptor 4, antithrombotic therapeutics, virtual high-throughput screening, structure-based drug discovery, molecular docking

## Abstract

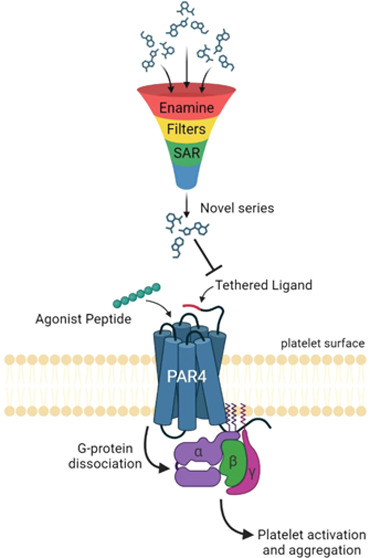

Here, we demonstrate a structure-based small molecule virtual screening and lead optimization pipeline using a homology model of a difficult-to-drug G-protein-coupled receptor (GPCR) target. Protease-activated receptor 4 (PAR4) is activated by thrombin cleavage, revealing a tethered ligand that activates the receptor, making PAR4 a challenging target. A virtual screen of a make-on-demand chemical library yielded a one-hit compound. From the single-hit compound, we developed a novel series of PAR4 antagonists. Subsequent lead optimization via simultaneous virtual library searches and structure-based rational design efforts led to potent antagonists of thrombin-induced activation. Interestingly, this series of antagonists was active against PAR4 activation by the native protease thrombin cleavage but not the synthetic PAR4 agonist peptide AYPGKF.

## PAR4 as a Therapeutic Target

Thrombin, the major activator of platelets, activates protease-activated receptors PAR1 or PAR4 through the cleavage of the extracellular domain, revealing a tethered ligand (TL).^1−3^ PAR1 is activated first by lower concentrations of thrombin, but PAR1 signaling is transient.^4^ PAR1 activation is required for the initiation of hemostasis, but as PAR1-mediated platelet activation generates platelet prothrombinase on its surface, local thrombin concentrations increase. When local thrombin concentrations are high enough to activate PAR4, thrombin signaling is predominated by PAR4. PAR4 signaling is more sustained than PAR1 and drives yet more thrombin generation, fibrinogen cleavage to fibrin, and microparticle formation.^5^

While PAR4 has been most noted for the activation of platelets, PAR4 is also known to be expressed on diverse cell types with PAR4 expression higher in the context of inflammation than in control conditions.^6−14^ PAR4 knockout mouse studies have demonstrated the role of PAR4 in neutrophil homing and invasion at the site of vascular insult.^6−8^ PAR4 contributes to tissue damage after ischemia reperfusion injury in animal models of both myocardial infarction^15^ and stroke,^16^ as well as in inflammatory bowel disease,^9^ lung inflammation and fibrosis,^6,10−12^ and arthritis.^13^

PAR1 has a greater affinity for thrombin than PAR4, but despite early clinical promise, the addition of vorapaxar (the only licensed PAR1 antagonist) to standard care failed to meet its primary efficacy outcome in patients with acute coronary syndrome and was associated with an excess of major bleeding, especially intracranial hemorrhage, in phase 3 clinical trials.^17,18^ Therefore, attention has since turned toward PAR4 as a potential thrombin receptor therapeutic target. Bristol–Myers–Squibb (BMS) developed PAR4 antagonists BMS-986120 (**1**) and BMS-986141 as antithrombotic agents with high potency and specificity for thrombin activation of PAR4 over PAR1.^19−21^ PAR4 antagonists are hypothesized to have a lower bleeding risk than PAR1 antagonists because PAR4 acts at a later stage in the platelet activation process than PAR14. Importantly, in a cynomolgus monkey arterial thrombosis model, BMS-986120 (**1**) demonstrated potent and highly efficacious antithrombotic activity.^19^ BMS-986120 (**1**) also exhibited a low bleeding liability and a markedly wider therapeutic window compared to that of clopidogrel tested in the same nonhuman primate model. In phase I human clinical trials, BMS-986120 (**1**) and BMS-986141 were safe and well tolerated in healthy participants over a wide range.^20,22^ However, at this time, the BMS compounds have not advanced further in clinical development and larger clinical trials would ultimately be needed to establish safety.

## Targeting PAR4

**Figure 1:**
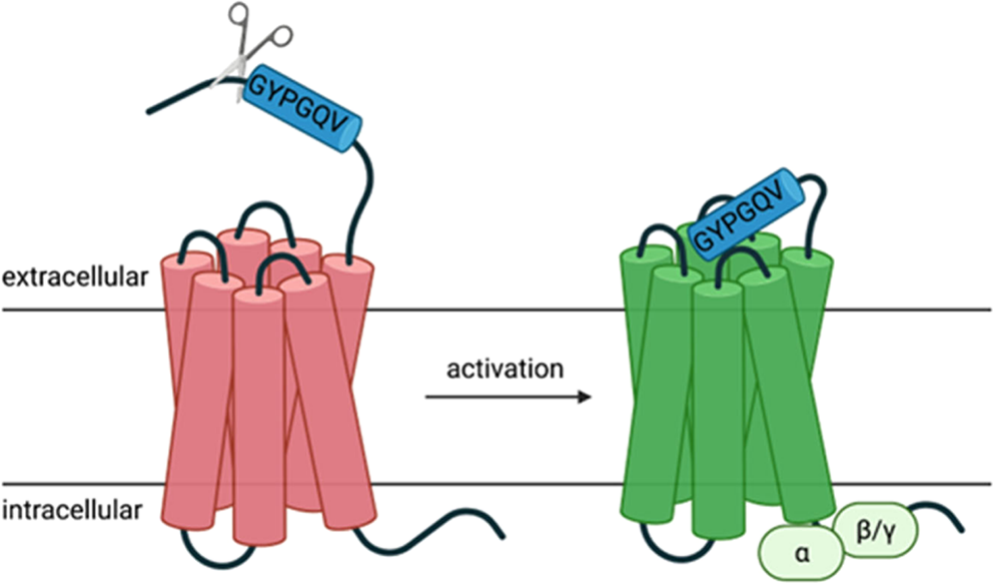
PAR4 activation mechanism. PARs contain the highly conserved seven transmembrane helical bundle, an extracellular N-terminus, and an intracellular C-terminus, which binds to respective G-proteins to initiate downstream signaling. PAR activation is caused by thrombin-induced cleavage between residues Arg47/Gly48 on the exposed extracellular N-terminus, revealing a new N-terminus called the “tethered ligand (TL)”. The TL subsequently binds within the 7TM helical bundle to induce a conformational change to the receptor, prompting G-protein binding and propagate downstream signaling via Gq and G_12/13_.

Unlike most other GPCRs, PARs are not activated by the binding of a soluble ligand. Instead, they are triggered by proteases, which cleave a part of their N-terminus, exposing a new N-terminus that we refer to as the “tethered ligand” that binds intramolecularly to the receptor, activating it and inducing signal transduction (Figure 1). Experimentally, PAR4 can be activated either by the native protease thrombin cleavage event or by a synthetic soluble agonist peptide mimicking the tethered ligand (“PAR4-AP”).

**Figure 2:**
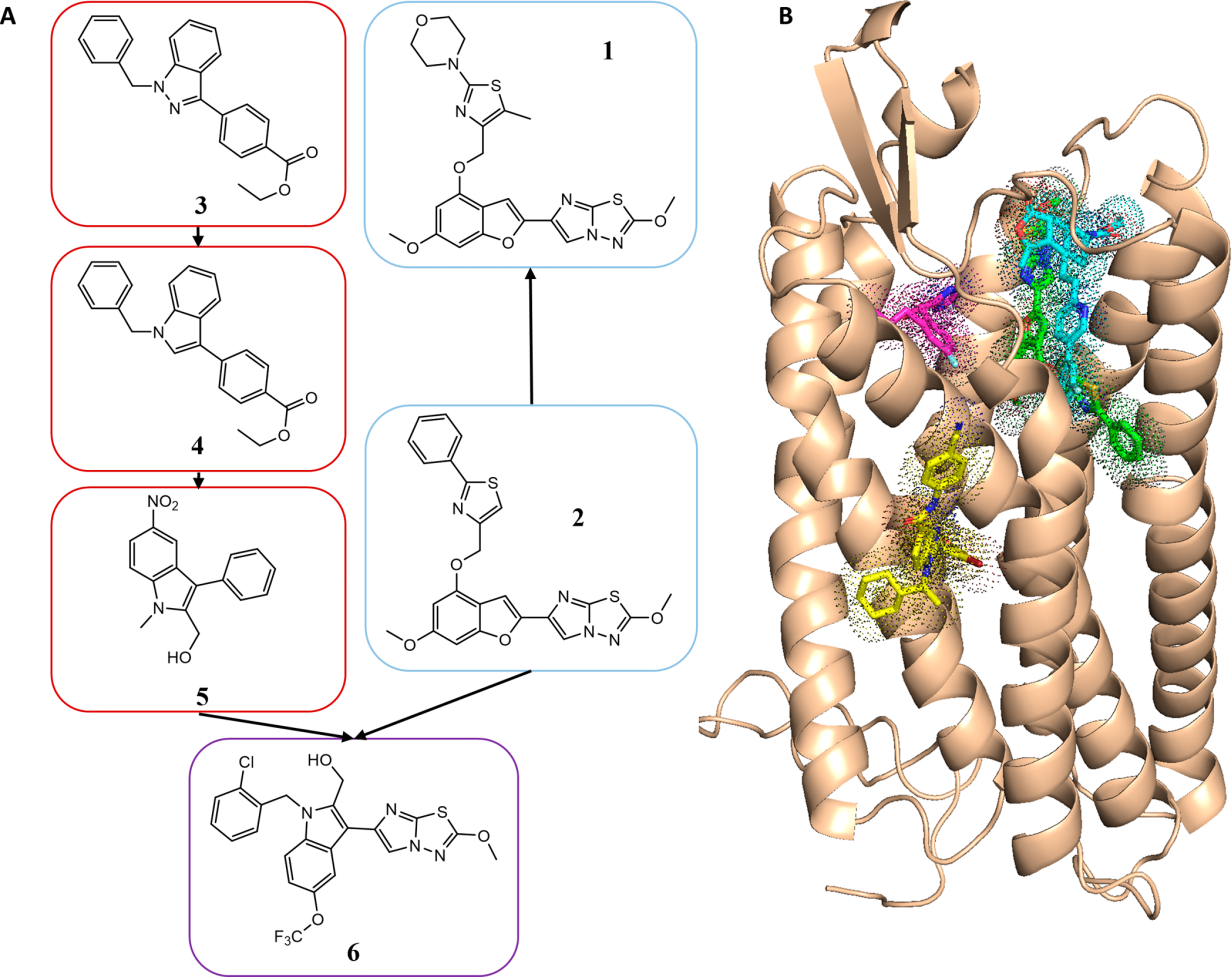
Previously identified PAR4 antagonists. (A) Red boxes follow lead optimization of **3** through **4** and **5** indole series; light blue designates imidazothiadiazole series from BMS starting with BMS-3 (**2**) from the HTS and BMS-986120 (**1**) in clinical trials; purple designates the chimerization series of the indole and imidazothiadiazole series (**6**). (B) Predicted binding mode of BMS-3 (green). Comparison to experimentally determined binding modes: vorapaxar (PAR1, PDB ID: 3vw7, cyan), AZ3451 (PAR2, PDB ID: 5NDZ, yellow), and AZ8838 (PAR2, PDB ID: 5NDD, magenta).

This unusual mechanism of action of PARs poses significant challenges in small molecule antagonist development. The native tethered ligand activator, being the newly cleaved N-terminus of the PAR, effectively exists at a high local concentration around the PAR binding site (estimated as high as 0.4 mM), making these receptors difficult to inhibit.^22,23^ Several groups have successfully inhibited agonist peptide PAR4 activation including an indazole scaffold,^24^**indole derivatives**,^25−27^ and a CNS-penetrant series;^28^ however, these compounds do not robustly inhibit the physiologically relevant thrombin activation of PAR4. Bristol–Myers–Squibb published a patent describing a series of efficacious and bioavailable PAR4 antagonists (WO2013163244). The initial hit (Figure 2A, BMS-3 (**2**)) from the BMS campaign contains an imidazothiadiazole scaffold and is a selective and potent PAR4 thrombin antagonist. We synthesized this lead as a tool compound, “BMS-3” (**2**).^4^ Chimerization of the imidazothiadiazole and indole (**6**) series has demonstrated weak antagonism against γ-thrombin (IC_50_ = 4.35 μM)^29^ (Figure 2A). Specifically, using the minimum pharmacophore of **2** to be the imidazothiadiazole moiety^29^ and systematic truncation of this molecule revealed a change in inhibition mode from noncompetitive to competitive.^4^

There currently exist no experimentally determined structures of PAR4; however, extensive in silico modeling studies using homology models of PAR4 in combination with mutagenesis and known antagonists led us to propose a binding mode for BMS-3. Here, we structurally probe “make-on-demand” chemical space for novel compounds that can extend into this antagonist binding pocket.

## Results and Discussion

Despite not having an experimentally determined structure of PAR4, there currently exist structures of homologous proteins that can be used as templates for comparative modeling of PAR4. Here, we used deposited Protein Data Bank^30^ crystal structures of PAR2, PAR1, chemokine receptor type 9 (CCR9), apelin receptor (APJ), and C5a receptor to build our model of PAR4. Templates were chosen based on the RosettaGPCR framework, which takes both sequence and structure-based GPCR alignments to select the optimal experimentally determined structure for individual regions to be used in multitemplate modeling.^30^ Additionally, we chose to include only other endogenous peptide binding GPCRS to maintain a larger binding pocket and include a conserved ECL2 β turn motif. In addition to using the respective protein templates, we built the PAR4 binding pocket around the determined binding mode of vorapaxar within PAR1 to generate the PAR4 structural model. Using this homology model and docking studies, we predicted that BMS-3 binds in a pocket overlapping with vorapaxar in PAR1^22^ while extending further down into the transmembrane bundle (Figure 2B). Also shown for comparison are the structures of two antagonists against PAR2.^31^ Independent findings based on hydrogen–deuterium exchange experiments demonstrate the mechanism of binding of the TL within the TM3/TM7 pocket, specifically noting the importance of T153 in the peptide binding event.^32^ Our modeling studies of BMS-3 series demonstrate that the minimum pharmacophore binds near the residues involved in TL binding proposed by Han et al.,^32^ whereas the extended ring system of BMS-986120 (**1**) binds deeper into the TM bundle outside of the TL binding pocket (Figure 2B). As the imidazothiadiazole scaffold is the most effective compound against TL activation, we aimed to structurally probe for a new chemical space that can extend into this pocket.

Using this hypothesis, we demonstrate a structure-based virtual ultralarge library screen using DOCK to identify a new series of PAR4 antagonists. This method has shown previous success in identifying novel binders where the structure of a known active compound bound to the receptor has been experimentally determined, specifically AmpC β-lactamase, D_4_ dopamine receptor,^33^ and melatonin receptor MT_1._^33,34^ Using comparative modeling in Rosetta^30,35^ to obtain a structure for docking simulations and the lead-like subset from the ZINC database (https://zinc.docking.org/),^36−38^ we screened 164 million compounds using the DOCK software to identify compounds distinct from the known PAR4 antagonist chemical space.^33,39,40^ After identifying and validating a single-hit lead compound, we developed a series of analogs using the make-on-demand library and medicinal chemistry rational design.

**Figure 3:**
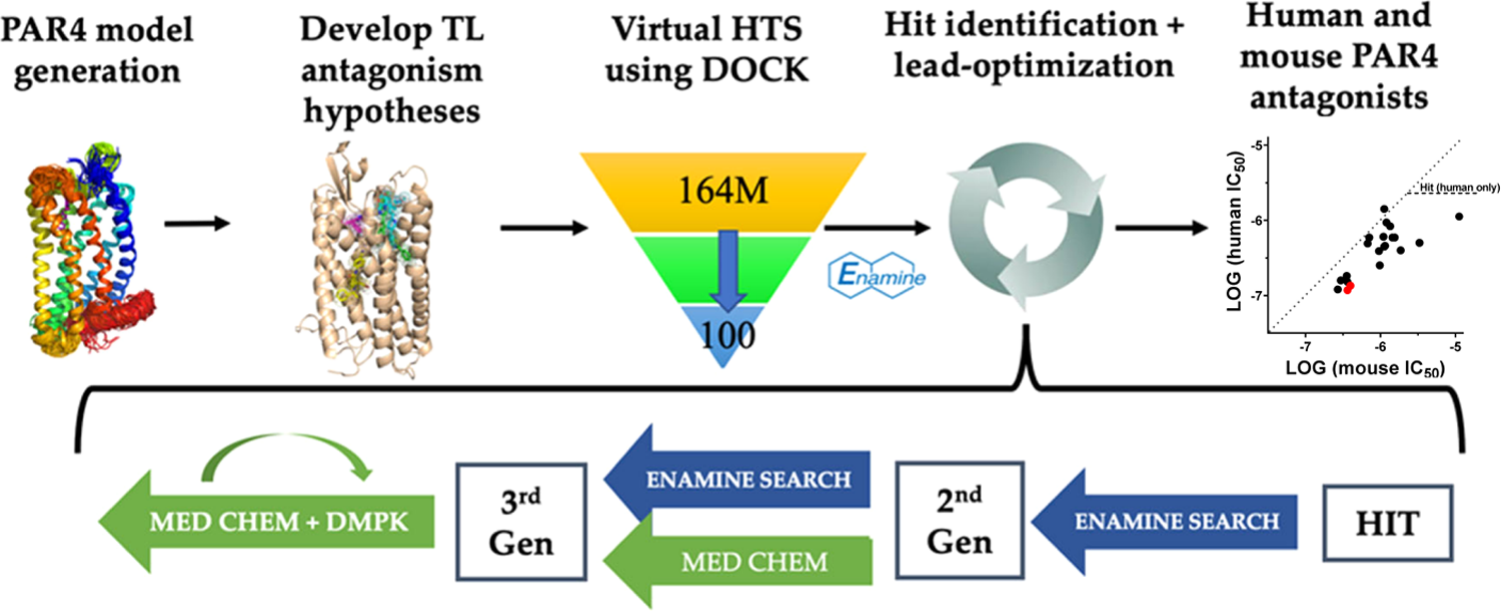
Overview of the structure-based high-throughput screen and subsequent optimization.

Parallel testing in both human and mouse platelets explores new tool compounds for *in vivo* mouse studies as well as optimization for clinically relevant human PAR4 antagonists. Aside from testing for activity at mouse PAR4, this parallel testing in both human and mouse platelets with structural mapping of sequence variations was used as an additional structure-based approach for optimization. This pipeline is summarized in Figure 3.

The initial virtual high-throughput screen of our PAR4 homology model was conducted, utilizing the lead-like subset of the ZINC ultralarge virtual library, composed of 164 million lead-like compounds. Virtual hit compounds were further filtered by DOCK score, transmembrane depth, visual analysis by medicinal chemists, and other chemical parameters. From the results of the virtual screen, 88 compounds were ordered from the Enamine make-on-demand library, and 79 were synthesized successfully.

**Figure 4:**
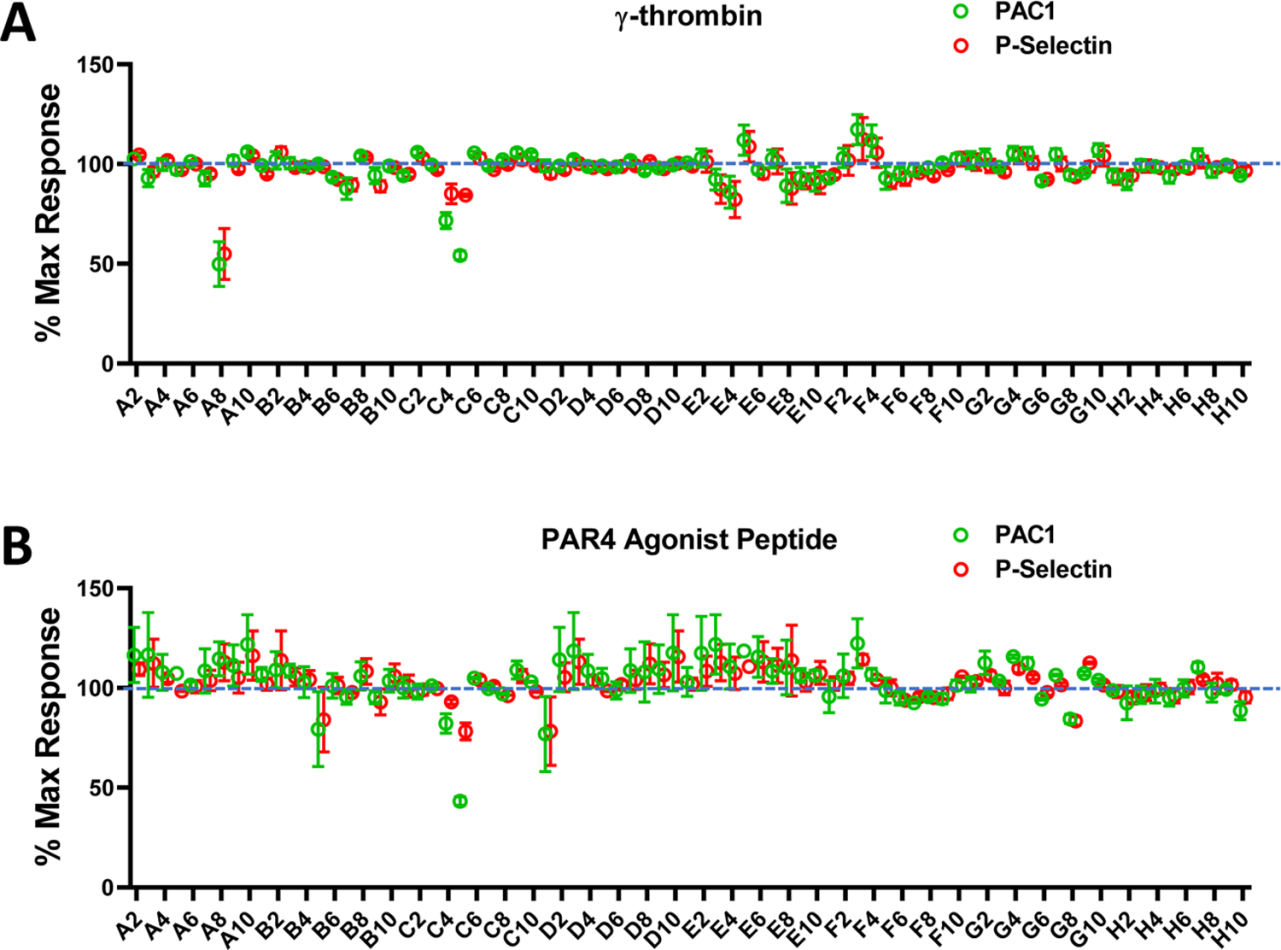
PAR4 platelet screen of enamine compounds generated from the vHTS conducted off the PAR4 homology model. Platelets activated 100 nM γ thrombin (A) or 200 μM PAR4 agonist peptide (AYPGKF) (B) with each compound normalized to vehicle DMSO control.

To test the antagonistic effect of the compounds on PAR4, a flow cytometry assay of human platelets was employed. Each compound was screened at 10 μM in human platelets using flow cytometry for GPIIbIIIa activation (PAC1) and P-selectin (CD62p) expression as previously described.^4^ Activation of GPIIbIIIa allows interaction with divalent fibrinogen or multivalent von Willebrand factor, leading to platelet aggregation.^41^ PAR4 stimulation also triggers α-granule secretion containing P-selectin.^5^ GPIIbIIIa and P-selectin flow cytometry signals exhibit strong correlation as readouts of PAR4 activation in platelets,^4,26^ and therefore, antagonists of PAR4 are expected to reduce PAC1 (active GPIIbIIIa) and P-selectin (CD62p) detected by flow cytometry in platelets. Platelets were treated with each test compound or vehicle control DMSO before activating with γ-thrombin (100 nM) or PAR4 agonist peptide mimetic (200 μM, “PAR4-AP” AYPGKF) for 30 min. γ-thrombin activates PAR4 but not PAR1.^42^ This screen identified one validated partial antagonist (“A8” (**7**), ZINC590833518) inhibiting the platelet activation by ∼50% against the protease γ-thrombin (Figure 4A) but not inhibiting the PAR4 agonist peptide (Figure 4B).

**Figure 5:**
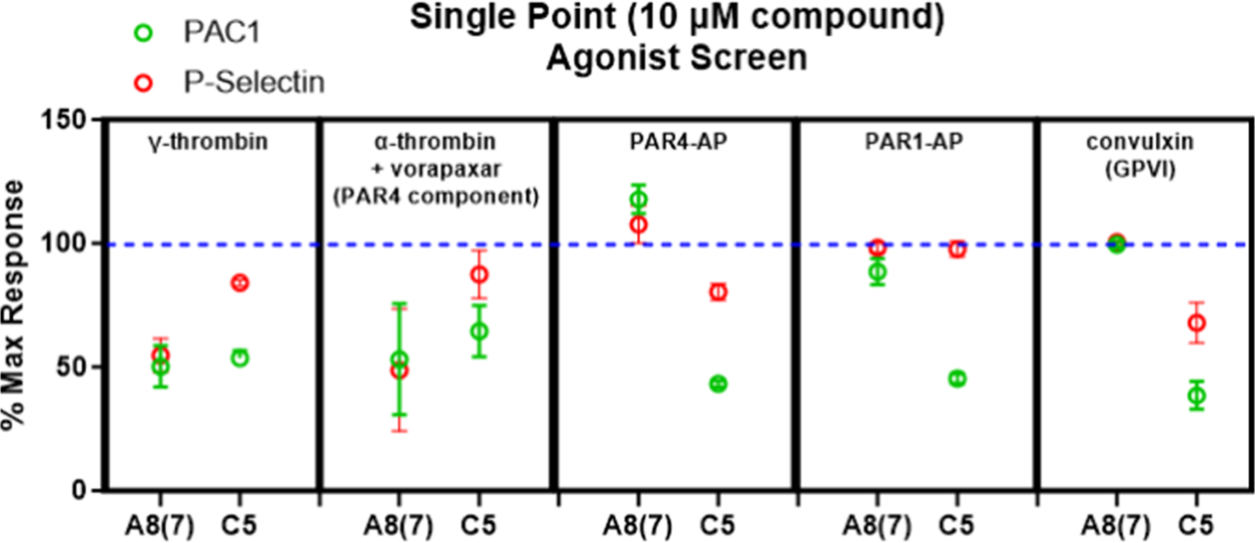
(A) Off-target controls: % of vehicle control for γ-thrombin (100 nM), PAR4 component of α-thrombin (5 nM α-thrombin +1 μM vorapaxar), PAR4 agonist peptide 200 μM AYPGKF, 200 μM PAR1 agonist peptide SFLLRN, and GPVI agonist convulxin 3.16 nM.

The hit compound (**7**) was tested against several platelet activators including targeting PAR1, PAR4, and GPVI collagen receptors (Figure 5). **7** did not inhibit the platelet off-target convulxin activation of the GPVI receptor. **7** also did not inhibit the synthetic agonist peptides for PAR4-AP or PAR1AP but did inhibit the PAR4 component of α-thrombin activation defined as a response to thrombin in the presence of the PAR1 antagonist vorapaxar (Figure 5). One other compound from the screen (“C5″, ZINC348291734) partially inhibited γ-thrombin activation but failed the off-target screen by also inhibiting platelet activation by the GPVI receptor agonist convulxin (Figure 5). C5 also displayed differential activity against PAC1 (green) and P-selectin (red), which our group has previously observed to be an effect of off-target inhibition of convulxin, whereas PAR4-specific antagonists inhibit both readouts of PAR4 signaling with similar efficacy. Taken together, the inhibition of the off-target GPVI and the divergence of the inhibition of the γ-thrombin readouts PAC1 vs P-selectin are consistent with the C5 compound not being a specific antagonist of PAR4.

**7** failed to directly inhibit thrombin in a fluorometric assay of in vitro thrombin activity, suggesting that **7** is inhibiting the PAR4 receptor rather than directly inhibiting thrombin enzymatic activity (Figure S1). The enamine-purchased **7** compound was confirmed using mass spectrometry and ^1^H NMR (data not shown) and then was independently resynthesized at Vanderbilt University (VU). The concentration–response curve (CRC) for **7** displayed an IC_50_ value of 2.3 μM (Figure S2B). These data show that at a higher concentration, **7** is a near-complete inhibitor for the γ-thrombin activation of PAR4.

Although it has a relatively low potency, **7** allowed for a starting point for structure–activity relationship (SAR) to be performed to improve potency. **7** (MW: 344 g/mol) is smaller than the clinical candidate compound BMS-986120 (**1**) (MW: 513 g/mol). The proposed binding mode of this compound in the homology model shows penetration deep in the transmembrane helical bundle pocket, similar to the proposed BMS compound and the TL (Figure S2C). These findings suggest that there are structural determinants of inhibiting the TL of PAR4 distinct from those inhibiting activation by the agonist peptide. This is also notable as previous efforts using conventional chemical library screening for PAR4 antagonists were relatively successful against the agonist peptide, while the TL was more challenging to inhibit.^24−28^

### Hit Compound A8 Optimization and Structure–Activity Relationship Development

A parallel approach to antagonist development included both (1) common scaffold and similarity searches of the virtual make-on-demand library and (2) medicinal chemistry optimization (Figure S3).

Following the functional characteristics of **7**, an additional screen was conducted of the Enamine database for structural similarity to that of **7**. Compounds for this “Analog set 1” were selected based on **7** Tanimoto similarity from the make-on-demand Enamine library. Analog set 1 (Figure S3) resulted in 5 compounds with a <30% max response against thrombin (data not shown), and three of these compounds **9**, **10**, and **17** were shown to have improved IC_50_ in comparison to the hit **7** (Tables 1 and 2). Structurally, **7**, **9**, **10**, and **17** all contain benzene off the pyrazole *N* (-R_1_ group), which is connected to another triazole via an amide bond. The second substitution of the pyrazole (-R_2_ group) showed the greatest variation within the hits. Thus, both small groups and large, sterically demanding groups were tolerated as well as differences in polarity. This prompted us to search the Enamine database for all compounds containing this same core as well as explore SAR through medicinal chemistry efforts.

**Table 1:**
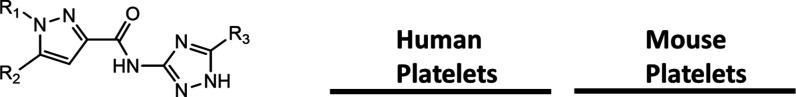

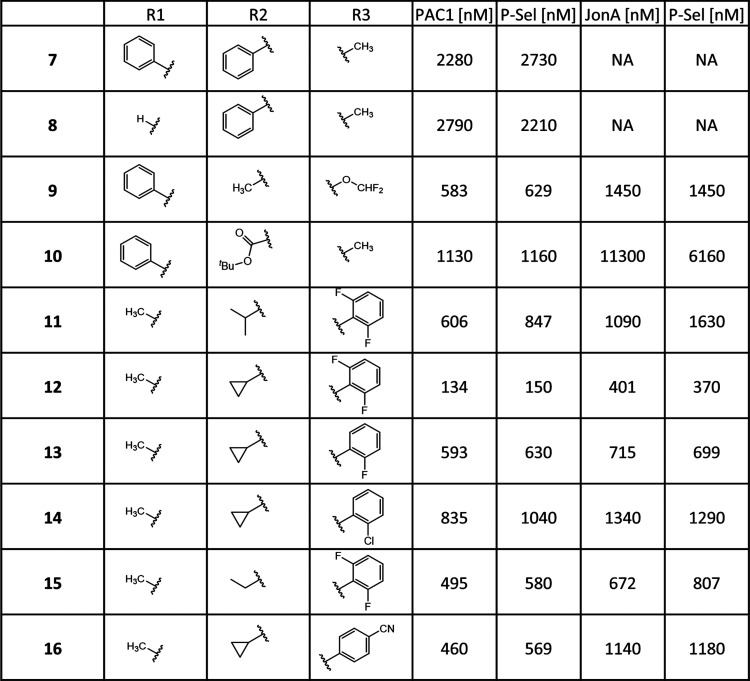
IC_50_ Values for Generation 1 Lead Optimization of **9** and **10**^a^

a PAC1 and P-selectin in human platelets, JonA and P-selectin in mouse platelets.

**Table 2:**
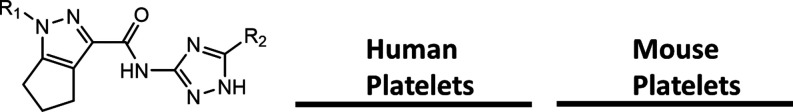

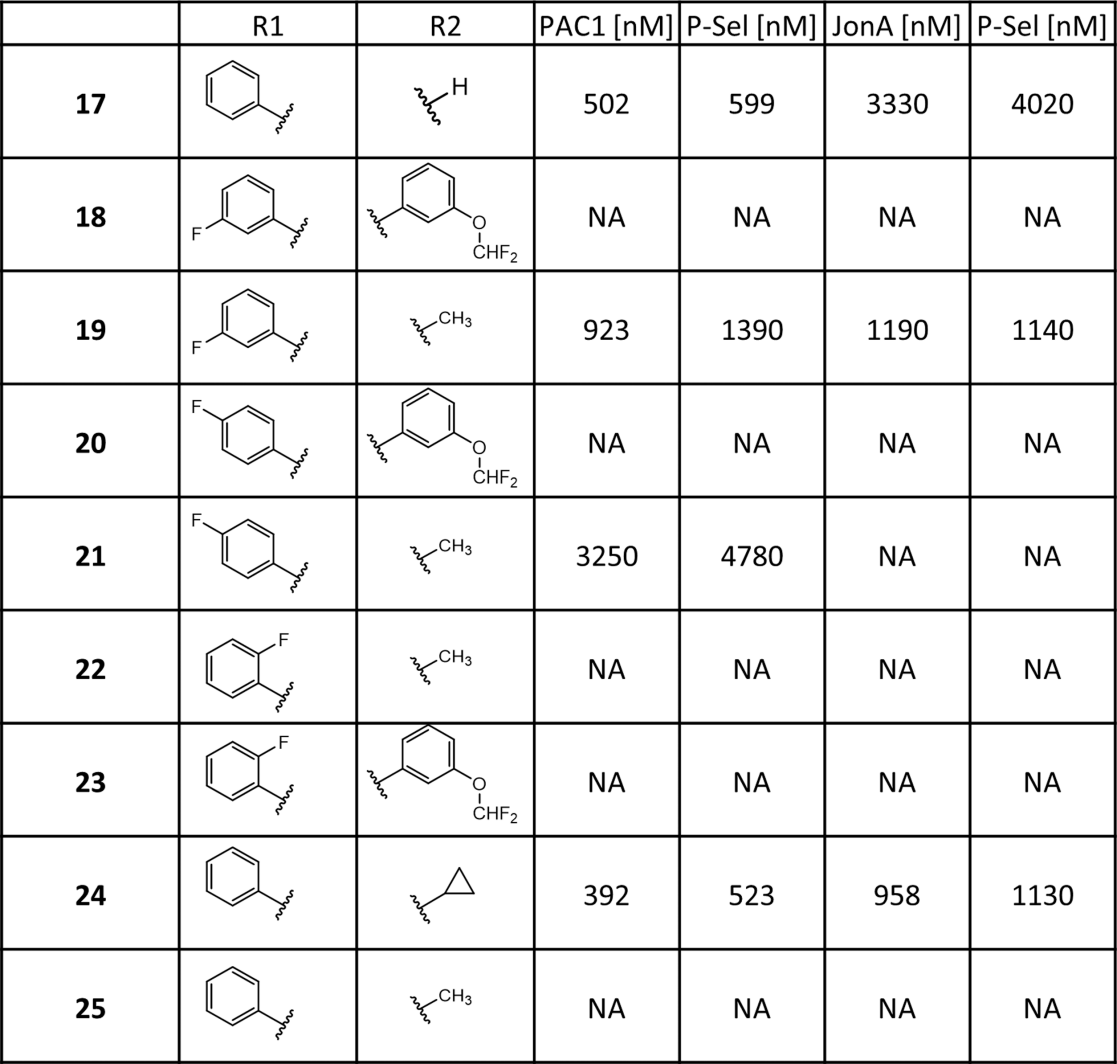
IC_50_ Values for Generation 2 Lead Optimization of **17**^a^

a PAC1 and P-selectin in human platelets, JonA and P-selectin in mouse platelets.

After screening enamine for similar compounds with the same core as the previous compounds, 100 compounds were selected for testing (80 synthesized successfully), which we have termed “Analog set 2”. Expectedly, this more specific search yielded a higher hit rate with 11/80 tested with similar or greater potency than that of **7** (Figure S3). We also explored derivatives from Analog set 1 around the common scaffold of **9** and **10** and separately around the fused ring moiety of **17** by searching through the Enamine database for similar compounds.

For **9** derivatives, compounds that showed comparable or improved activity had an -R_1_ substitution of a methyl group in place of the benzene and small hydrocarbon (isopropyl, ethyl, or cyclopropyl) substitution on -R_2_ showed overall improved activity over **9** with the exception of **14**. Compounds with an introduced aromatic ring on -R_3_ also showed improved potency (Table 1). **17** derivatives showed no activity upon the introduction of a difluoro-methoxy group on -R_2_. Halogen introduction at multiple sites around -R_1_ benzene led to a loss of activity (Table 2). Ring expansion of the fused ring system resulted in a cyclohexane series being the most potent series to date, leading to a nearly 2-fold IC_50_ improvement. Subsequent derivatization of -R_1_ and -R_2_ groups showed no obvious trends in halogen placement around the -R_1_ benzene or -R_2_ substitutions with different ring systems ranging from cyclopropane to *para*-cyanobenzene (Table 3). All efforts to replace the amide core with isosteric groups thus far have resulted in a loss of activity (data not shown). Replacing any triazole nitrogen with carbon or oxygen resulted in a complete loss of activity, as well (data not shown). Considering all medicinal chemistry efforts, a minimal pharmacophore consisting of a pyrazole linked by an amide bond and 1,2,4-triazole is obtained.

**Table 3:**
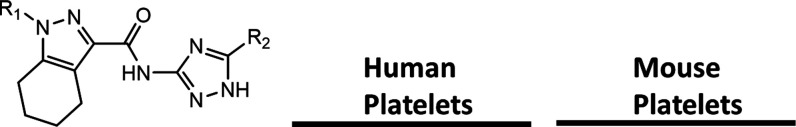

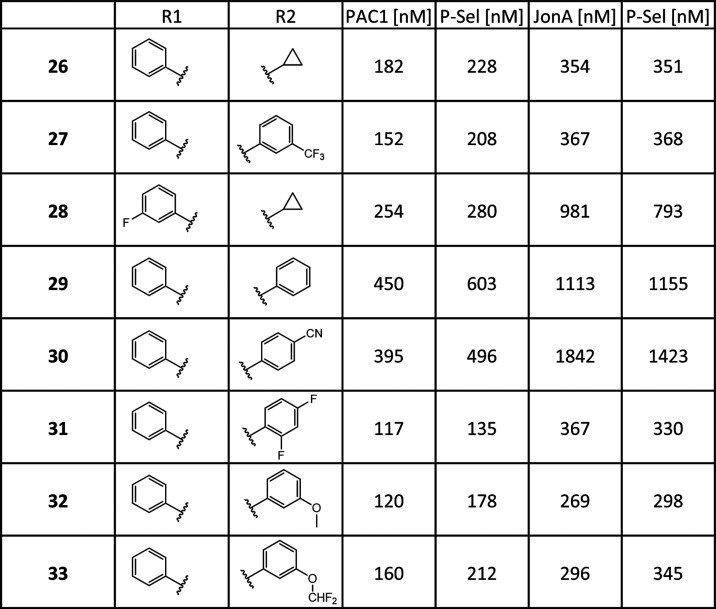
IC_50_ Values for Generation 3 Lead Optimization^a^

a PAC1 and P-selectin in human platelets, JonA and P-selectin in mouse platelets.

**Figure 6:**
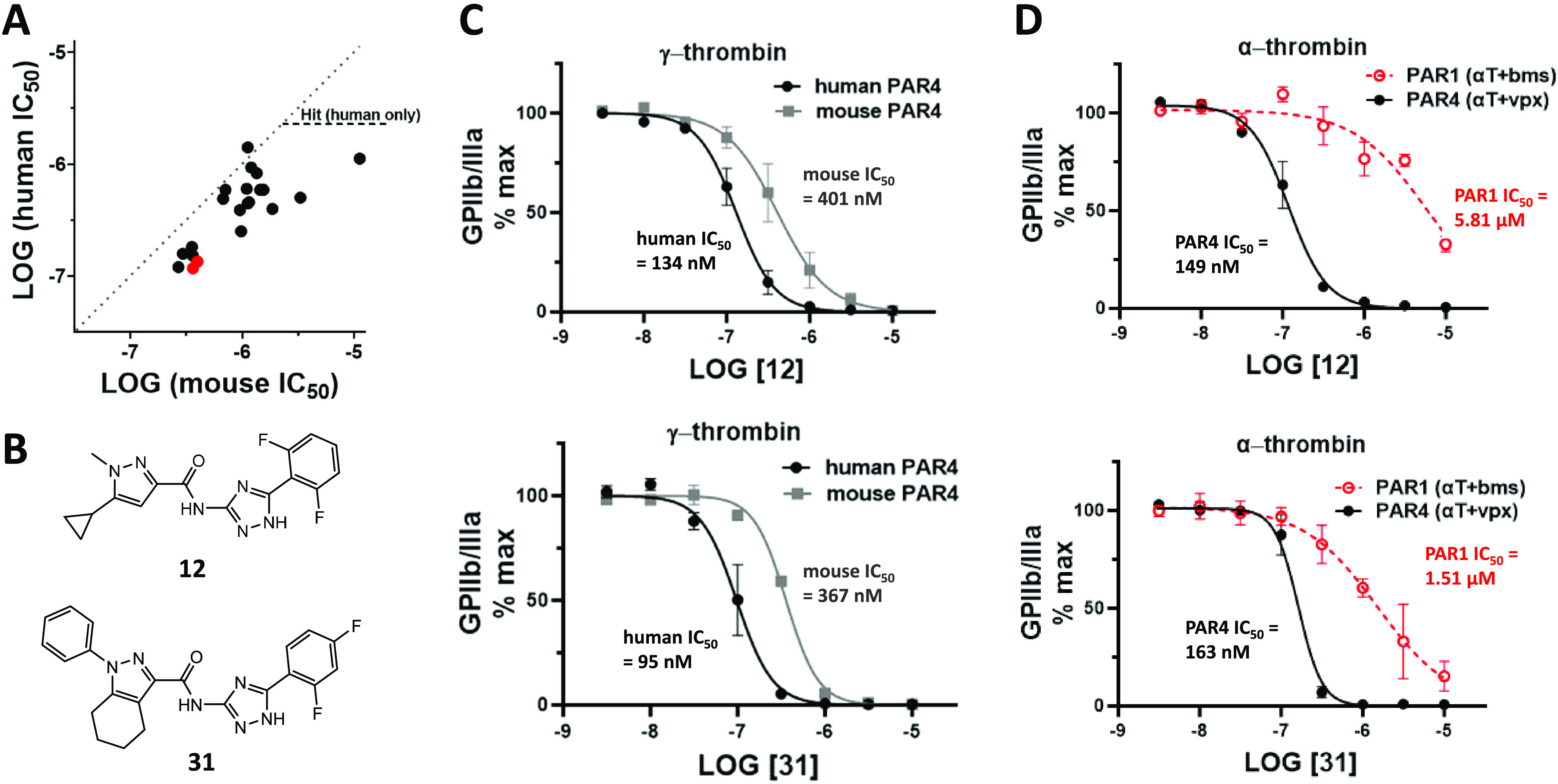
(A) Distribution of IC50 values for the inhibition of human PAR4 vs mouse PAR4. Red indicates the 2 compounds highlighted in panels (B–D). (B) Structures of exemplar compounds. (C) Concentration–response curves for human vs mouse PAR4. (D) Concentration–response curves for the inhibition of pharmacologically defined α-thrombin components for PAR1 (α-thrombin + BMS-3) vs PAR4 (α-thrombin + vorapaxar).

Figure 6A shows the progress and correlation of compound activity against human versus mouse platelets. The PAR4 sequence identity between the mouse and human is 78%, with only two residue variations (Q214H and A236E) within 5 Å of the binding pocket (Figure S4). As exemplars of this study, we compared key pharmacology properties of one potent compound from the virtual make-on-demand library (**12**) and one compound designed and synthesized locally (**31**) (Figure 6B). As is typical for the overall set of compounds (see Figure 6A), both **12** and **31** had modest potency preference for human over mouse PAR4 (Figure 6C). The basis for the potency preference for humans over mice is not known, as the predicted binding modes of **31** and **12** do not show direct interactions with the said residues. Aside from the Q214H and A236E mutations in the binding site, there are certainly other mutations that may be causing activity differences. There are roughly 30 mutations within the transmembrane domain likely causing distal changes in PAR4, which in turn affect receptor dynamics. Additionally, there are also mutations within the ECLs that may affect accessibility to the orthosteric site, as well as ICL mutations including helix 8 that potentially change the G-protein interface, causing signaling bias or affecting dynamics. Systematic mutational analysis of these regions in tandem with parallel signaling assays to determine the recruitment of different G-proteins or arrestin will be necessary to further understand the dynamics of PAR4 signaling.

α-thrombin activates both PAR1 and PAR4, and the individual PAR1 or PAR4 component can be isolated pharmacologically. Both **12** and **31** exhibit a preference for inhibiting PAR4 thrombin activity over PAR1 thrombin activity (Figure 6D). With 20 substitutions between PAR1 and PAR4 within 5 Å of the predicted **31** binding site, the basis for these selectivity differences is not known (Figure S4). As we continue to probe for a selective PAR4 antagonist, mutagenesis of individual residues will be conducted.

**Figure 7:**
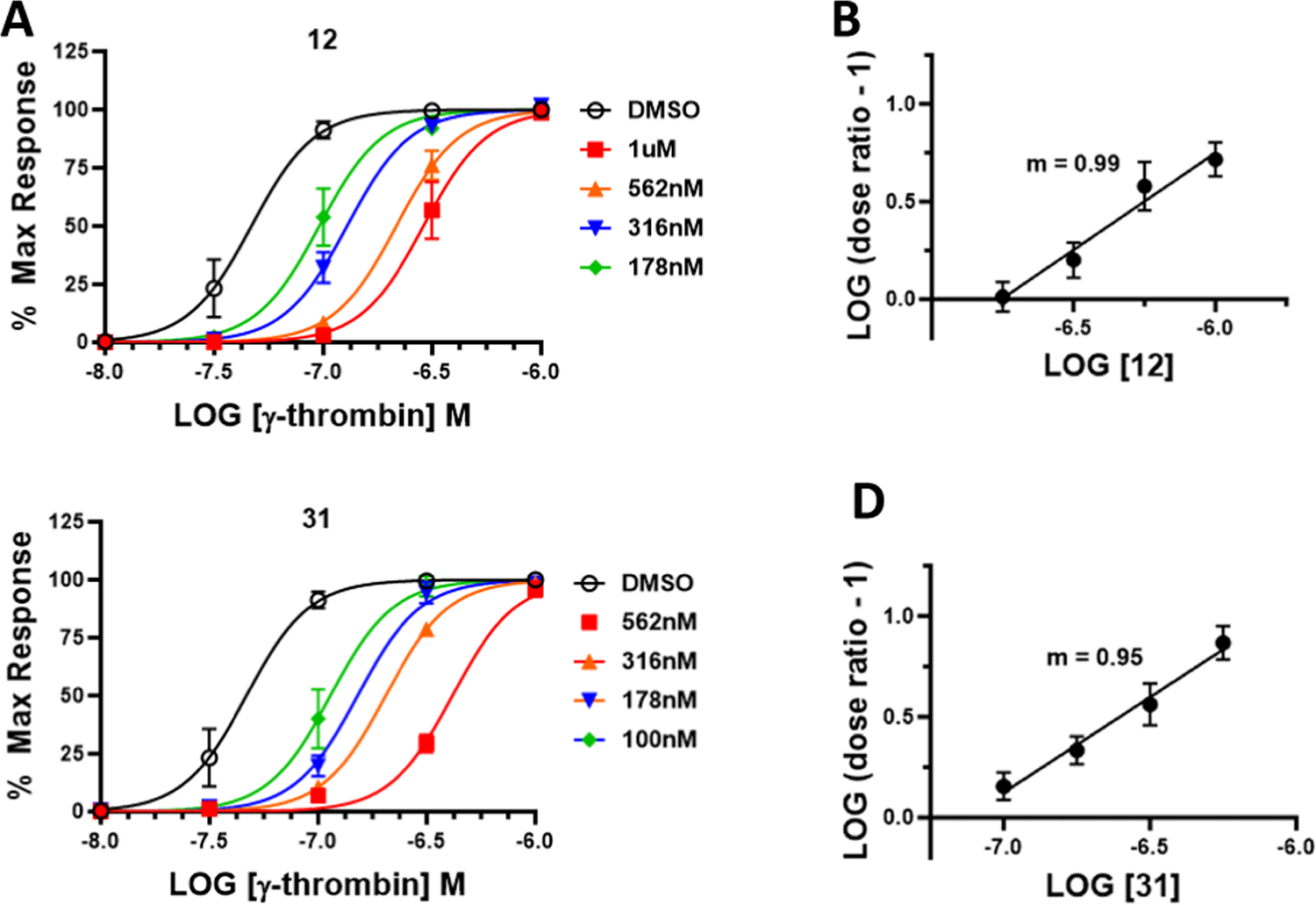
Schild analysis progressive fold-shift experiments. (A) Platelets were pretreated with increasing concentrations of each antagonist for 20 min prior to activation with increasing concentrations of γ-thrombin. (B) Logarithmic transformation of the dose ratio plotted versus the logarithm of compound concentration. *m* = slope of linear regression.

A Schild analysis was performed on compounds **12** and **31** to determine the mode of inhibition of these compounds. Both compounds exhibited a rightward shift in the Schild graph and a linear slope of about one on the Schild plot. This indicates that both compounds exhibit a competitive mode of inhibition against γ-thrombin activation (Figure 7).

### Structural Analysis of the Predicted Binding Modalities of the Antagonists

**Figure 8:**
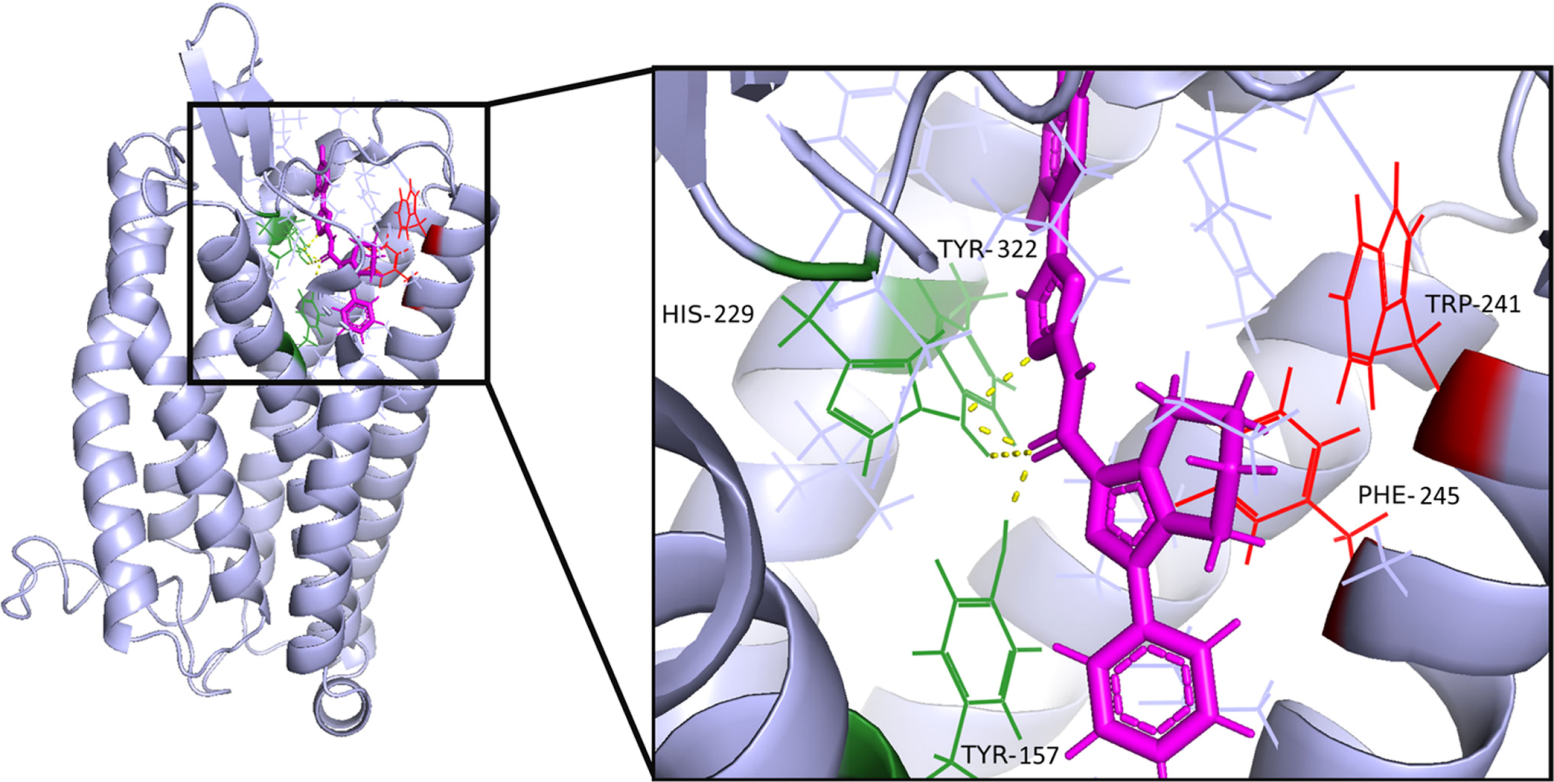
Predicted binding mode of **31** in the PAR4 homology model. Noting key binding interactions: H-bonds (yellow), H-bonding residues HIS162, TYR255, and TYR90 (green), and hydrophobic interactions TRP147 and PHE178 (magenta).

Structural analysis of our current most potent compound, **31**, provides a hypothesis for further derivatization to improve potency (Figure 8). The current model shows the carbonyl oxygen from the amide core of the molecule forming hydrogen bonds with the hydroxyl groups of Tyr157 and His229, while Tyr322 forms hydrogen bonds with the triazole nitrogen adjacent to the amide; the fused cyclohexane group occupies a hydrophobic pocket near Trp241 and Phe245, and the benzene from off the pyrazole nitrogen extends down into the TM domain. 1,3-Difluorobenzene off the triazole points into the extracellular region.

**Figure 9:**
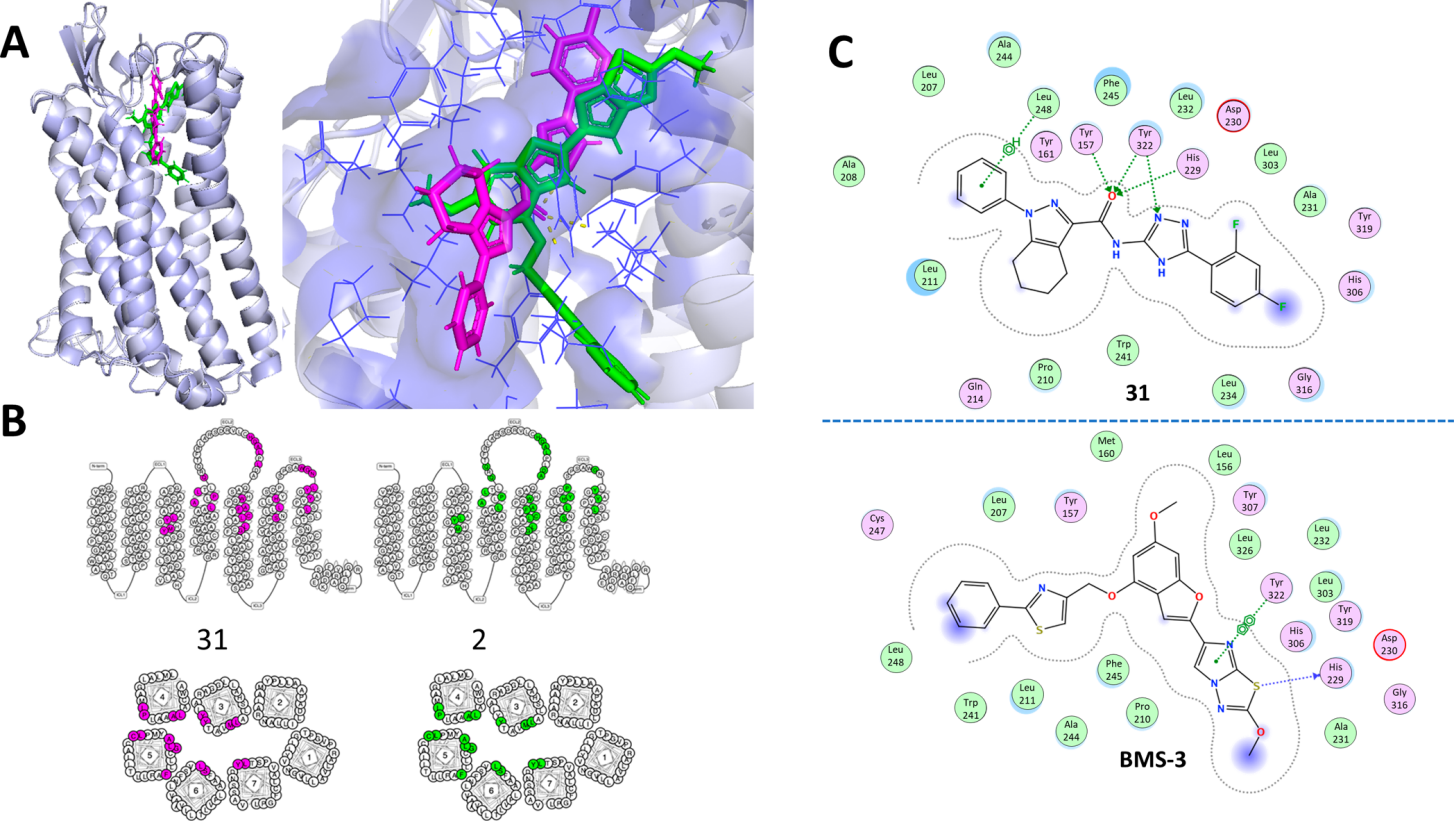
Predicted binding mode of **31** and BMS-3. (A) Predicted docking of **31** (magenta) and **2** (green) in the PAR4 homology model, noting the binding pocket in yellow. (B) Key residue of PAR4 interacting with **31** and **2**. (C) Binding interaction between key residues of PAR4 and **31** and **2**.

Overlaying **31** and BMS-3 (**2**) predicted binding modes shows that both molecules occupy the same pocket and interact with a nearly identical set of residues (Figure 9). A closer inspection of Figure 9A shows the triazole moiety overlaying the imidazothiadiazole of BMS-3 (**2**), whereas on the other side of the molecule, the phenyl off the pyrazole is overlaid onto the thiazole of BMS-3 (**2**). This supports previously published data, showing that the imidazothiadiazole of BMS-3 (**2**) is also the minimum pharmacophore.^4^ Whereas His162 and Tyr255 interact with the core amide oxygen, these same residues are predicted to interact with the imidazothiadiazole.

The ability of these compounds to inhibit the endogenous activation mechanism and not the peptide-mediated activation is still an important question to answer and requires more in-depth structural and signaling studies between the mechanisms of TL and PAR4-AP-induced activation. We propose two intertwined and testable hypotheses: the TL and PAR4-AP bind to separate sites or induce distinct structural receptor states upon binding, or the TL and PAR4-AP demonstrate distinct signaling patterns.

It has previously been shown that TL binding requires a complex arrangement of TM3, TM7, ECL2, and ECL3.^32^ It is possible that our molecules bind to a receptor state that occludes binding of the TL but not binding of the PAR4-AP. We specifically screened for molecules that bind deeper within the transmembrane domain, which is the proposed binding site for the TL, whereas the lower-affinity PAR4-AP could bind further toward the ECLs. Aside from interactions with the extracellular domain, the conformational states of the 6-residue soluble peptide and the corresponding TL residues likely differ, therefore potentially resulting in distinct binding mechanisms. Another explanation lies in the intracellular domain, specifically whether the TL and PAR4-AP demonstrate distinct signaling biases and kinetics. Structural changes on the intracellular side may induce changes in the recruitment of different G-protein or β-arrestin, which is not seen in our assay. Detailed signaling analysis using more direct activity readouts of the ligands may help us with our understanding of the activation mechanisms. In-depth structural and signaling studies including mutational analysis in both the presence and absence of thrombin and free peptide will be necessary for understanding these PAR4 activation mechanisms.

### Pharmacokinetic Properties of Top Compounds

To assess the druglikeness of some of these molecules, the in vitro intrinsic clearance (CL_lint_) and plasma protein binding (PPB) were measured in mouse and human matrices. The values associated with these initial measurements are listed in Table 4. Plasma protein binding was high in human plasma, ranging from 0.6 to 5.2% free. For the compounds measured in mouse plasma, the percent free ranged from 0.6% to 16.2%. The most potent compound, **31**, exhibited an intrinsic clearance of 459 mL/min/kg and 19,894 mL/min/kg in human liver microsomes (HLMs) and mouse liver microsomes (MLMs), respectively. In HLM, similarly high intrinsic clearance values were measured for all of the other compounds tested, with the exception of **17**, which returned a Clint value of 49.6 mL/min/kg. While this value is still high for translational developability of the molecule, it is distinct from the others that were measured. In vitro intrinsic clearances in MLM of **17** and **12** were 2443 and 11534 mL/min/kg, respectively. Given the high intrinsic clearance values, the calculated hepatic clearance values were all approaching liver blood flow values in both the human and mouse.

**Table 4:** In Vitro Drug Metabolism and Pharmacokinetic Parameters (*f*_u_, Unbound Fraction; PPB, Plasma Protein Binding)

			human (in vitro mL/min/kg)		mouse (in vitro mL/min/kg)		human PPB	mouse PPB
compound	human PAR4 IC50 (nM)	mouse PAR4 IC50 (nM)	Cl_int_	CL_hep_	Cl_int_	CL_hep_	*f*_u_	*f*_u_
**17**	502	3330	49.6	14.8	2443	86.8	0.029	0.006
**31**	95	367	459	20.1	19894	89.6	0.015	0.044
**12**	134	150	478	20.1	11534	89.3	0.052	0.169
**9**	583	1450	407	20	NR	NR	0.036	NR
**33**	160	296	416	20	NR	NR	0.006	NR
**32**	120	269	285	19.6	NR	NR	0.007	NR

It is of note that the experimental design for the initial round of microsomal clearance assays adds a test compound to microsomes before the reaction is started by the addition of nicotinamide adenine dinucleotide phosphate (NADPH). This design assumes that the major contributing drug-metabolizing enzymes are cytochrome P450s (CYPs). To investigate this further, the experimental design was modified where the reaction was initiated by the addition of a test compound and was carried out in the presence or absence of NADPH. The intrinsic clearances of **17**, **9**, and **12** were measured under these conditions (Table S1). By comparison of the measured intrinsic clearance in the presence and absence of NADPH, the contribution of non-CYP450 drug-metabolizing enzymes can be estimated.

Mammalian liver microsomes include CYPs, flavin monooxygenases (FMOs), glucuronosyltransferases (GSTs), and esterases as part of their enzyme composition. In the presence of NADPH, which serves as a cofactor for CYP- and FMO-dependent reactions, the observed activity can be attributed to these enzymes. Similarly, UGTs require the cofactor uridine diphosphate glucuronic acid (UDPGA). Therefore, when NADPH and UDPGA are absent, any reduction in the compound can be ascribed only to esterase activity or chemical instability. Incubations with **12**, **17**, and **31** in potassium phosphate (KPI) or Tyrode’s buffer (HP) only showed little loss of compound over time (Figures S5C,D, S6C,D, and S7C,D), so the loss of compound in the liver microsomal incubations in the absence of NADPH is likely due to esterase activity. These data support the notion that the in vitro potency measurements of the compounds are likely an accurate reflection of their inherent pharmacological activity at PAR4. This is based on two factors: (1) the stability of the compounds in HP buffer, which is used in the platelet assays, and (2) the low probability of drug metabolism occurring within the platelets themselves. Together, these minimize the chance that any metabolites with distinct PAR4 potencies could confound the results. By comparing the area under the curve of the compound metabolized from the microsomal incubations in the presence or absence of NADPH to the corresponding area under the curve of the buffer-only incubation, the percent contribution of CYPs and FMOs and esterases were estimated (Table S1). The major contributing enzyme toward the metabolism of **12** is esterases in both the human (96%) and mouse (98%). In contrast, the metabolism of **31** was primarily driven by CYPs and FMOs in both HLM (80%) and MLM (88%). Metabolism of **17** in MLM was solely attributed to the CYP/FMO activity, whereas in HLM, CYP/FMO contributed only 39%.

## Conclusions

This study has integrated multiple approaches to develop a potent PAR4 antagonist. Without an experimentally determined structure of PAR4, a virtual high-throughput screen was used on a homology model to generate an initial hit compound. Through subsequent computational screens and parallel medicinal chemistry efforts, SAR was conducted to generate several potent and specific PAR4 antagonists. Further SAR and optimization are ongoing to increase the potency and improve the DMPK properties. As thrombin is the endogenous activator of PAR4, it would be of interest to focus drug discovery efforts against the TL over the PAR4-AP. This series of compounds has antagonist effects against the endogenous activator of PAR4, thrombin, but not PAR4 activation by the agonist peptide mimetic (PAR4-AP, AYPGKF). The pharmacological differences between inhibition by thrombin and inhibition by the agonist peptide may be important for understanding the structural basis for the activation of PAR4 by the protease-generated tethered ligand.

## Materials and Methods

### PAR4 Model Generation

Despite not having an experimentally determined structure of PAR4, there currently exist deposited structures of homologous proteins to use for comparative modeling of PAR4. Here, we used deposited Protein Data Bank crystal structures of PAR2, PAR1, chemokine receptor type 9 (CCR9), apelin receptor (APJ), and C5a to build our model of PAR4. In addition to using the respective protein templates, we built the PAR4 binding pocket around the determined binding mode of vorapaxar within PAR1 to generate 500 output models.

Inclusion of a ligand in the binding site has been shown to be crucial during homology model building due to binding pocket collapse. It would be ideal to obtain a known PAR4 antagonist that is chemically close to a ligand cocrystallized in another template. First, we took all known PAR4 binders and ran Tanimoto similarity to the ligands cocrystallized in PARs 1 and 2. If we found a known PAR4 antagonist that was chemically similar to a cocrystallized PAR1 or PAR2 ligand, we could align the compounds and use this in RosettaCM; however, there was no significant chemical overlap. We then looked at all known binders to PARs 1, 2, and 4 from ChEMBL and ran Tanimoto similarities to determine any chemical overlap; however, we still found nothing. We also tried breaking down vorapaxar into fragments and compared to known PAR4 antagonists, but there was still no chemical overlapping. We then decided to use the predicted binding mode of BMS-3 aligned with the templates to build PAR4 using RosettaCM. Tanimoto similarities in all cases were <0.3, and binder threshold was defined using a threshold IC_50_ < 200 nM.

We used all 500 receptor models from the comparative modeling step for receptor optimization by testing the enrichment rates when scoring using the DOCK algorithm when docking known binders and property-matched generated decoys from the DUD-E database. Looking at the docked structures of the known active compounds, while taking into account the enrichment of these actives, we selected a single PAR4 model to move forward into the ultralarge screen. From the ZINC database,^37^ we opted to screen against the lead-like compound set containing 129 million compounds using the following criteria: MW: 300–350 Da, log *P*: −1–3.5, Rep: 3D, React: Standard, Purch: Wait OK, pH: ref Mid, Charge: 0. (Data set curated on December 4, 2019.)

### Postscreen Filtering

An arbitrary cutoff of −40 DOCK score was used as a cutoff value for initial filtering; this resulted in ∼100 K DOCKed poses and is only the top ∼0.4% of screened compounds. Using Tanimoto similarities, we removed all compounds >0.35 Tc to known PAR4 binders, which reduced our compounds to ∼99.7 K. We then ran a filter that only selects compounds that docked below a certain threshold with respect to the membrane, resulting in ∼7000 remaining compounds to analyze. The transmembrane depth filter was put in place based on our hypothesis that we want a binder that penetrates deep into the transmembrane bundle to inhibit the TL mechanism. To ensure that compound poses are energetically favorable and below a certain internal strain threshold, we ran the resulting compounds through a conformer analysis with a threshold <1.5 TEU (arbitrary torsional strain units) and resulted in 4638 compounds. These compounds were then clustered based on the Tanimoto similarity to ensure we were testing a diverse set of compounds, resulting in 2101 cluster heads for further inspection. The resulting compounds with their respective predicted binding pose from the DOCK simulation were analyzed for interaction criteria using Rosetta’s InterfaceAnalyzer application, namely, to quantify the number of unsatisfied hydrogen bonds and the number of hydrogen bonds between the ligand and receptor.^43^ This interface analysis was meant to guide visual inspection of the 2101 output models, in which we also manually inspected predicted interactions. All command-line protocol captures for these steps are detailed in the Supporting Information.

### Hit-to-Lead Optimization Using SAR-by-Purchase Approach

To acquire “Analog set 1”, we screened the ZINC database for compounds similar to A8. As we were starting with a single hit, we wanted the next set to balance having enough similarity to our initial hit while obtaining a rich diversity of compounds to expand potential SAR. We screened the ZINC library to acquire compounds with a Tanimoto similarity of >0.75 relative to A8. These compounds were then clustered based on a Tanimoto similarity threshold of >0.75 with respect to each other to achieve a maximally diverse subset, resulting in 207 compound clusters. The compound with the lowest molecular weight from each of these clusters was selected to serve as a potential starting compound for testing and to be easier for subsequent optimization. These 207 resulting cluster representatives were aligned to the predicted binding mode^44,45^ for **7**, redocked using RosettaLigand,^46−48^ and subsequently ranked based on score, number of unsaturated hydrogen bond donors and acceptors, and number of interface hydrogen bonds formed. These compounds were first screened at 10 μM; the dose–response curves were performed for the compounds that were full antagonists at this concentration. Of these 80 compounds, three had higher affinities than the original hit. All three analogs passed the same off-target screen we performed for the original hit, not inhibiting platelet activation by the GPVI receptor agonist convulxin nor the PAR1 agonist peptide (data not shown, *n* = 3).

“Analog set 2” was based on the common core of the resulting 3 higher affinity compounds. We rescreened the Enamine database for compounds containing this chemical motif (SMILES string: “c1*n*c(NC(=O)c2cc[*n*H]*n*2)*n*[nH]1”). This search resulted in 100 compounds that we then purchased from Enamine for testing. We also explored analogs around the Analogs set 1 hits 212B10, 212D8, and 212H3 individually. We repeated the steps above from A8 to obtain derivatives for B212D8 and B212H3, driving a 20-fold increased potency.

### Chemicals and Reagents

All solvents were purchased from Sigma-Aldrich and were used without further purification. Unless otherwise stated, all reagents were purchased from commercial suppliers and used without further purification. ^1^H and ^13^C NMR spectra were recorded on a 400 MHz AMX Bruker NMR spectrometer and referenced internally to the deuterated solvent signal. Chemical shifts are reported downfield in δ-values (ppm). The signals are reported as follows: chemical shift, multiplicity (s = singlet, bs = broad singlet, d = doublet, t = triplet, q = quartet, dd = doublet of doublets, m = multiplet), coupling constant, and integration. Reversed-phase LCMS analysis was performed using an Agilent 1200 system composed of a binary pump with a degasser, a high-performance autosampler, a thermostated column compartment, a C18 column, a diode-array detector (DAD), and an Agilent 6150 MSD with the following parameters. The gradient conditions were 5–95% acetonitrile with the aqueous phase 0.1% TFA or 1 g NH_4_CO_3_/L in water over 1.4 min. Samples were separated on a Waters Acquity UPLC BEH C18 column (1.7 μm, 1.0 mm × 50 mm) at 0.5 mL/min, with column and solvent temperatures maintained at 55 °C. The DAD was set to scan from 190 to 300 nm, and the signals used were 220 and 254 nm (both with a bandwidth of 4 nm). The MS detector was configured with an electrospray ionization source, and the low-resolution mass spectra were acquired by scanning from 140 to 700 AMU with a step size of 0.2 AMU at 0.13 cycles/second and a peak width of 0.008 min. The drying gas flow was set to 13 L/min at 300 °C. and the nebulizer pressure was set to 30 psi. The capillary needle voltage was set at 3000 V, and the fragmentor voltage was set at 100 V. Data acquisition was performed with Agilent Chemstation and Analytical Studio Reviewer software. The purity of all compounds was greater than 95% according to LCMS. The starting benzamidoguanidines were synthesized according to.^49^ The cyclization to the 3-amino-1H-1,2,4-triazoles was performed according to.^50^ Fluorine containing carboxylic acids was prepared from the appropriate fluoroanilines according to.^51^

### General Procedure for the Amide Coupling of the Final Compounds

Final compounds were prepared through a mixture of the carboxylic acid (1 equiv), triethylamine (1.5 equiv), 1-ethyl-3-(3-dimethylaminopropyl)carbodiimide hydrochloride (1.5 equiv), 4-dimethylaminopyridine (1.5 equiv), and the corresponding 3-amino-1H-1,2,4-triazole (1.5 equiv) in dry acetonitrile (0.5 mL/0.1 mmol), a mixture of the carboxylic acid (1 equiv), triethylamine (1.5 equiv), hexafluorophosphate azabenzotriazole tetramethyl uronium (HATU) (1.5 equiv) in dry dimethylformamide (DMF) (0.5 mL/0.1 mmol), or a mixture of the carboxylic acid (1 equiv), triethylamine (1.5 equiv), 2-chloro-1-methylpyridinium iodide (1.2 equiv), and the corresponding 3-amino-1H-1,2,4-triazole (1.5 equiv) in dry dimethylformamide (DMF) (0.5 mL/0.1 mmol). The reactions were stirred overnight at room temperature. The solvent was then removed under pressure, and the crude reaction was purified on silica.

### Human Platelet Activity Assay

Activation of washed human platelets was monitored by flow cytometry for the detection of PAC1 (GPIIbIIIa activation) and CD62p (P-selectin expression) binding as previously described.^4^ Human platelets were obtained from healthy volunteers. The Vanderbilt University Internal Review Board approved these studies. Informed consent was obtained from all individuals prior to the blood draw. 10 mL of whole blood was collected into 0.32% Na citrate. Platelet-rich plasma was collected after room-temperature centrifugation at 1100 rpm for 15 min on a Thermo Forma 400 centrifuge (Thermo Fisher, Waltham, MA). 1/10 volume of acid citrate dextrose (ACD) buffer was added to the collected supernatant and incubated for 10 min. After centrifugation at 2400 rpm for 10 min, the platelet pellet was resuspended in 1 mL Tyrode’s buffer (“TBB”: 15 mM HEPES, 0.33 mM NaH_2_PO_4_, pH 6.4, 138 mM NaCl, 2.7 mM KCl, 1 mM MgCl_2_, 5.5 mM dextrose, 0.1% bovine serum albumin). Platelets were collected, counted on a Z1 Coulter Particle Counter (Beckman Coulter, Brea, CA), and diluted in TBB to 1.5 × 10^7^ platelets/mL. 60 uL of platelets were aliquoted to each tube and preincubated with 40 μL of TBB with antibodies PAC1 FITC (Becton Dickinson, Franklin Lakes NJ) and P-selectin PE (Becton Dickinson) and either test compound antagonist or DMSO vehicle control for 20 min. Platelets were then activated with 100 nM γ-thrombin or other agonists for 30 min. Samples were then fixed by adding 100 μL of 2% paraformaldehyde. After 30 min, samples were diluted by adding 300 μL of phosphate-buffered saline (PBS) and stored at 4C. Data were collected on a three-laser BD LSRFortessa (Becton Dickinson) and analyzed with FlowJo software (FlowJo LLC, Ashland, OR). The mean fluorescence intensity (geometric) of PE and FITC was determined from 30,000 events after compensation correction. Data were normalized to vehicle (DMSO) controls.

Compounds were screened for inhibition activity against human PAR4 using 100 nM γ-thrombin or 200 μM PAR4-AP in a single-point assay (10 μM test compound). Dose–response curves were conducted for active compounds. Active compounds were also assessed at a single concentration of 10 μM against other agonists, including the PAR4 component of α-thrombin (5 nM α-thrombin +1 μM vorapaxar), the PAR1 agonist peptide (20 μM SFFLRN), and the GPVI receptor agonist convulxin (3 nM).

### Mouse Platelet Activity Assay

For mouse PAR4 activity, washed mouse platelets were prepared by collecting cardiac puncture blood with 200 μL of 20 U/mL heparin. Following centrifugation at 1000 rpm for 15 min, plasma and buffy coat were collected into a separate tube and spiked with apyrase and PGE_1_. After centrifuging at 1000 rpm for 8 min, the platelet-rich plasma was transferred to another tube and centrifuged at 2200 rpm for 10 min to pellet the platelets. Platelets were resuspended in TBB + apyrase + PGE1. Platelets were counted and diluted in TBB to a final concentration of 2 × 10^7^ platelets/mL. 50 μL of platelets were aliquoted to each tube preincubated with 50 μL of TBB with antibodies for mouse GPIIbIIIa and P-selectin (Emfret, Eibelstadt Germany) and either test compound antagonist or DMSO control. Platelets were activated with 316 nM γ-thrombin for 30 min. Samples were then fixed with 100 μL of 2% paraformaldehyde for 20 min and diluted with 300 μL of PBS. Flow cytometry counts are measured as for human platelets on a three-laser BD LSRFortessa (Becton Dickinson) and analyzed with FloJo software.

### Drug Metabolism and Pharmacokinetics

#### Materials

Potassium phosphate, ammonium formate, formic acid, magnesium chloride, and carbamazepine were purchased from Sigma-Aldrich (St. Louis, MO). Human liver microsomes (HLMs) and mouse liver microsomes (MLMs) were obtained from BD Biosciences (Billerica, MA), with pooled gender at a concentration of 20 mg/mL protein. Microsomes were stored in a −80 °C freezer. All solvents used for bioanalysis were purchased from Sigma-Aldrich or Fisher Scientific (Waltham, MA) and were of high-performance liquid chromatography (HPLC) grade.

#### Microsomal Stability

The metabolic stability of each compound was investigated in mouse and human hepatic microsomes using substrate depletion methodology (% parent compound remaining). Prior to use, microsomes were removed from the freezer and allowed to thaw in a 37 °C water bath before being placed on wet ice.

In separate 96-well plates for each time point, a mixture of 0.1 M potassium phosphate-buffered (pH 7.4), 3 mM MgCl_2_, and 2.5 mg/mL microsomes were prewarmed at 37 °C for 5 min. Following the preincubation, a 1 μM test compound and 1 mM NADPH (for 3, 7, 15, 25, or 45 min time points) or buffer (for 0 min time point) were added to initiate the reaction. Under standard conditions, reactions were initiated with the addition of NADPH. To delineate the contribution of esterases, some experiments were initiated by the addition of substrate in the presence or absence of NADPH. Plates were incubated at 37 °C under ambient oxygenation. At the respective times, each plate’s reaction was precipitated by the addition of 2 volumes of ice-cold acetonitrile containing an internal standard (carbamazepine, 50 nM). The plates were centrifuged at 4000 rcf (4 °C) for 5 min. The resulting supernatants were transferred and diluted 1:1 (supernatant:water) into new 96-well plates in preparation for LC/MS/MS analysis. Each compound was assayed in triplicate within the same 96-well plate. The in vitro half-life (*t*_1/2_, min, eq 1), intrinsic clearance (CL_int_, mL/min/kg, eq 2), and subsequent predicted hepatic clearance (CL_hep_, mL/min/kg, eq 3) were determined employing the following equations
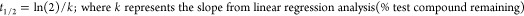
1
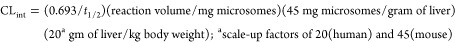
2
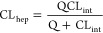
3The estimated percent contribution to metabolism was calculated from the percent substrate lost plots generated from metabolic incubations initiated with the addition of a test compound. Calculations compared the area under the curve (AUC) from conditions in the presence and absence of NADPH and buffer-only (control) conditions (eqs 4 and 5). AUC was calculated by using the trapezoidal method
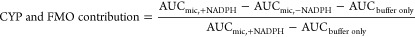
4
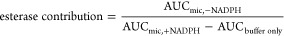
5

#### Plasma Protein Binding

The protein binding of each compound was determined in mouse plasma via equilibrium dialysis employing single-use RED plates with inserts (Thermo Fisher Scientific, Rochester, NY). Plasma (220 μL) was added to a 96-well plate containing test compound (5 μL) and mixed thoroughly. Subsequently, 200 μL of the plasma–compound mixture was transferred to the cis chamber (red) of the RED plate with an accompanying 350 μL of phosphate buffer (25 mM, pH 7.4) in the trans chamber. The RED plate was sealed and incubated for 4 h at 37 °C with shaking. At completion, 50 μL aliquots from each chamber were diluted 1:1 (50 μL) with either plasma (cis) or buffer (trans) and transferred to a new 96-well plate, at which time ice-cold acetonitrile (2 volumes) was added to extract the matrices. The plate was centrifuged (3000 rpm, 10 min), and supernatants were transferred and diluted 1:1 (supernatant: water) into a new 96-well plate, which was then sealed in preparation for LC/MS/MS analysis. Each compound was assayed in triplicate within the same 96-well plate.

#### Liquid Chromatography-Mass Spectrometry Analysis

The analysis of in vitro samples from microsomal stability or plasma protein binding experiments was determined by employing LCMS/MS with an electrospray ionization-enabled 5500 Sciex instrument (Sciex, Foster City, CA) that was coupled to Agilent HPLC pumps and an autosampler (Agilent Technologies, Santa Clara, CA). Analytes were separated by gradient elution using a Kinetex C18 column (2.1 mm × 50 mm, 5 μm; Phenomenex, Torrance, CA) warmed to 35 °C. Mobile phase A was 0.5% formic acid in water, and mobile phase B was 0.5% formic acid in acetonitrile. The gradient started at 5% B after a 0.2 min hold and was linearly increased to 95% B over 1.5 min, held at 95% B for 0.2 min, and returned to 5% B in 0.1 min, followed by a re-equilibration (0.3 min). The total run time was 2.8 min, and the HPLC flow rate was 0.5 mL/min. Mass spectral analyses were performed using multiple reaction monitoring, with transitions and voltages specific for each analyte using a Turbo Ion Spray source (source temperature of 500 °C) in positive ionization mode (5.0 kV spray voltage). Multiple reaction monitoring transitions were the following: 6110B8 (*m*/*z* 345.02 → 149.03, DP = 31; EP = 10; CE = 37 and CXP = 14), 6G3R (*m*/*z* 421.06 → 225.12, DP = 91; EP = 10; CE = 39 and CXP = 12), 5D22 (*m*/*z* 309.068 → 211.16, DP = 131; EP = 10; CE = 29 and CXP = 12), and carbamazepine (*m*/*z* 237 → 194, DP = 96; EP = 10; CE = 25 and CXP = 8). Data were analyzed using OS Sciex Analyst 1.5.1 (SCIEX, Framingham, MA), Graphpad Prism 10.1.0 (GraphPad Software, Boston, Massachusetts), and Microsoft Excel version 2310 (Microsoft, Redmond, WA).

## Supporting Information Available

Compound synthesis analytical data with NMR spectra and computational protocol captures (PDF)
