# Supplementary material for: Discovery of Protease-Activated Receptor 4 (PAR4)-Tethered Ligand Antagonists Using Ultralarge Virtual Screening
Source: ACS Pharmacol Transl Sci. 2024 Mar 21;7(4):1086–100. doi: 10.1021/acsptsci.3c00378 (PMC11020070; doi:10.1021/acsptsci.3c00378)
Supplement: Supplementary file 1 — pt3c00378_si_001.pdf [file pt3c00378_si_001.pdf]

Supporting Information

Discovery of protease-activated receptor 4 (PAR4) tethered ligand antagonists using ultra-large virtual screening

Shannon T. Smith[2], Jackson B. Cassada[1], Lukas Von Bredow[3,5], Kevin Erreger[1], Emma M. Webb[1], Trevor A. Trombley[3], Jacob J. Kalbfleisch[2,3], Brian J. Bender[4], Irene Zagol-Ikapitte[3], Valerie M. Kramlinger[1,3], Jacob L. Bouchard[3], Sidnee G. Mitchell[1], Maik Tretbar[5], Brian K. Shoichet[4], Craig W. Lindsley[1,2,3], Jens Meiler[1,2,5*], Heidi E. Hamm[1*]

[1]Department of Pharmacology, Vanderbilt University, Nashville, TN USA

[2]Department of Chemistry, Vanderbilt University, Nashville, TN USA

[3]Warren Center for Neuroscience Drug Discovery, Nashville, TN USA

[4]Department of Pharmaceutical Chemistry, University of California San Francisco, San Francisco, CA USA

[5]Institute for Drug Discovery, Leipzig University Medical School, Leipzig, Germany

[*]Correspondence: jens@meilerlab.org, heidi.hamm@vanderbilt.edu

Table of Contents

1.1 Analytical Data of Final Compounds .... S 3

1.2 ^{1}H and ^{13}C NMR Spectra .... S 10

1.3 Protocol Captures.......... S 38

1.1 Analytical Data of Final Compounds

1.1.1 N-[3-[3-(difluoromethoxy)phenyl]-1H-1,2,4-triazol-5-yl]-1-(3-fluorophenyl)-5,6-dihydro-4H-cyclopenta[d]pyrazole-3-carboxamide (18)

Synthesis according to the general procedure using 1-(3-fluorophenyl)-5,6-dihydro-4H-cyclopenta[d]pyrazole-3-carboxylic acid and 3-[3-(difluoromethoxy)phenyl]-1H-1,2,4-triazol-5-amine yielded 38.6 mg (62 %) as a white solid.

^{1}H NMR (400 MHz, DMSO): δ= 7.91 – 7.87 (m, 2H), 7.86 (t, J = 1.2 Hz, 1H), 7.74 (t, J = 1.9 Hz, 1H), 7.66 – 7.60 (m, 3H), 7.58 (t, J = 8.0 Hz, 1H), 7.33 (t, J = 73.8 Hz, 1H), 7.32 – 7.26 (m, 2H), 3.17 (t, ^{3}J = 7.3 Hz, 2H), 2.92 (t, ^{3}J = 7.2 Hz, 2H), 2.63 – 2.52 (m, 2H).
^{13}C NMR (101 MHz, DMSO): δ= 162.92 (d, J = 245.8 Hz), 161.74, 159.37, 159.09, 151.65 (d, J = 3.2 Hz), 150.88, 141.02 (d, J = 10.4 Hz), 139.42, 132.69, 132.55, 132.16 (d, J = 9.2 Hz), 131.24, 123.46, 121.07, 116.79 (t, J = 258.3 Hz), 116.55, 116.30 (d, J = 2.5 Hz), 114.62 (d, J = 20.7 Hz), 107.69 (d, J = 26.5 Hz), 30.36, 26.24 (2C).
HRMS (ESI) *m/z* calculated for $[M+H]^{+}$= 455.1438, found: 455.1443.

1.1.2 1-(3-fluorophenyl)-N-(3-methyl-1H-1,2,4-triazol-5-yl)-5,6-dihydro-4H-cyclopenta[d]pyrazole-3-carboxamide (19)

Synthesis according to the general procedure using 1-(3-fluorophenyl)-5,6-dihydro-4H-cyclopenta[c]pyrazole-3-carboxylic acid and 3-amino-5-methyl-4H-1,2,4-triazole yielded 73.8 mg (30 %) as a white solid.

^{1}H NMR (400 MHz, DMSO): δ= 7.70 (td, ^{3}J = 7.9 Hz, ^{4}J = 1.6 Hz, 1H), 7.61 – 7.47 (m, 4H), 7.40 (ddd, ^{3}J = 7.6, 6.8Hz, ^{4}J = 1.7 Hz, 1H), 2.85 (t, ^{3}J = 7.2 Hz, 2H), 2.80 (t, ^{3}J = 7.3 Hz, 2H), 2.58 – 2.44 (m, 2H), 2.12 (s, 3H) ppm.
^{13}C NMR (101 MHz, DMSO): δ= 161.25, 160.11, 158.08, 154.60 (d, J = 249.9 Hz), 152.35, 139.75, 130.75, 130.61 (d, J = 7.8 Hz), 126.91, 126.89 (d, J = 11.1 Hz), 125.47 (d, J = 3.9 Hz), 117.05 (d, J = 19.5 Hz), 29.66, 26.10, 24.27 (d, J = 4.9 Hz), 13.99 ppm.
HRMS (ESI) *m/z* calculated for $[M+H]^{+}$= 327.1364, found: 327.1367.

1.1.3 N-[3-[3-(difluoromethoxy)phenyl]-1H-1,2,4-triazol-5-yl]-1-(4-fluorophenyl)-5,6-dihydro-4H-cyclopenta[d]pyrazole-3-carboxamide (20)

Synthesis according to the general procedure using 1-(4-fluorophenyl)-5,6-dihydro-4H-cyclopenta[c]pyrazole-3-carboxylic acid and 3-[3-(difluoromethoxy)phenyl]-1H-1,2,4-triazol-5-amine yielded 5.4 mg (20 %) as a white solid.

^{1}H NMR (400 MHz, DMSO): δ= 7.88 – 7.83 (m, 2H), 7.83 – 7.79 (m, 2H), 7.74 (t, *J* = 2.0 Hz, 1H), 7.57 (t, *J* = 8.0 Hz, 1H), 7.45 – 7.38 (m, 2H), 7.32 (t, ^{2}J = 73.7 Hz, 1H), 7.31 – 7.27 (m, 1H), 3.11 (t, ^{3}J = 7.3 Hz, 2H), 2.92 (t, ^{3}J = 7.2 Hz, 2H), 2.56 (p, ^{3}J = 7.6 Hz, 2H) ppm.
^{13}C NMR (101 MHz, DMSO): δ= 161.82, 161.33 (d, *J* = 244.6 Hz), 159.29, 159.07, 151.62 (t, *J* = 3.2 Hz), 150.63, 138.93, 136.20 (d, *J* = 2.7 Hz), 132.57, 132.43, 131.23, 123.45, 122.66 (d, *J* = 8.7 Hz), 121.04, 117.01 (d, *J* = 23.0 Hz), 116.80 (t, *J* = 258.3 Hz), 116.56, 30.43, 26.47, 26.02 ppm.

HRMS (ESI) *m/z* calculated for $[M+H]^+$= 455.1438, found: 455.1439.

1.1.4 1-(4-fluorophenyl)-N-(3-methyl-1H-1,2,4-triazol-5-yl)-5,6-dihydro-4H-cyclopenta[d]pyrazole-3-carboxamide (21)

Synthesis according to the general procedure using 1-(4-fluorophenyl)-5,6-dihydro-4H-cyclopenta[c]pyrazole-3-carboxylic acid and 3-amino-5-methyl-4H-1,2,4-triazole yielded 15.3 mg (68 %) as a white solid.

^{1}H NMR (400 MHz, DMSO): δ= 7.82 – 7.71 (m, 2H), 7.57 (bs, 1H), 7.45 – 7.34 (m, 2H), 3.06 (t, ^{3}J = 7.3 Hz, 2H), 2.82 (t, ^{3}J = 7.2 Hz, 2H), 2.58 – 2.51 (m, 2H), 2.11 (s, 3H) ppm.
^{13}C NMR (101 MHz, DMSO): δ= 161.30, 160.79 (d, *J* = 244.5 Hz), 160.00, 158.05, 149.87, 138.87, 135.75 (d, *J* = 2.8 Hz), 131.72, 122.13 (d, *J* = 8.6 Hz), 116.50 (d, *J* = 23.1 Hz), 29.74, 25.75, 25.49, 13.97 ppm.

HRMS (ESI) *m/z* calculated for $[M+H]^+$= 327.1364, found: 327.1367.

1.1.5 1-(2-fluorophenyl)-N-(3-methyl-1H-1,2,4-triazol-5-yl)-5,6-dihydro-4H-cyclopenta[d]pyrazole-3-carboxamide (22)

Synthesis according to the general procedure using 1-(2-fluorophenyl)-5,6-dihydro-4H-cyclopenta[c]pyrazole-3-carboxylic acid and 3-methyl-1H-1,2,4-triazol-5-amine yielded 16.2 mg (85 %) as a white solid.

^{1}H NMR (400 MHz, DMSO): δ= 7.70 (td, ^{3}J = 7.9 Hz, ^{4}J = 1.7 Hz, 1H), 7.61 – 7.47 (m, 4H), 7.44 – 7.37 (m, 1H), 2.83 (dt, J = 18.1, 7.2 Hz, 4H), 2.55 – 2.51 (m, 2H), 2.12 (s, 3H).
^{13}C NMR (101 MHz, DMSO): δ= 161.25, 160.10, 158.08, 154.60 (d, J = 250.0 Hz), 152.34, 139.75, 130.75, 130.61 (d, J = 7.9 Hz), 126.91, 126.89 (d, J = 11.1 Hz), 125.47 (d, J = 3.7 Hz), 117.05 (d, J = 19.4 Hz), 29.66, 26.10, 24.27 (d, J = 4.9 Hz), 13.98 ppm.
HRMS (ESI) *m/z* calculated for $[M+H]^{+}$= 327.1364, found: 327.1369.

1.1.6 N-[3-[3-(difluoromethoxy)phenyl]-1H-1,2,4-triazol-5-yl]-1-(2-fluorophenyl)-5,6-dihydro-4H-cyclopenta[d]pyrazole-3-carboxamide (23)

Synthesis according to the general procedure using 1-(2-fluorophenyl)-5,6-dihydro-4H-cyclopenta[d]pyrazole-3-carboxylic acid and 3-[3-(difluoromethoxy)phenyl]-1H-1,2,4-triazol-5-amine yielded 62.2 mg (35 %) as a white solid.

^{1}H NMR (400 MHz, DMSO): δ= 7.90 – 7.82 (m, 3H), 7.74 (t, ^{4}J = 1.9 Hz, 1H), 7.65 – 7.59 (m, 3H), 7.57 (t, ^{3}J = 8.0 Hz, 1H), 7.32 (t, ^{2}J = 73.8 Hz, 1H), 7.32 – 7.24 (m, 2H), 3.16 (t, ^{3}J = 7.2 Hz, 2H), 2.91 (t, ^{3}J = 7.2 Hz, 2H), 2.62 – 2.51 (m, 2H) ppm.
^{13}C NMR (101 MHz, DMSO): δ= 162.45 (d, J = 244.8 Hz), 161.27, 158.90, 158.62, 151.16 (t, J = 3.2 Hz), 150.41, 140.55 (d, J = 10.3 Hz), 138.95, 132.23, 132.08, 131.69 (d, J = 9.2 Hz), 130.77, 122.99, 120.60, 116.33 (t, J = 258.3 Hz), 116.10, 115.83 (d, J = 3.0 Hz), 114.14 (d, J = 21.2 Hz), 107.22 (d, J = 26.1 Hz), 29.90, 25.78 ppm.

HRMS (ESI) *m/z* calculated for $[M+H]^{+}$= 455.1438, found: 455.1440.

1.1.7 N-(3-cyclopropyl-1H-1,2,4-triazol-5-yl)-1-phenyl-5,6-dihydro-4H-cyclopenta[d]pyrazole-3-carboxamide (24)

Synthesis according to the general procedure using 1-phenyl-1H,4H,5H,6H-cyclopenta[C]pyrazole-3-carboxylic acid and 3-cyclopropyl-1H-1,2,4-triazol-5-amine yielded 9.4 mg (6 %) as a white solid.

^{1}H NMR (400 MHz, DMSO): δ= 7.77 – 7.71 (m, 2H), 7.61 – 7.53 (m, 3H), 7.45 – 7.38 (m, 1H), 3.10 (t, ^{3}J = 7.3 Hz, 2H), 2.86 (t, ^{3}J = 7.2 Hz, 2H), 2.54 (q, ^{3}J = 7.2 Hz, 2H), 1.84 (tt, ^{3}J = 8.2, 4.9 Hz, 1H), 0.94 – 0.79 (m, 4H) ppm.
^{13}C NMR (101 MHz, DMSO): δ= 165.18, 161.60, 158.53, 150.25, 139.71, 139.28, 132.17, 130.18 (2 C), 127.73, 120.33 (2 C), 30.23, 26.25, 26.19, 9.35, 7.44 (2 C) ppm.
HRMS (ESI) *m/z* calculated for $[M+H]^+$= 335.1615, found: 335.1617.

1.1.8 N-(5-cyclopropyl-1H-1,2,4-triazol-3-yl)-1-phenyl-4,5,6,7-tetrahydro-1H-indazole-3-carboxamide, (26)

50 mg (0.21 mmol, 1.0 eq.)1-Phenyl-4,5,6,7-tetrahydro-1*H*-indazole-3-carboxylic acid, 59 mg (0.31 mmol, 1.5 eq.) 1-Ethyl-3-(3-dimethylaminopropyl)carbodiimide hydrochloride, 43 µL (0.31 mmol, 1.5 eq.) trimethylamine, 38 mg (0.31 mmol, 1.5 eq.) diemethylaminopyridine and 38 mg (0.31 mmol, 1.5 eq.) 5-Cyclopropyl-1*H*-1,2,4-triazol-3-amine in 1 mL acetonitrile were used. The reaction yielded 45 mg (61 %) as a white solid.

^{1}H NMR (400 MHz, CD_3OD+ $CDCl_3$): δ= 7.41 – 7.34 (m, 2H), 7.33 – 7.27 (m, 3H), 2.76 (t, ^{3}J = 6.3 Hz, 2H), 2.66 (t, ^{3}J = 6.2 Hz, 2H), 1.94 – 1.84 (m, 2H), 1.84 – 1.74 (m, 2H), 1.64 (tt, ^{3}J = 8.3, 4.9 Hz, 1H), 0.90 – 0.77 (m, 2H), 0.78 – 0.68 (m, 2H) ppm.
^{13}C-NMR (101 MHz, CD_3OD+ $CDCl_3$): δ=166.58, 161.45, 158.76, 151.35, 141.14, 132.03, 129.99, 128.61, 124.38, 123.84, 23.77, 23.64, 23.50, 22.03, 9.27, 7.67 ppm.
HRMS (ESI) *m/z* calculated for $[M+H]^+$= 349.1771, found: 349.1766.

1.1.9 1-Phenyl-N-(5-(3-(trifluoromethyl)phenyl)-1H-1,2,4-triazol-3-yl)-4,5,6,7-tetrahydro-1H-indazole-3-carboxamide (27)

Synthesis according to the general procedure using 1-Phenyl-4,5,6,7-tetrahydro-1*H*-indazole-3-carboxylic acid and 5-(3-(trifluoromethyl)phenyl)-1*H*-1,2,4-triazol-3-amine yielded 30 mg (32 %) as a white solid.

^{1}H NMR (400 MHz, DMSO): δ= 8.03 (dd, ^{3}J = 8.0; ^{4}J = 1.7 Hz, 1H), 7.95 (d, ^{4}J = 1.9 Hz, 1H), 7.89 (bs, 1H), 7.84 – 7.77 (m, 1H), 7.67 (t, ^{3}J = 7.9 Hz, 1H), 7.38 – 7.33 (m, 4H), 7.21 (tt, ^{3}J = 6.3; ^{4}J = 2.3 Hz, 1H), 2.74 (t, ^{3}J = 6.0 Hz, 2H), 2.67 (t, ^{3}J = 6.2 Hz, 2H), 1.85 – 1.79 (m, 2H), 1.77 – 1.69 (m, 2H).
^{13}C NMR (101 MHz, DMSO): δ= 159.95, 158.61, 158.21, 149.73, 140.35, 130.67, 130.29, 130.09, 130.05, 129.34 (q, J = 32.0 Hz), 127.27, 126.65, 123.94 (q, J = 272.2 Hz), 123.26, 122.83, 122.58 (q, J = 1.9 Hz), 122.51 (q, J = 3.9 Hz), 22.70, 22.46, 22.27, 20.98 ppm.
HRMS (ESI) *m/z* calculated for $[M+H]^+$= 453.1645, found: 453.1636.

1.1.10 N-(5-cyclopropyl-1H-1,2,4-triazol-3-yl)-1-(3-fluorophenyl)-4,5,6,7-tetrahydro-1H-indazole-3-carboxamide (28)

Synthesis according to the general procedure using 1-(3-Fluorophenyl)-4,5,6,7-tetrahydro-1*H*-indazole-3-carboxylic acid and 5-Cyclopropyl-1*H*-1,2,4-triazol-3-amine yielded 34 mg (44 %) as a white solid.

^{1}H NMR (400 MHz, DMSO): δ= 7.66 – 7.45 (m, 5H), 7.36 – 7.27 (m, 1H), 2.81 (t, ^{3}J = 5.9 Hz, 2H), 2.62 (t, ^{3}J = 5.9 Hz, 1H), 1.84 – 1.66 (m, 5H), 0.91 – 0.72 (m, 4H) ppm.
^{13}C NMR (101 MHz, DMSO): δ= 164.85, 162.54, 162.13 (d, J = 244.9 Hz), 158.04, 142.92, 140.26 (d, J = 10.3 Hz), 139.84, 131.21 (d, J = 9.1 Hz), 119.96, 119.42 (d, J = 2.9 Hz), 114.67 (d, J = 21.2 Hz), 110.70 (d, J = 25.3 Hz), 22.66, 22.15, 21.85, 21.16, 8.83, 6.87 ppm.
HRMS (ESI) *m/z* calculated for $[M+H]^+$= 367.1677, found: 367.1677.

1.1.11 1-Phenyl-N-(5-phenyl-1H-1,2,4-triazol-3-yl)-4,5,6,7-tetrahydro-1H-indazole-3-carboxamide (29)

Synthesis according to the general procedure using 1-Phenyl-4,5,6,7-tetrahydro-1*H*-indazole-3-carboxylic acid and 5-(3-Phenyl)-1*H*-1,2,4-triazol-3-amine yielded 17 mg (21 %) as a white solid.

^{1}H NMR (400 MHz, DMSO): δ= 7.85 – 7.74 (m, 3H), 7.46 – 7.33 (m, 6H), 7.28-7.21 (m, 1H), 2.74 (t, ^{3}J = 6.3 Hz, 2H), 2.65 (t, ^{3}J = 6.2 Hz, 2H), 1.83 (qd, ^{3}J = 7.7, 6.6, 3.8 Hz, 2H), 1.71 (dd, ^{3}J = 7.1, 4.2 Hz, 2H) ppm.
^{13}C NMR (101 MHz, DMSO): δ= 160.14, 160.12, 158.05, 149.62, 140.14, 130.60, 130.17, 129.74, 129.15, 128.61, 127.32, 126.39, 123.01, 122.72, 22.73, 22.47, 22.30, 21.06 ppm.
HRMS (ESI) *m/z* calculated for $[M+H]^{+}$= 385.1771, found: 385.1766.

1.1.12 N-(5-(4-cyanophenyl)-1H-1,2,4-triazol-3-yl)-1-phenyl-4,5,6,7-tetrahydro-1H-indazole-3-carboxamide (30)

Synthesis according to the general procedure using 1-Phenyl-4,5,6,7-tetrahydro-1*H*-indazole-3-carboxylic acid and 5-(3-Phenyl)-1*H*-1,2,4-triazol-3-amine yielded 5 mg (5 %) as a white solid.

^{1}H NMR (400 MHz, DMSO): δ= 8.18 – 8.11 (m, 2H), 8.04 – 7.97 (m, 2H), 7.93 (bs, 2H), 7.76 – 7.68 (m, 2H), 7.64 (dd, *J* = 8.6, 7.0 Hz, 2H), 7.57 – 7.51 (m, 1H), 2.87 (t, ^{3}J = 6.0 Hz, 2H), 2.75 (t, ^{3}J = 6.0 Hz, 2H), 1.86 (d, ^{3}J = 6.4 Hz, 2H), 1.80 (d, ^{3}J = 6.8 Hz, 2H) ppm.
^{13}C NMR (101 MHz, DMSO): δ= 162.90, 158.72, 158.35, 141.93, 139.88, 138.86, 134.51, 132.88, 129.46, 128.06, 126.91, 123.55, 120.49, 118.60, 112.25, 22.72, 22.19, 21.97, 21.45 ppm.
HRMS (ESI) *m/z* calculated for $[M+H]^{+}$= 410.1724, found: 410.1715.

1.1.13 N-(5-(2,4-difluorophenyl)-1H-1,2,4-triazol-3-yl)-1-phenyl-4,5,6,7-tetrahydro-1H-indazole-3-carboxamide (31)

Synthesis according to the general procedure using 1-Phenyl-4,5,6,7-tetrahydro-1*H*-indazole-3-carboxylic acid and 5-(2,4-Difluorophenyl)-1*H*-1,2,4-triazol-3-amine yielded 49 mg (55 %) as a white solid.

^{1}H NMR (400 MHz, $CDCl_3$): δ= 8.06 (td, ^3J = 8.8, 6.6 Hz, 1H), 7.64 – 7.57 (m, 2H), 7.50 (dd, ^3J = 8.6, 7.0 Hz, 2H), 7.44 – 7.36 (m, 2H), 7.00 – 6.83 (m, 2H), 6.75 (bs, 2H), 2.87 (t, ^3J = 5.5 Hz, 2H), 2.77 (t, ^3J = 5.4 Hz, 2H), 1.91 – 1.78 (m, 4H) ppm.
^{13}C NMR (101 MHz, $CDCl_3$): δ= 163.90 (dd, *J* = 256.7, 11.8 Hz), 163.86, 161.36 (d, *J* = 251.1, 11.7 Hz), 158.47, 156.88 (d, *J* = 5.3 Hz), 141.67, 140.16, 139.43, 131.72 (dd, *J* = 10.0, 4.1 Hz), 129.32, 128.11, 123.94, 122.43, 115.06 – 114.84 (m), 111.71 (dd, *J* = 21.4, 3.6 Hz), 105.03 (t, *J* = 25.5 Hz), 23.75, 22.75, 22.57, 21.96 ppm.
HRMS (ESI) *m/z* calculated for $[M+H]^+$= 421.1583, found: 421.1576.

1.1.14 N-(5-(3-methoxyphenyl)-1H-1,2,4-triazol-3-yl)-1-phenyl-4,5,6,7-tetrahydro-1H-indazole-3-carboxamide (32)

Synthesis according to the general procedure using 1-Phenyl-4,5,6,7-tetrahydro-1*H*-indazole-3-carboxylic acid and 5-(3-Methoxyphenyl)-1H-1,2,4-triazol-3-amine yielded 32 mg (37 %) as a white solid.
^{1}H NMR (400 MHz, $CDCl_3$): δ= 7.68 – 7.66 (m, 4H), 7.64 – 7.57 (m, 2H), 7.54 – 7.45 (m, 1H), 7.45 – 7.29 (m, 1H), 7.05 – 6.95 (m, 1H), 6.57 (bs, 2H), 3.87 (2, 3H), 2.85 (d, ^3J = 5.6 Hz, 2H), 2.78 (d, ^3J = 6.1 Hz, 2H), 1.90 – 1.80 (m, 4H) ppm.
^{13}C NMR (101 MHz, $CDCl_3$): δ= 163.76, 159.83, 158.24, 146.22, 141.82, 140.24, 139.45, 131.11, 129.76, 129.34, 128.22, 124.01, 122.27, 119.67, 117.15, 111.50, 55.46, 23.77, 22.79, 22.65, 22.29 ppm.
HRMS (ESI) *m/z* calculated for $[M+H]^+$= 414.1925, found: 414.1919.

1.2 1H and 13C NMR Spectra

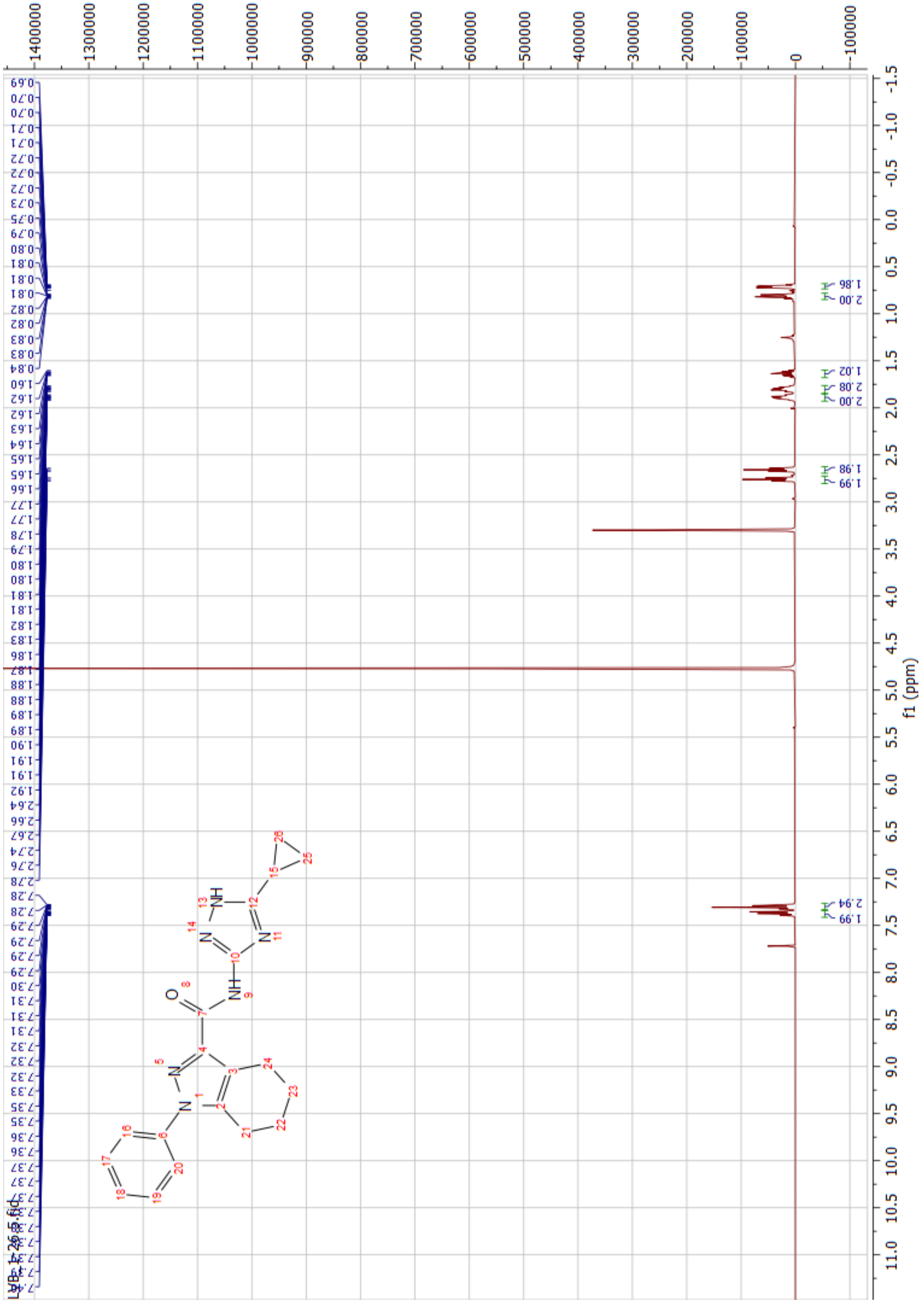

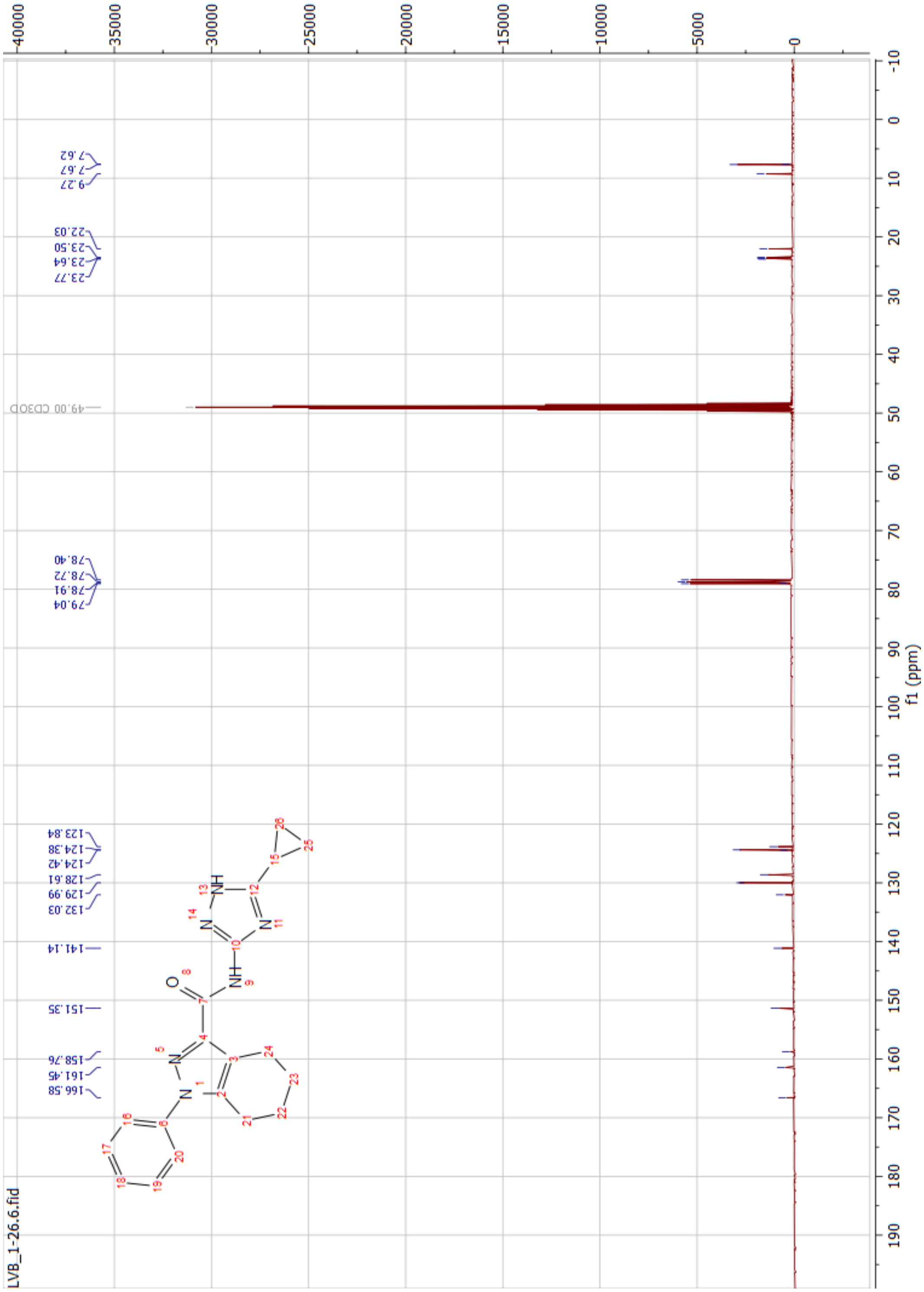

Figure S 1: ^{1}H (400 MHz) and ^{13}C NMR (101 MHz) NMR (DMSO) of N-(5-cyclopropyl-1H-1,2,4-triazol-3-yl)-1-phenyl-4,5,6,7-tetrahydro-1H-indazole-3-carboxamide, (**26**).

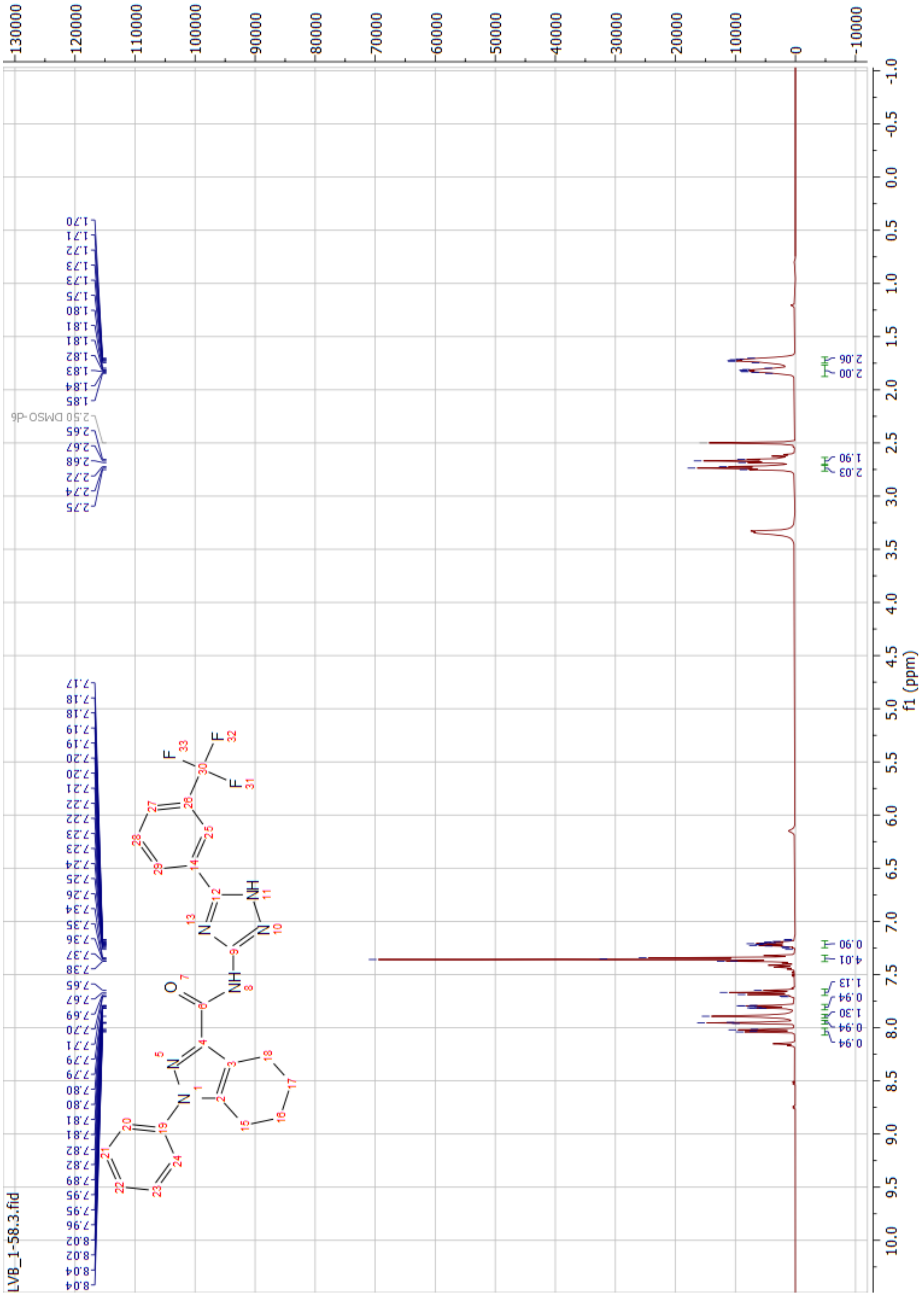

LVB_1-58.3.fid
f1 (ppm)
2.50 DMSO-d6

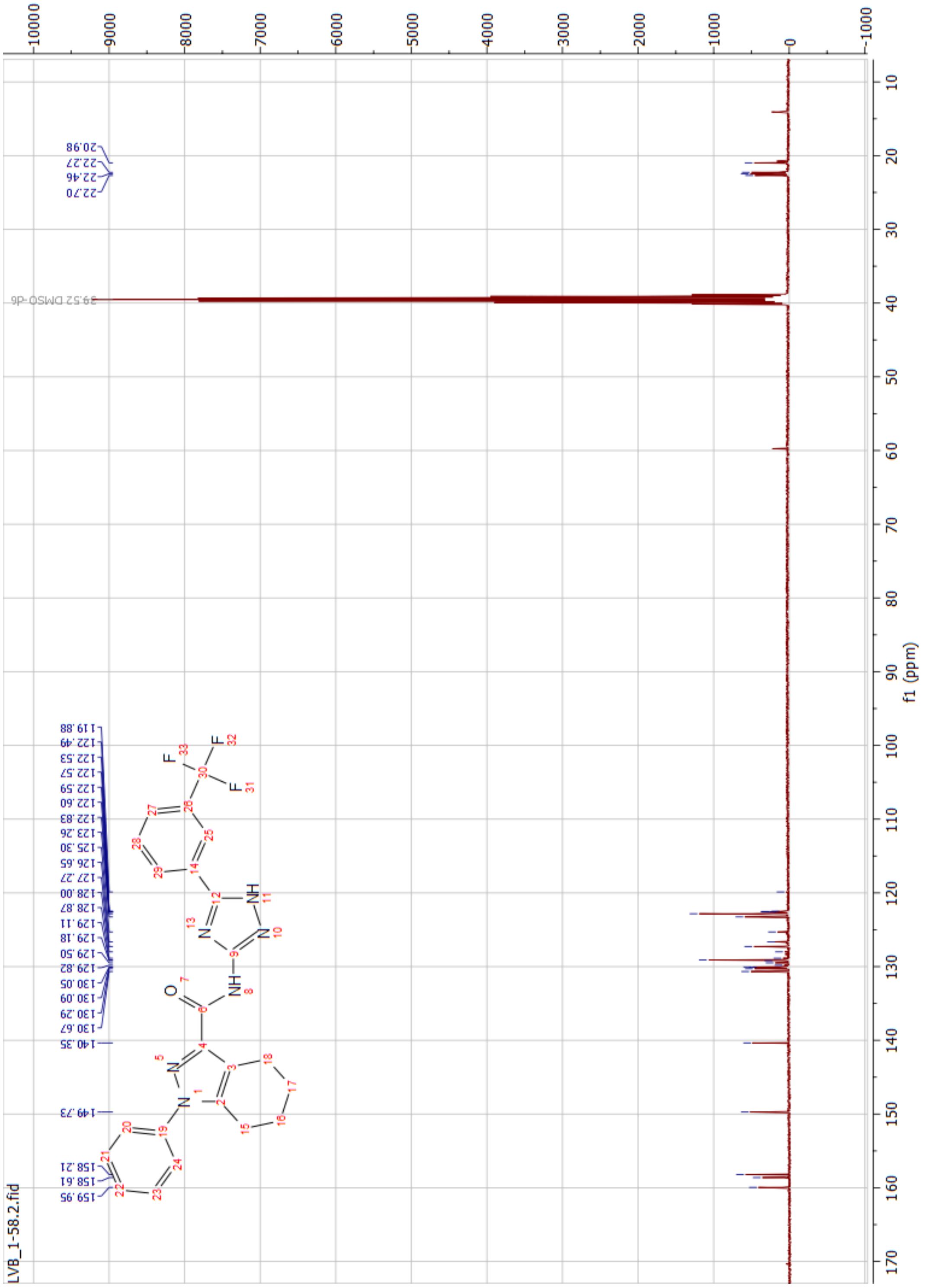

Figure S 2: ^{1}H (400 MHz) and ^{13}C NMR (101 MHz) NMR (DMSO) of 1-Phenyl-N-(5-(3 (trifluoromethyl)phenyl)-1H-1,2,4-triazol-3-yl)-4,5,6,7-tetrahydro-1H-indazole-3-carboxamid (**27**).

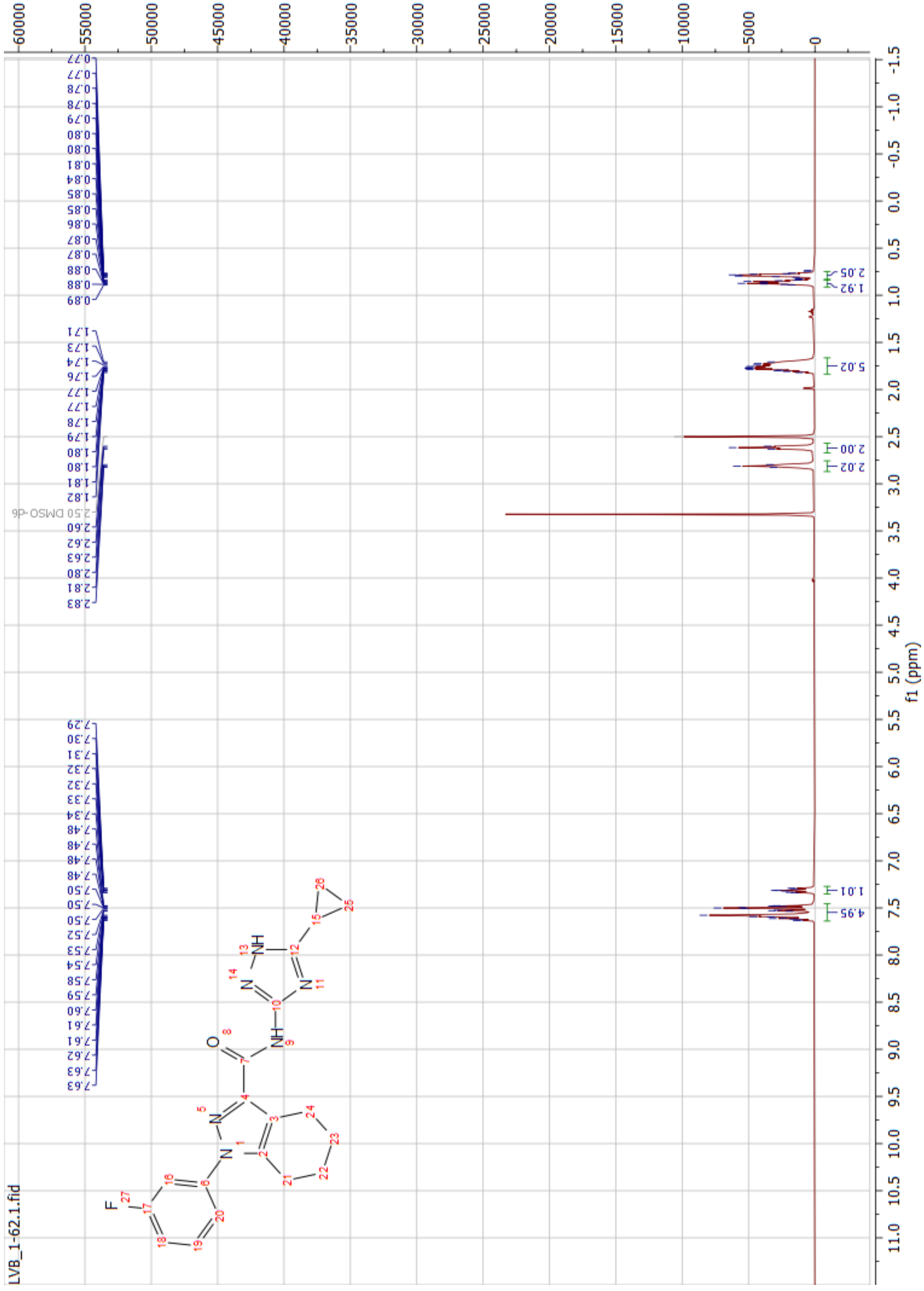
LVB_1-62.1.fid
f1 (ppm)
2.50 DMSO-d6

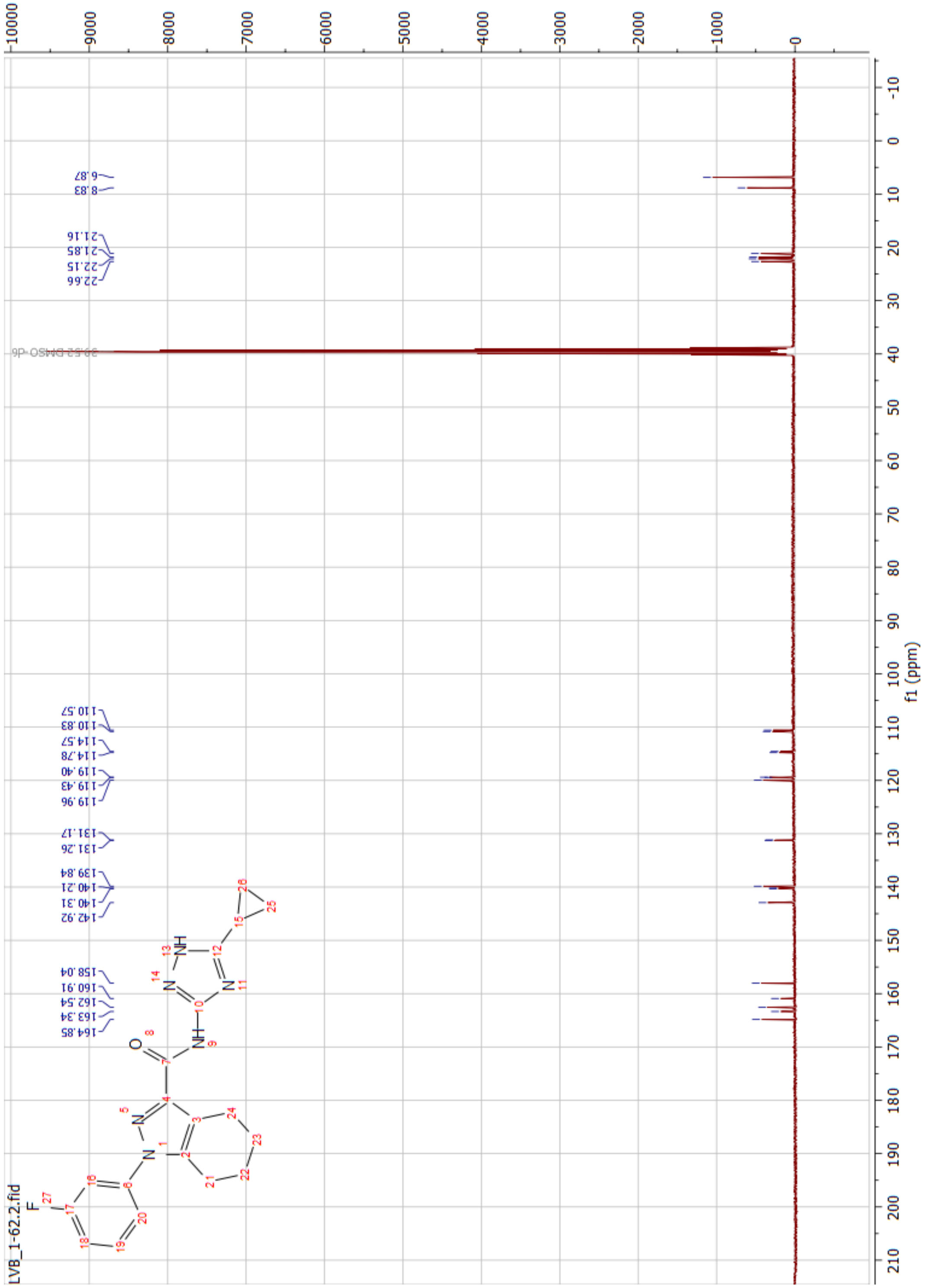

Figure S 3: ^{1}H (400 MHz) and ^{13}C NMR (101 MHz) NMR (DMSO) of N-(5-cyclopropyl-1H-1,2,4-triazol-3-yl)-1-(3-fluorophenyl)-4,5,6,7-tetrahydro-1H-indazole-3-carboxamide (**28**).

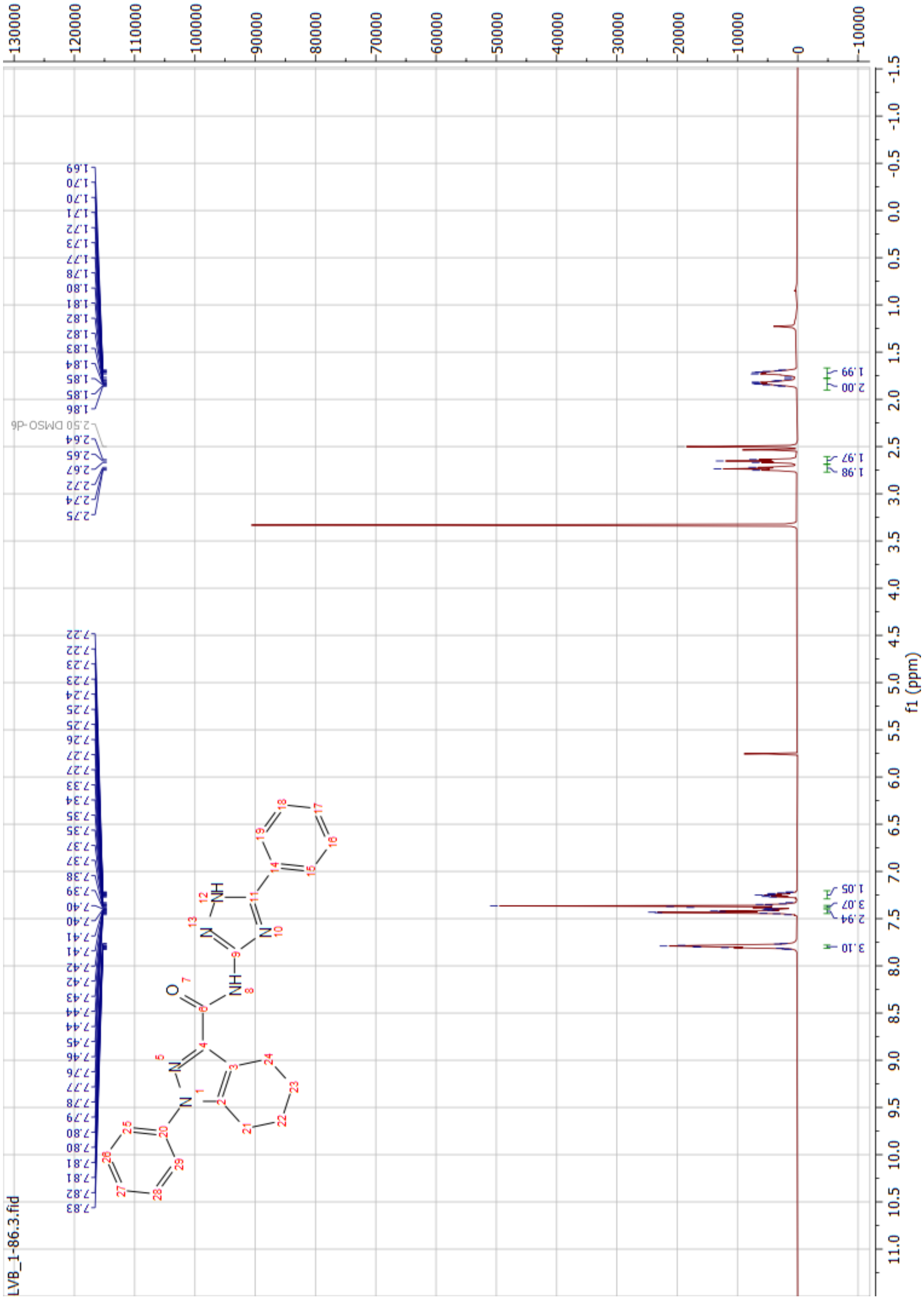
LVB_1-86.3.fid
f1 (ppm)
1.69
1.70
1.70
1.71
1.72
1.73
1.77
1.78
1.80
1.81
1.82
1.82
1.83
1.84
1.85
1.85
1.86
2.50 DMSO-d6
2.64
2.65
2.67
2.72
2.74
2.75
7.22
7.22
7.23
7.23
7.24
7.25
7.25
7.26
7.26
7.27
7.27
7.33
7.34
7.35
7.35
7.37
7.37
7.38
7.39
7.40
7.40
7.41
7.41
7.42
7.42
7.43
7.44
7.44
7.45
7.46
7.76
7.77
7.78
7.79
7.80
7.80
7.81
7.81
7.82
7.83
1.99
2.00
1.97
1.98
1.05
3.07
2.94
3.10

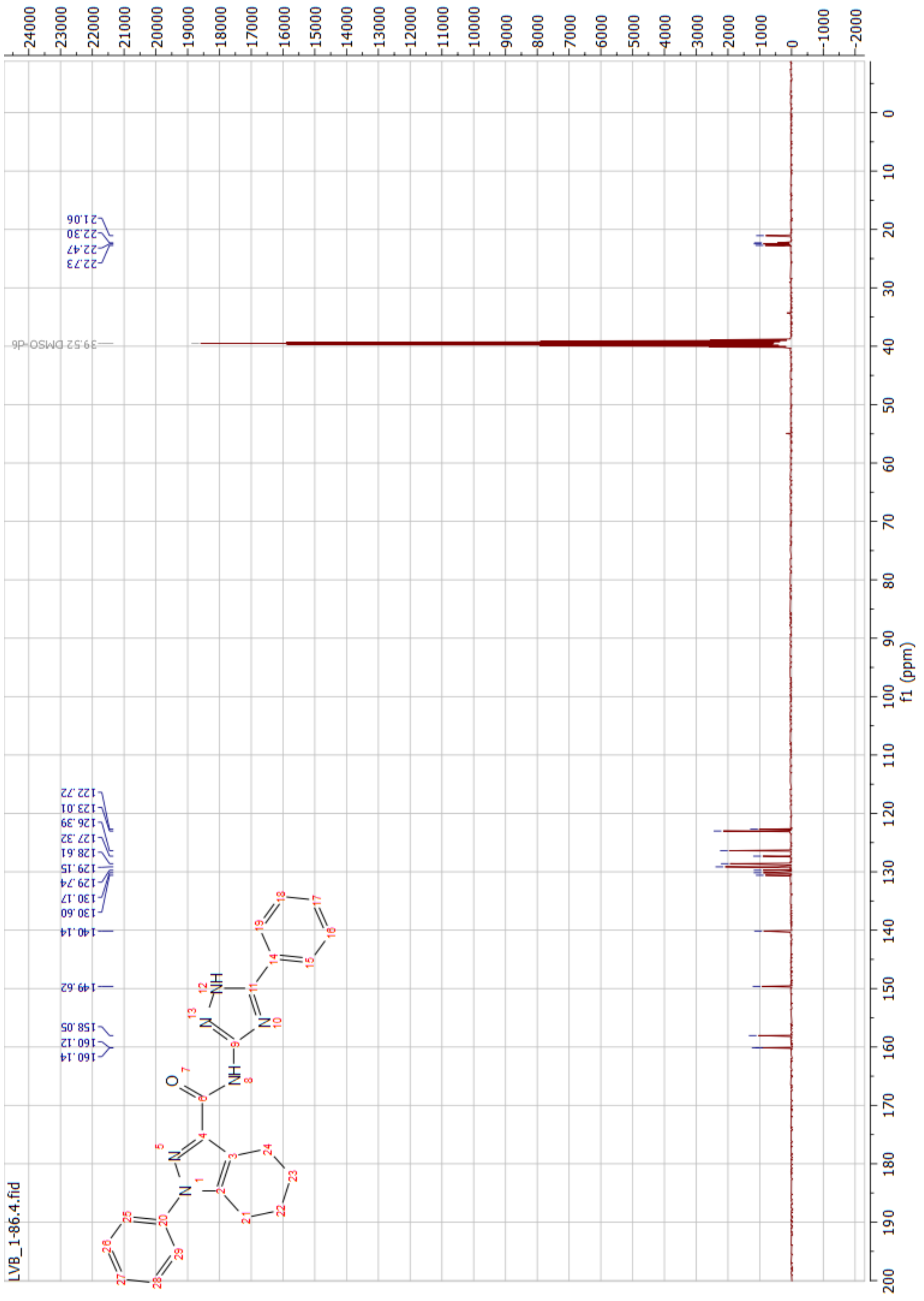

Figure S 4: ^{1}H (400 MHz) and ^{13}C NMR (101 MHz) NMR (DMSO) of 1-Phenyl-N-(5-phenyl-1H-1,2,4-triazol-3-yl)-4,5,6,7-tetrahydro-1H-indazole-3-carboxamide (**29**).

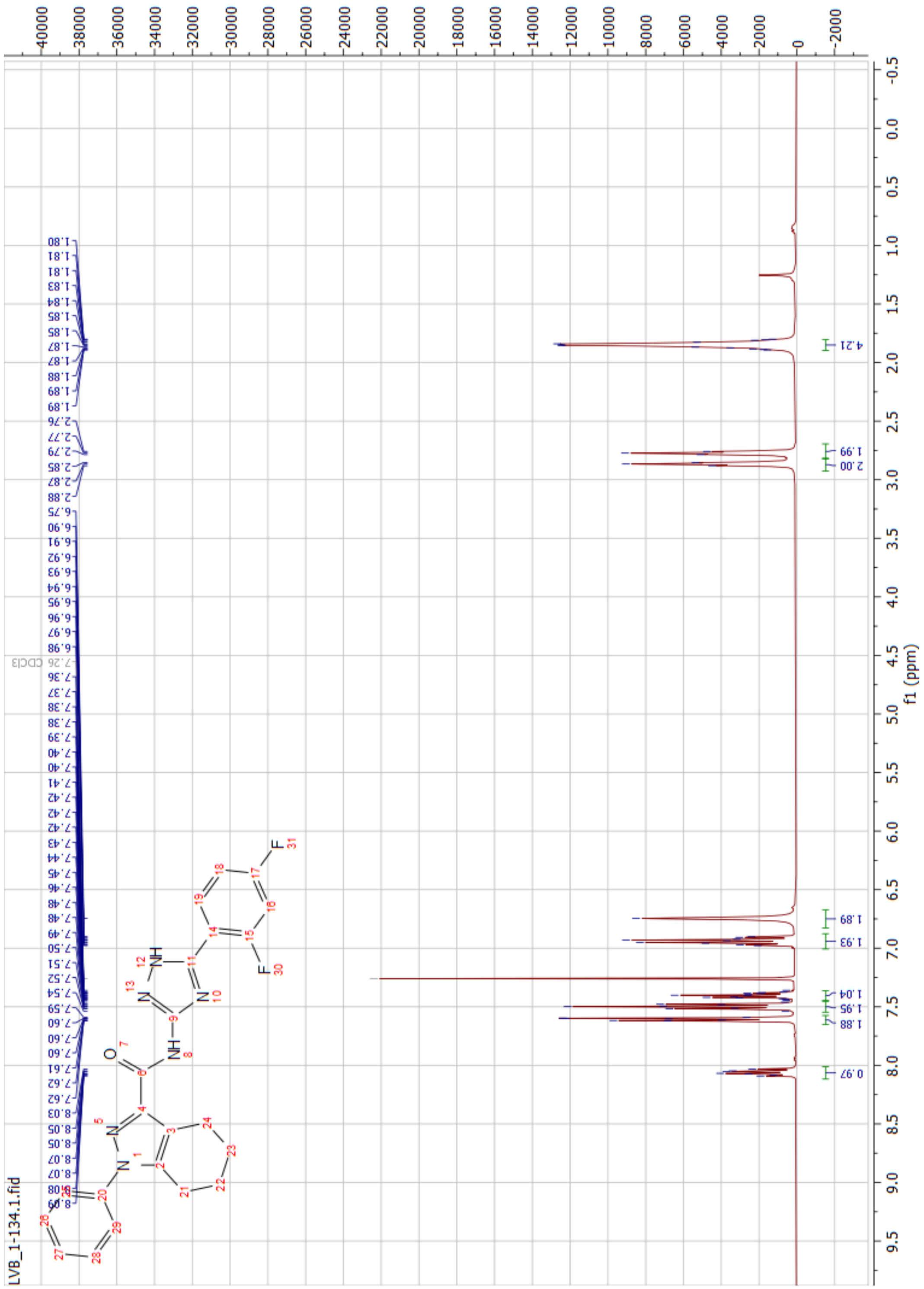

LVB_1-134.1.fid
f1 (ppm)

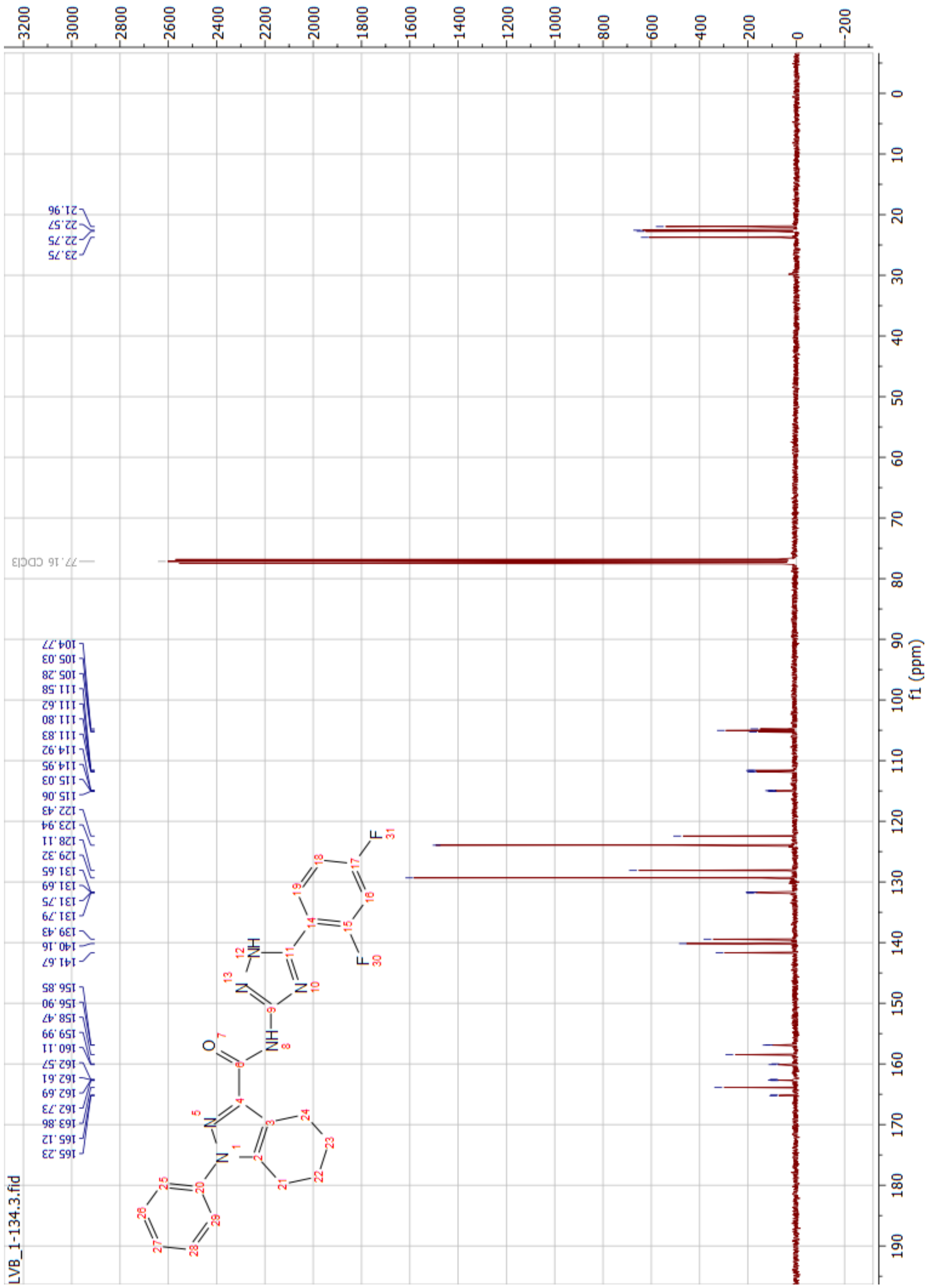

Figure S 5: ^{1}H (400 MHz) and ^{13}C NMR (101 MHz) NMR (DMSO) of N-(5-(2,4-difluorophenyl)-1H-1,2,4-triazol-3-yl)-1-phenyl-4,5,6,7-tetrahydro-1H-indazole-3-carboxamide (**31**).

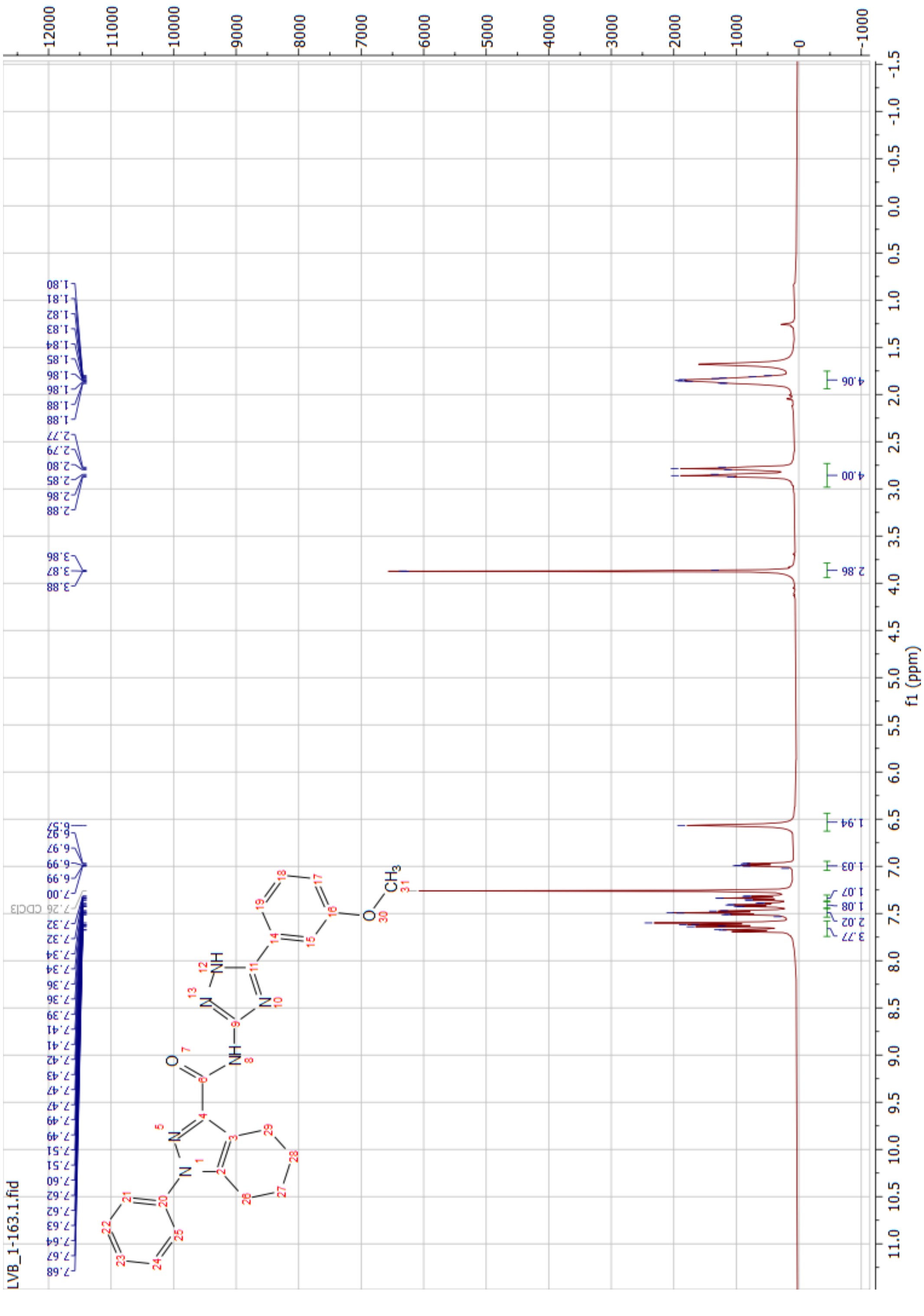

LVB_1-163.1.fid
f1 (ppm)
7.26 CDCl3
4.06
4.00
2.86
1.94
1.03
1.07
1.08
2.02
3.77

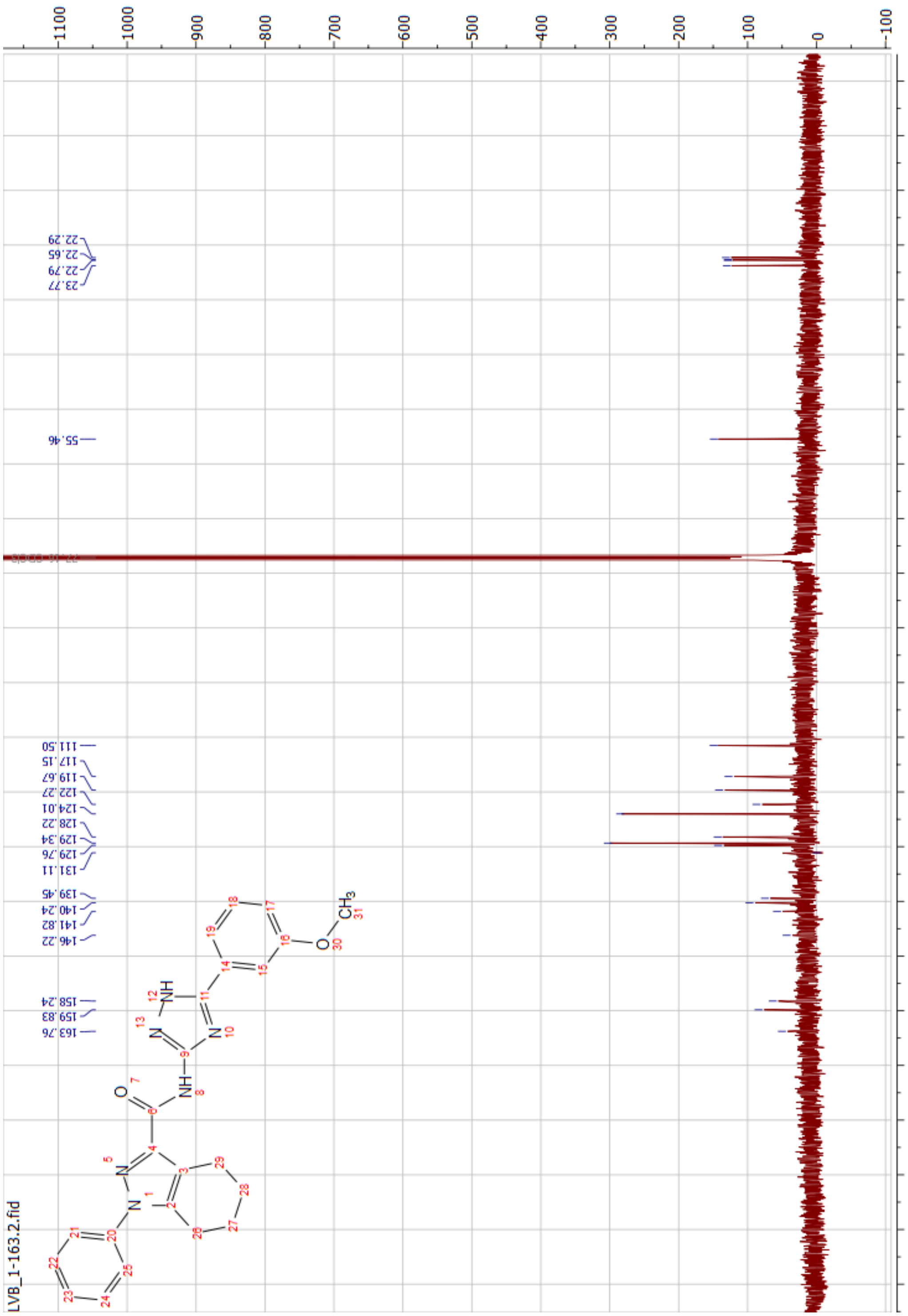

Figure S 6: ^{1}H (400 MHz) and ^{13}C NMR (101 MHz) NMR (DMSO) of N-(5-(3-methoxyphenyl)-1H-1,2,4-triazol-3-yl)-1-phenyl-4,5,6,7-tetrahydro-1H-indazole-3-carboxamide (**32**).

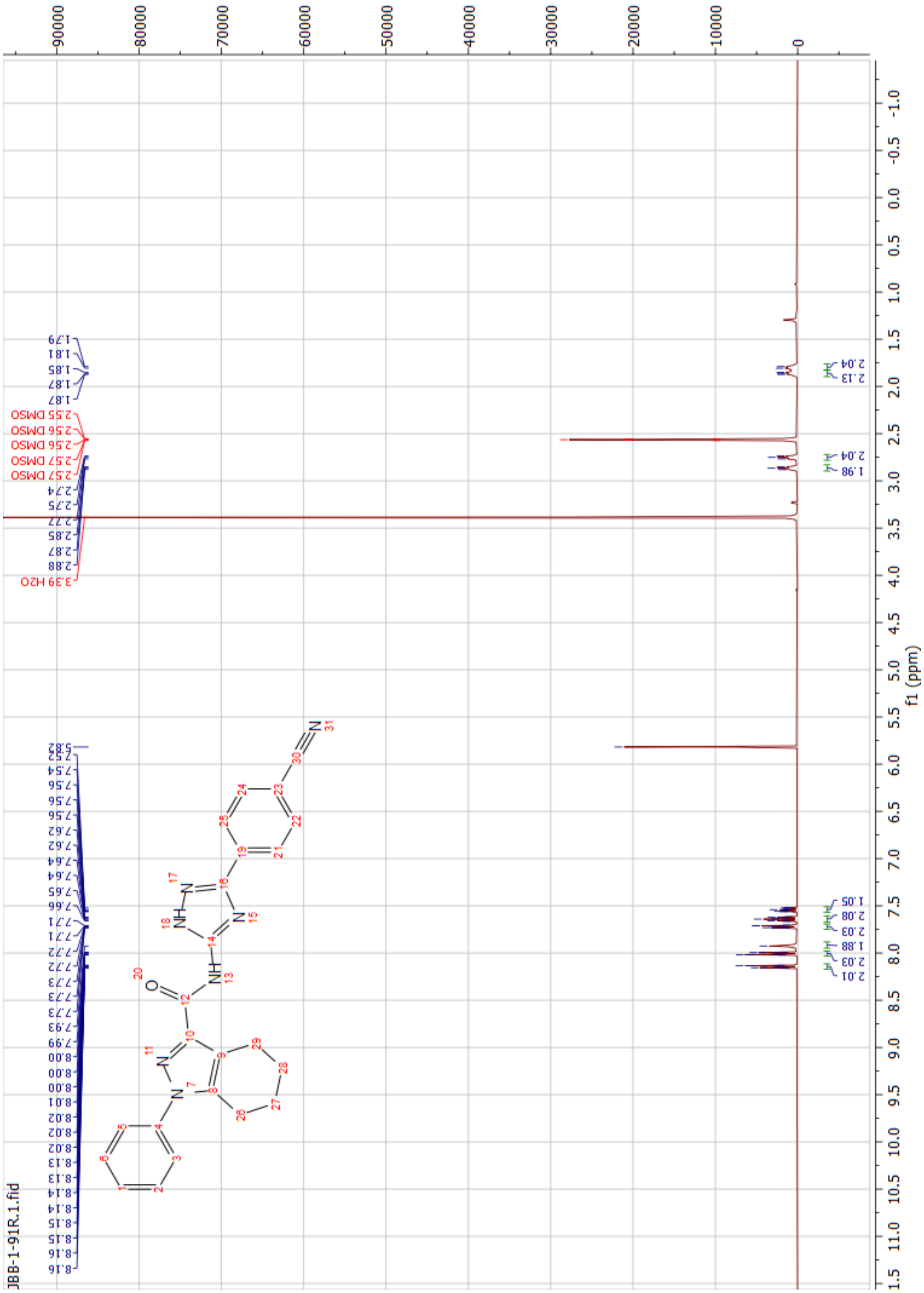
JBB-1-91R.1.fid
f1 (ppm)

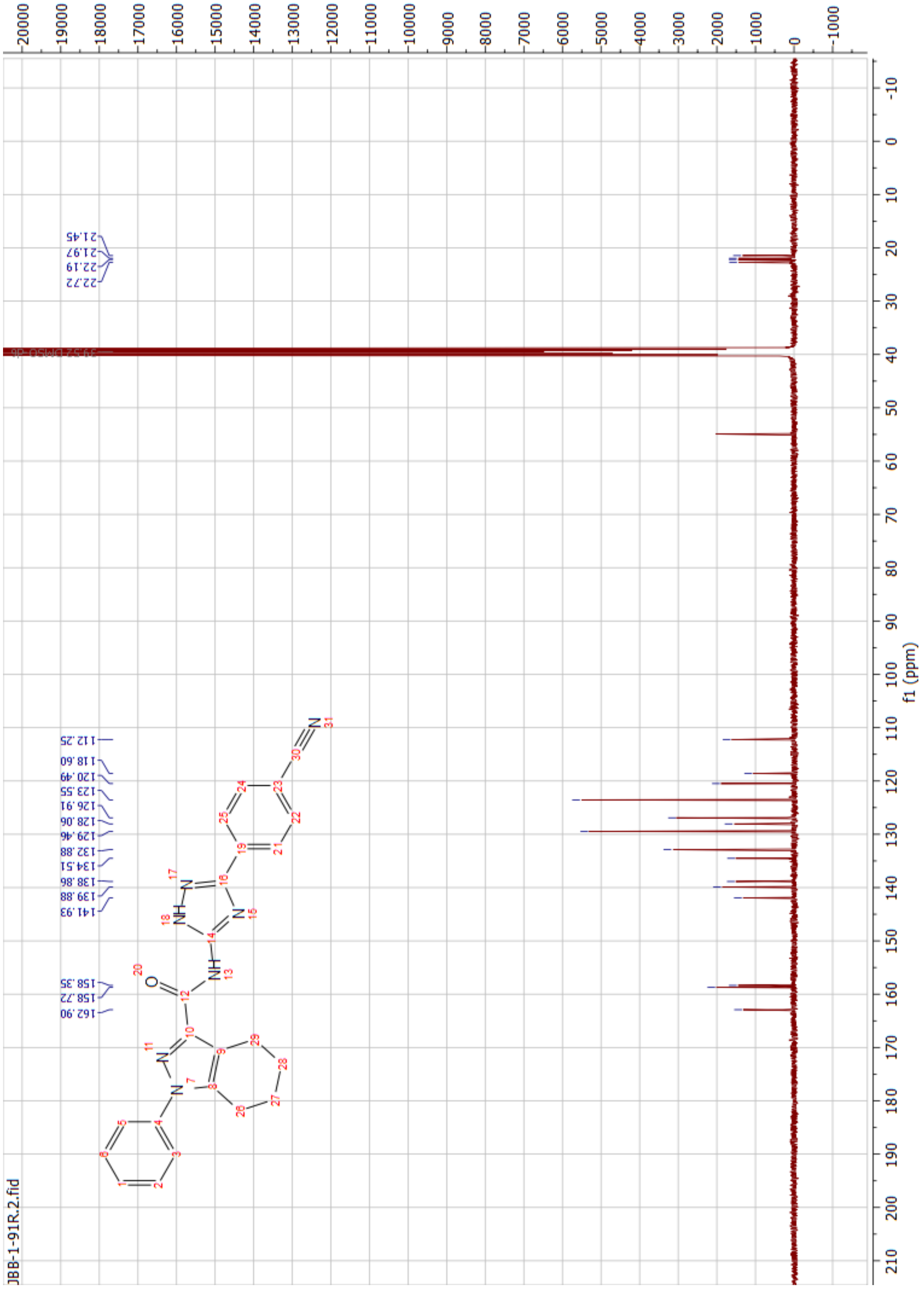

Figure S 7: ^{1}H (400 MHz) and ^{13}C NMR (101 MHz) NMR (DMSO) of N-(5-(4-cyanophenyl)-1H-1,2,4-triazol-3-yl)-1-phenyl-4,5,6,7-tetrahydro-1H-indazole-3-carboxamide (**30**).

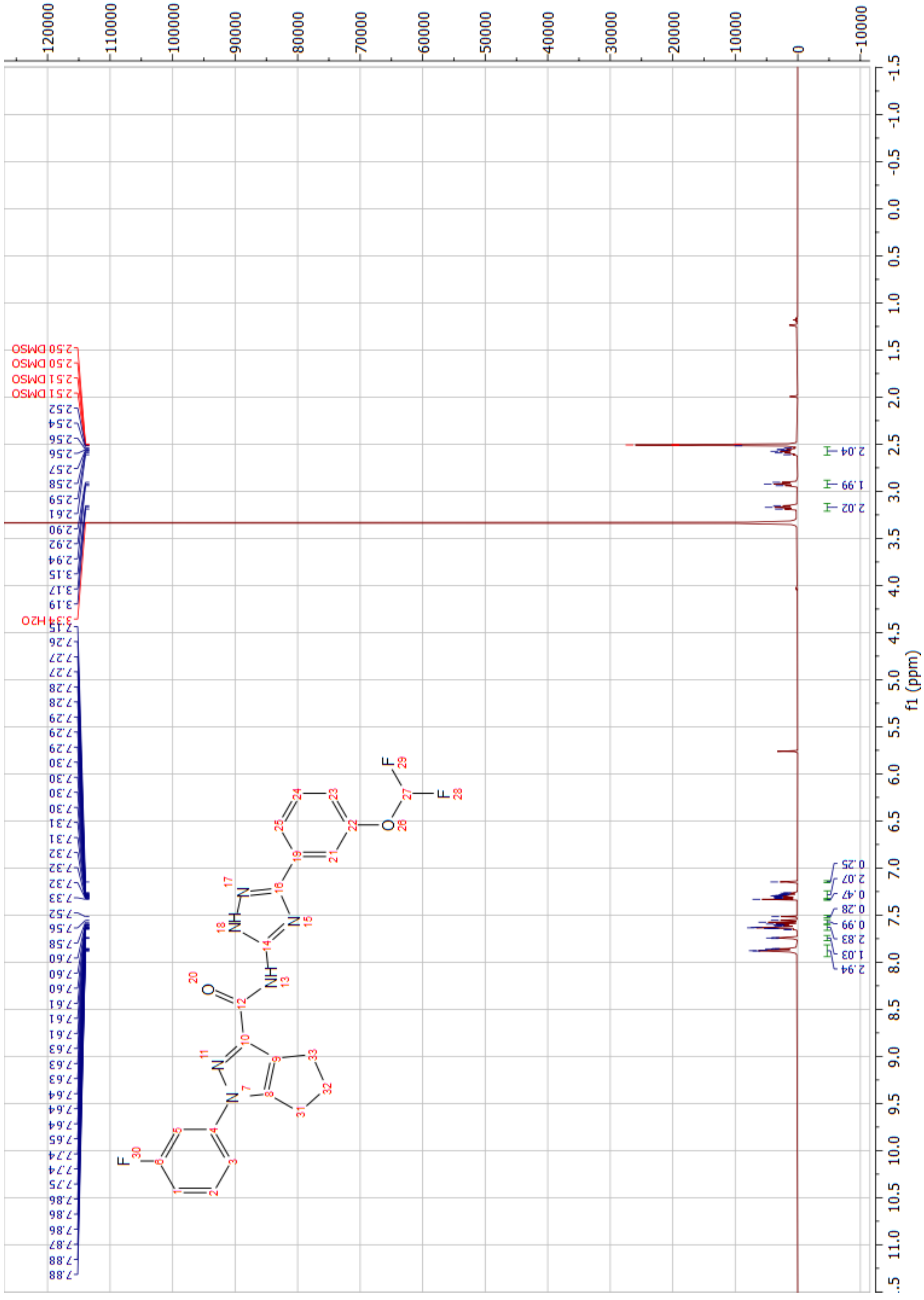
f1 (ppm)
2.50 DMSO
3.34 H2O

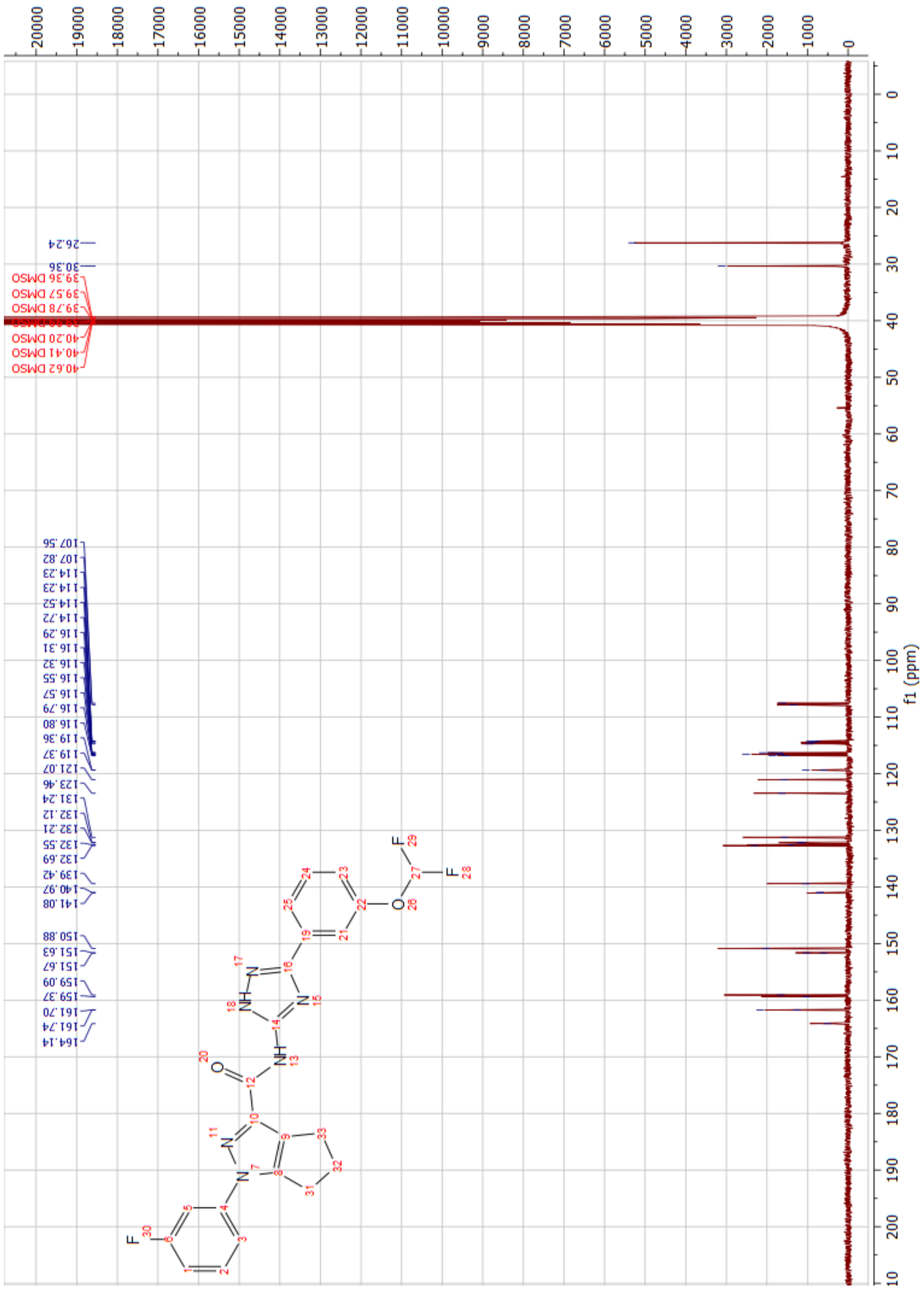

Figure S 8: ^{1}H (400 MHz) and ^{13}C NMR (101 MHz) NMR (DMSO) of N-[3-[3-(difluoromethoxy)phenyl]-1H-1,2,4-triazol-5-yl]-1-(3-fluorophenyl)-5,6-dihydro-4H-cyclopenta[d]pyrazole-3-carboxamide (**18**).

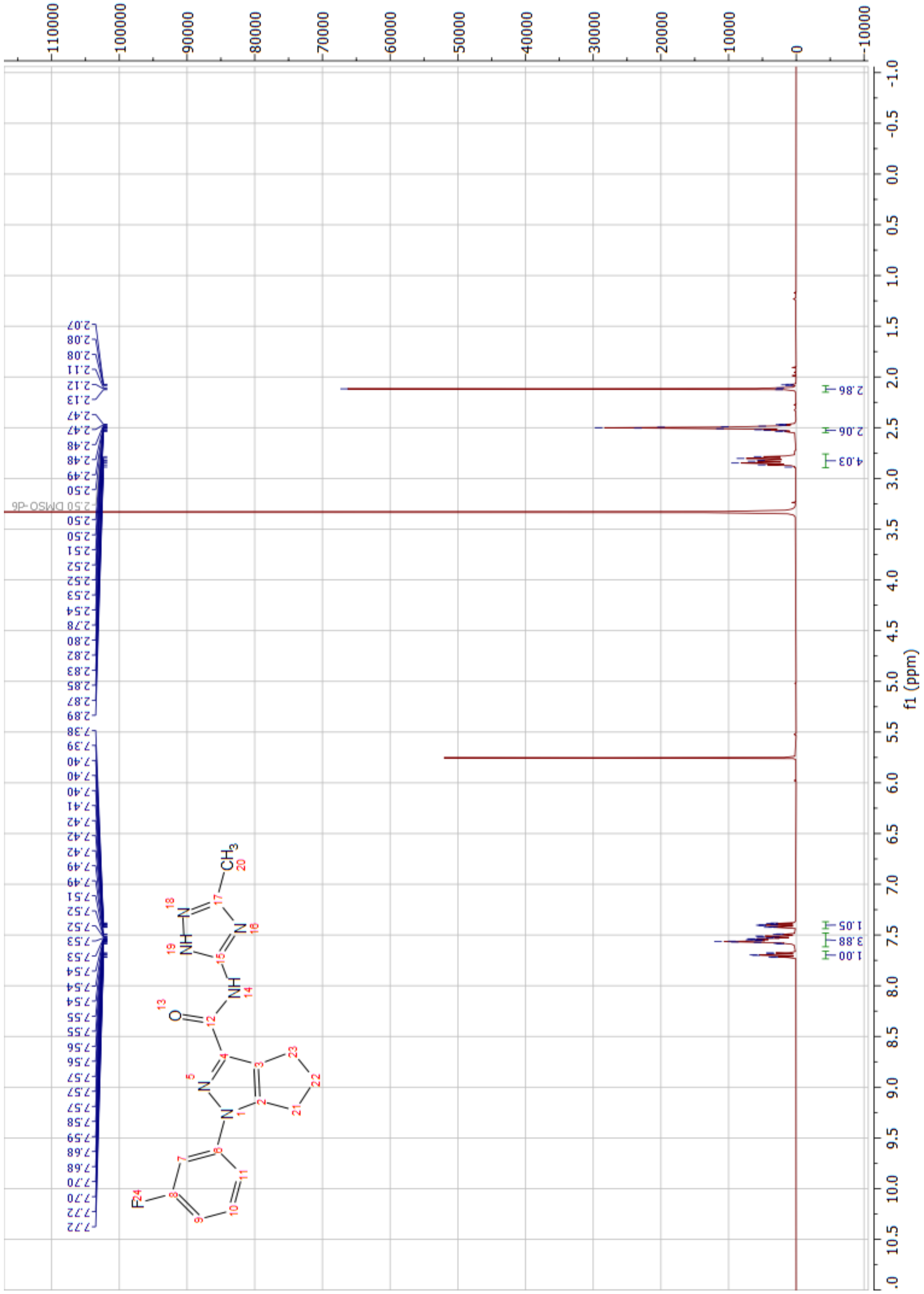
f1 (ppm)
2.50 DMSO-d6
2.86
2.06
4.03
1.05
3.88
1.00

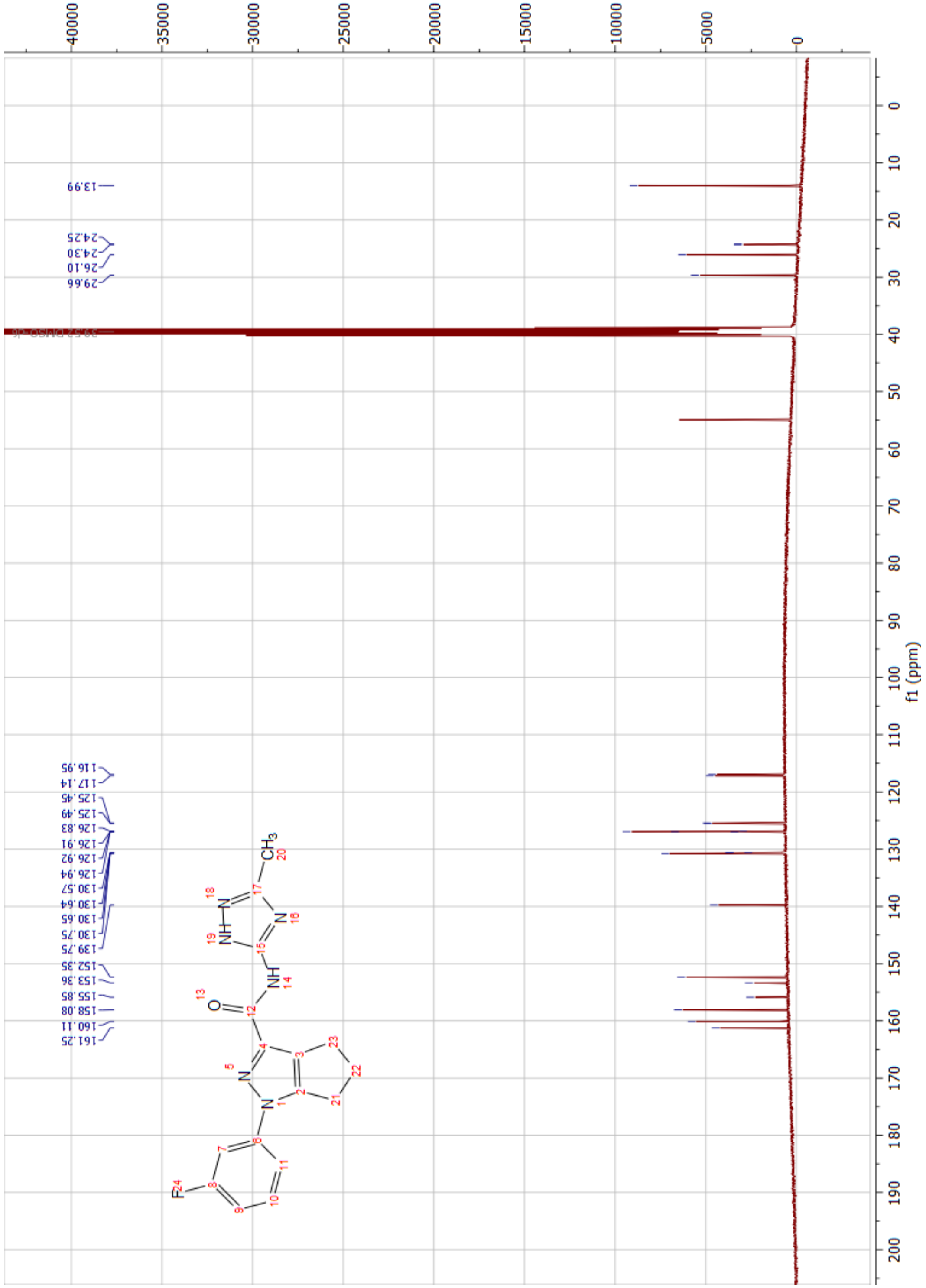

Figure S 9: ^{1}H (400 MHz) and ^{13}C NMR (101 MHz) NMR (DMSO) of 1-(3-fluorophenyl)-N-(3-methyl-1H-1,2,4-triazol-5-yl)-5,6-dihydro-4H-cyclopenta[d]pyrazole-3-carboxamide (**19**).

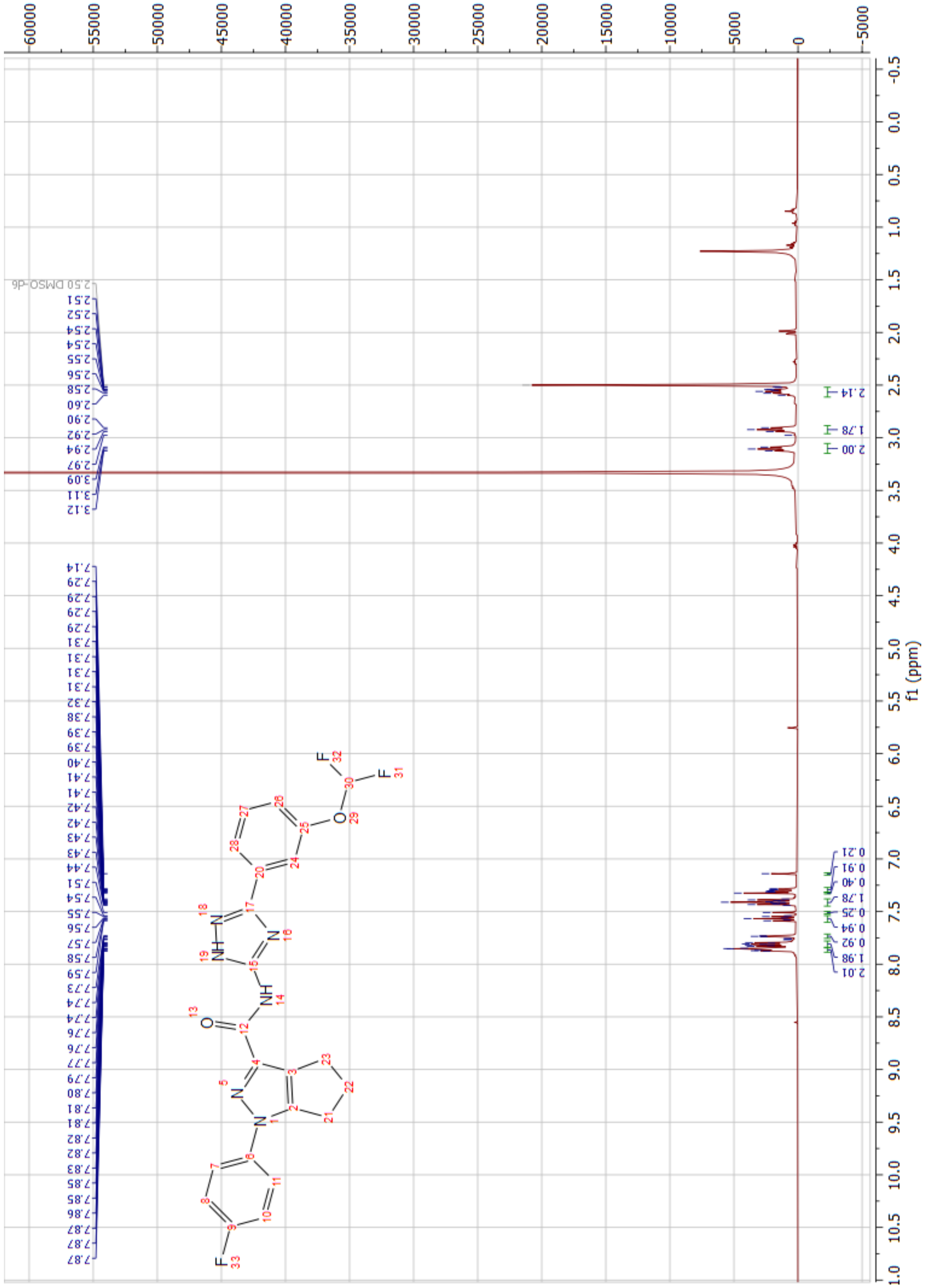

2.50 DMSO-d6
2.51
2.52
2.54
2.54
2.55
2.56
2.58
2.60
2.90
2.92
2.92
2.94
2.97
3.09
3.11
3.12
7.14
7.29
7.29
7.29
7.29
7.31
7.31
7.31
7.31
7.32
7.38
7.39
7.39
7.40
7.41
7.41
7.42
7.42
7.43
7.43
7.44
7.51
7.54
7.55
7.56
7.57
7.58
7.59
7.73
7.74
7.74
7.76
7.76
7.77
7.79
7.80
7.81
7.81
7.82
7.82
7.83
7.85
7.85
7.86
7.87
7.87
7.87
2.14
1.78
2.00
0.21
0.91
0.40
1.78
0.25
0.94
0.92
1.98
2.01
f1 (ppm)

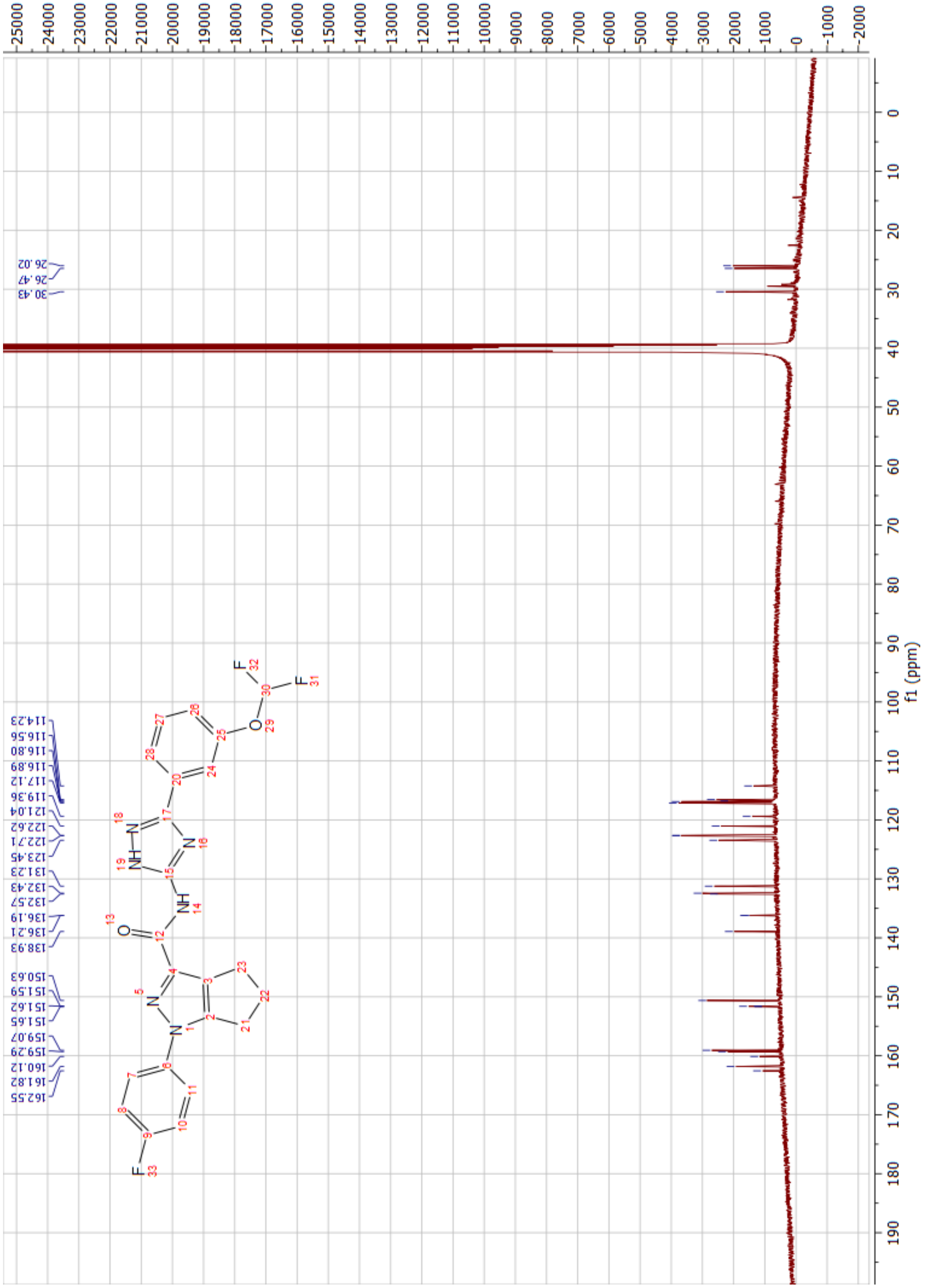

Figure S 10: ^{1}H (400 MHz) and ^{13}C NMR (101 MHz) NMR (DMSO) of N-[3-[3-(difluoromethoxy)phenyl]-1H-1,2,4-triazol-5-yl]-1-(4-fluorophenyl)-5,6-dihydro-4H-cyclopenta[d]pyrazole-3-carboxamide (**20**).

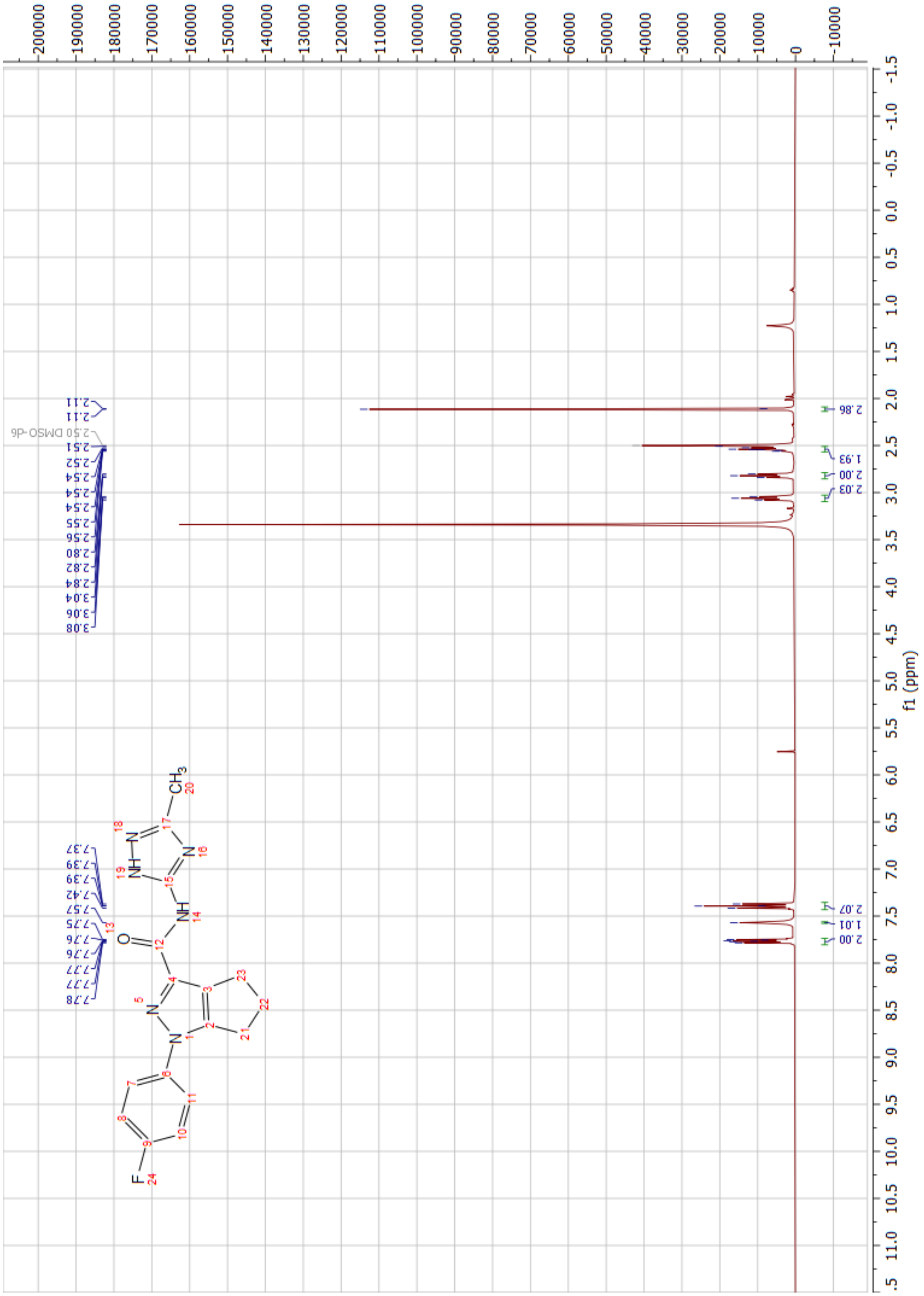

f1 (ppm)
2.50 DMSO-d6

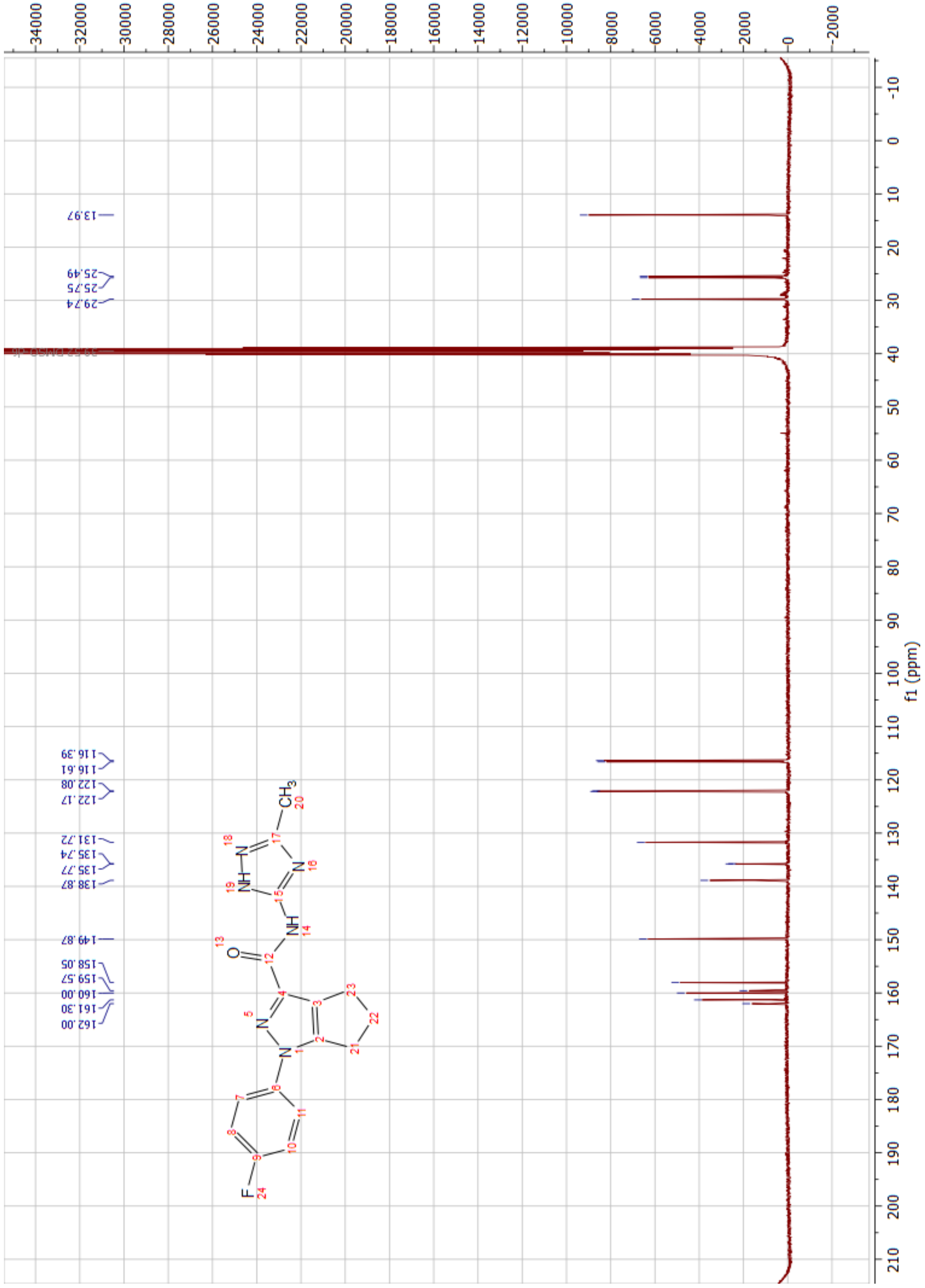

Figure S 11: ^{1}H (400 MHz) and ^{13}C NMR (101 MHz) NMR (DMSO) of 1-(4-fluorophenyl)-N-(3-methyl-1H-1,2,4-triazol-5-yl)-5,6-dihydro-4H-cyclopenta[d]pyrazole-3-carboxamide (**21**).

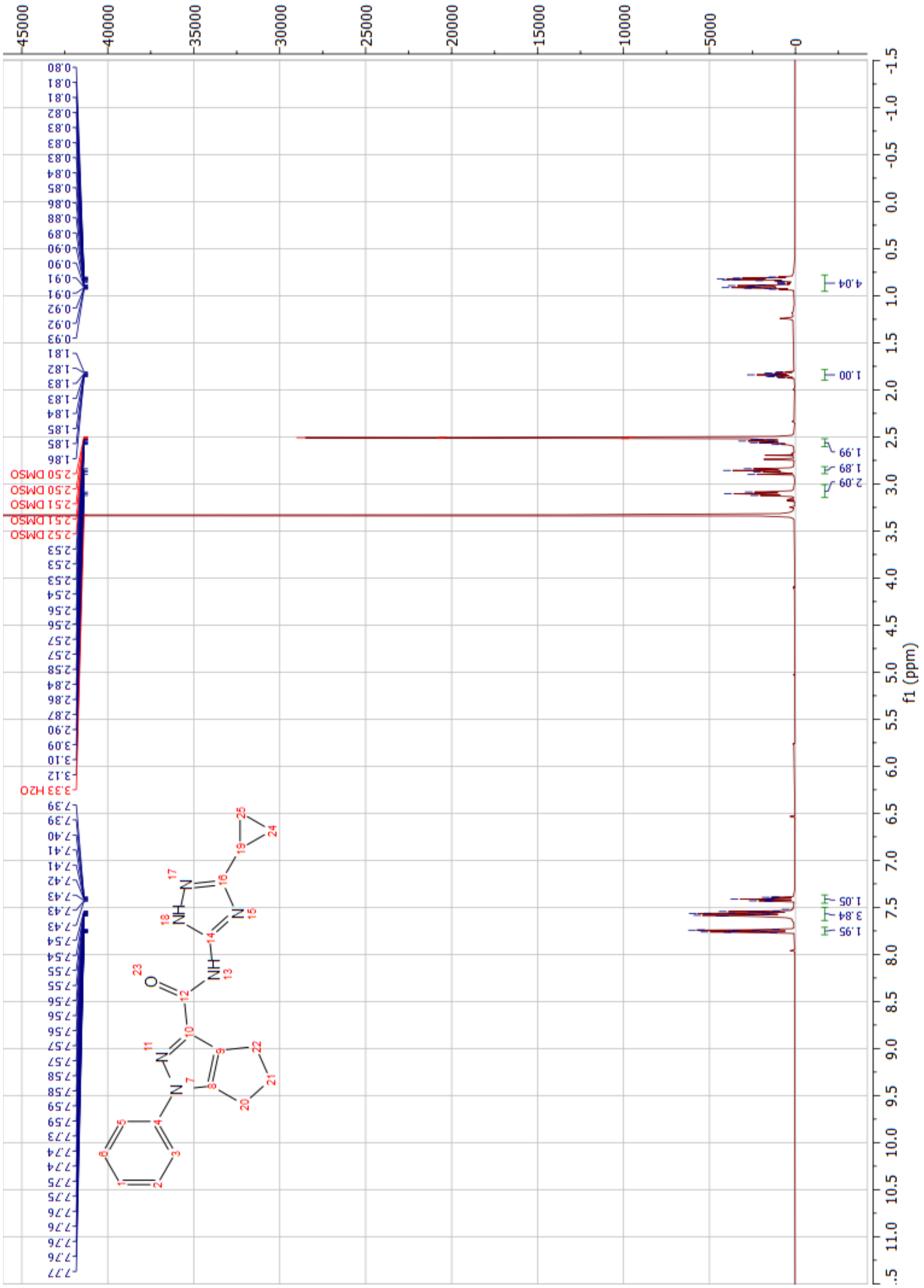
f1 (ppm)
4.04
1.00
1.99
1.89
2.09
1.05
3.84
1.95
2.50 DMSO
2.51 DMSO
2.52 DMSO
3.33 H2O

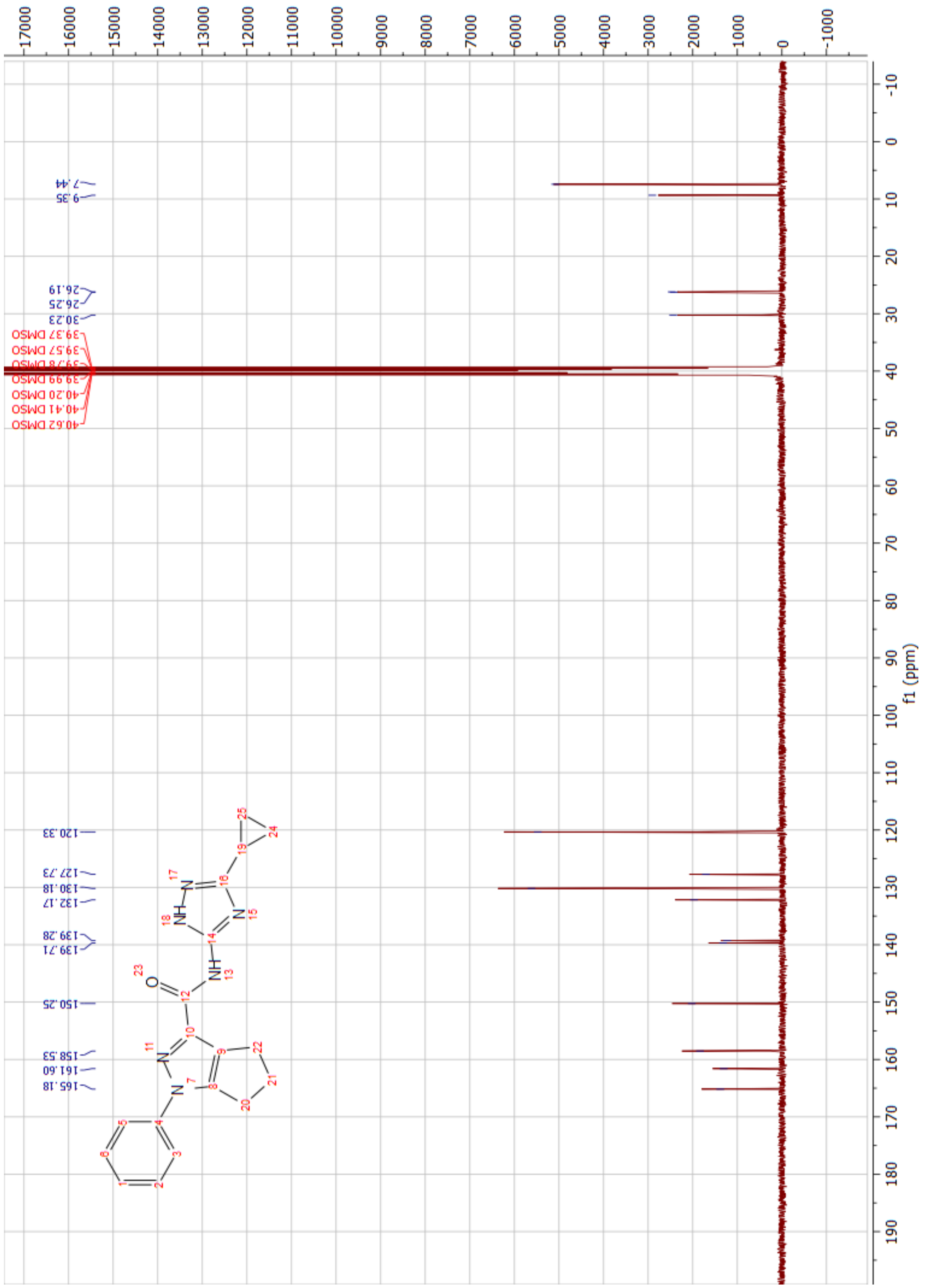

Figure S 12: ^{1}H (400 MHz) and ^{13}C NMR (101 MHz) NMR (DMSO) of N-(3-cyclopropyl-1H-1,2,4-triazol-5-yl)-1-phenyl-5,6-dihydro-4H-cyclopenta[d]pyrazole-3-carboxamide (**24**).

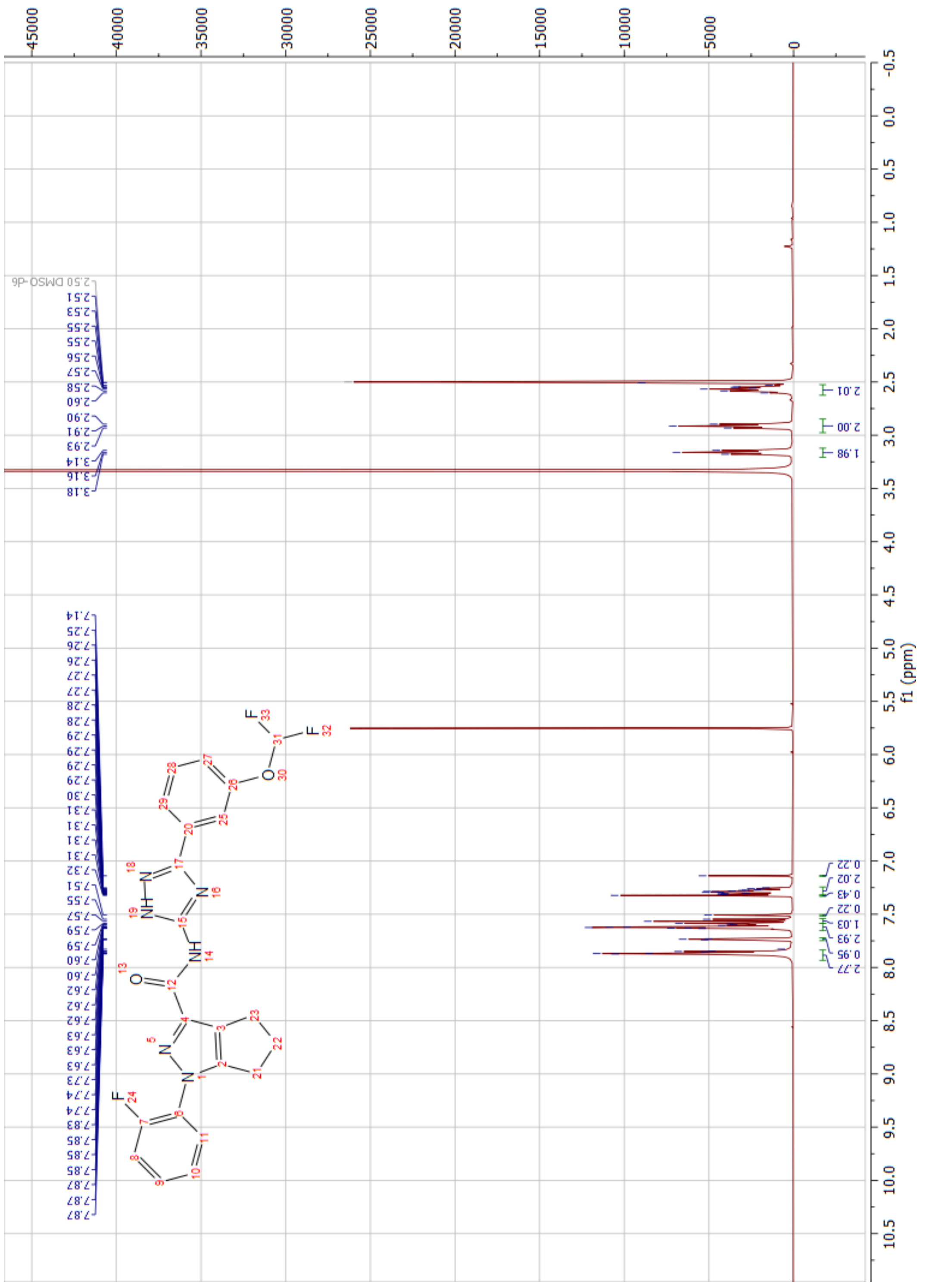
2.50 DMSO-d6
f1 (ppm)

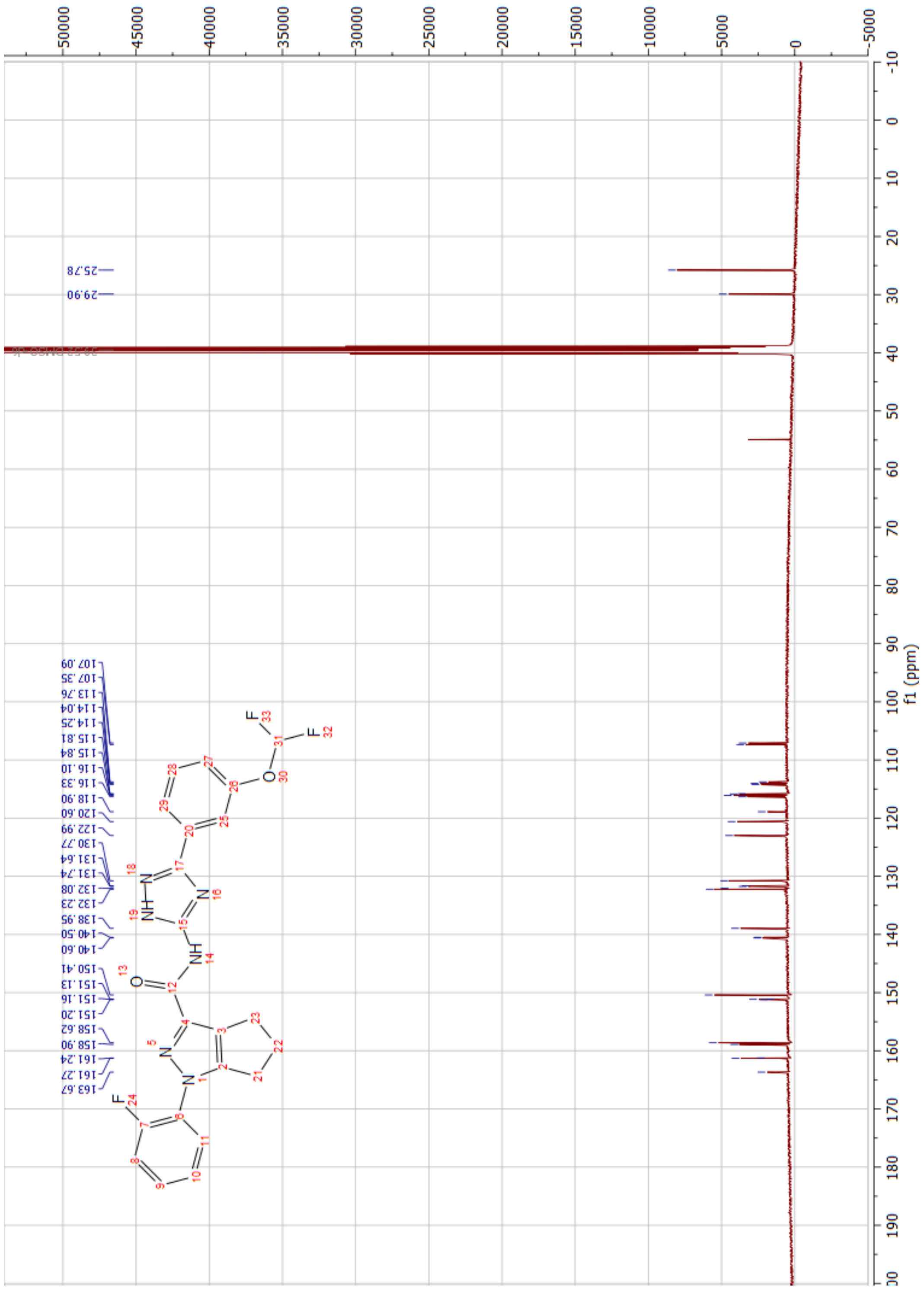

Figure S 13: ^{1}H (400 MHz) and ^{13}C NMR (101 MHz) NMR (DMSO) of N-[3-[3-(difluoromethoxy)phenyl]-1H-1,2,4-triazol-5-yl]-1-(2-fluorophenyl)-5,6-dihydro-4H-cyclopenta[d]pyrazole-3-carboxamide (**23**).

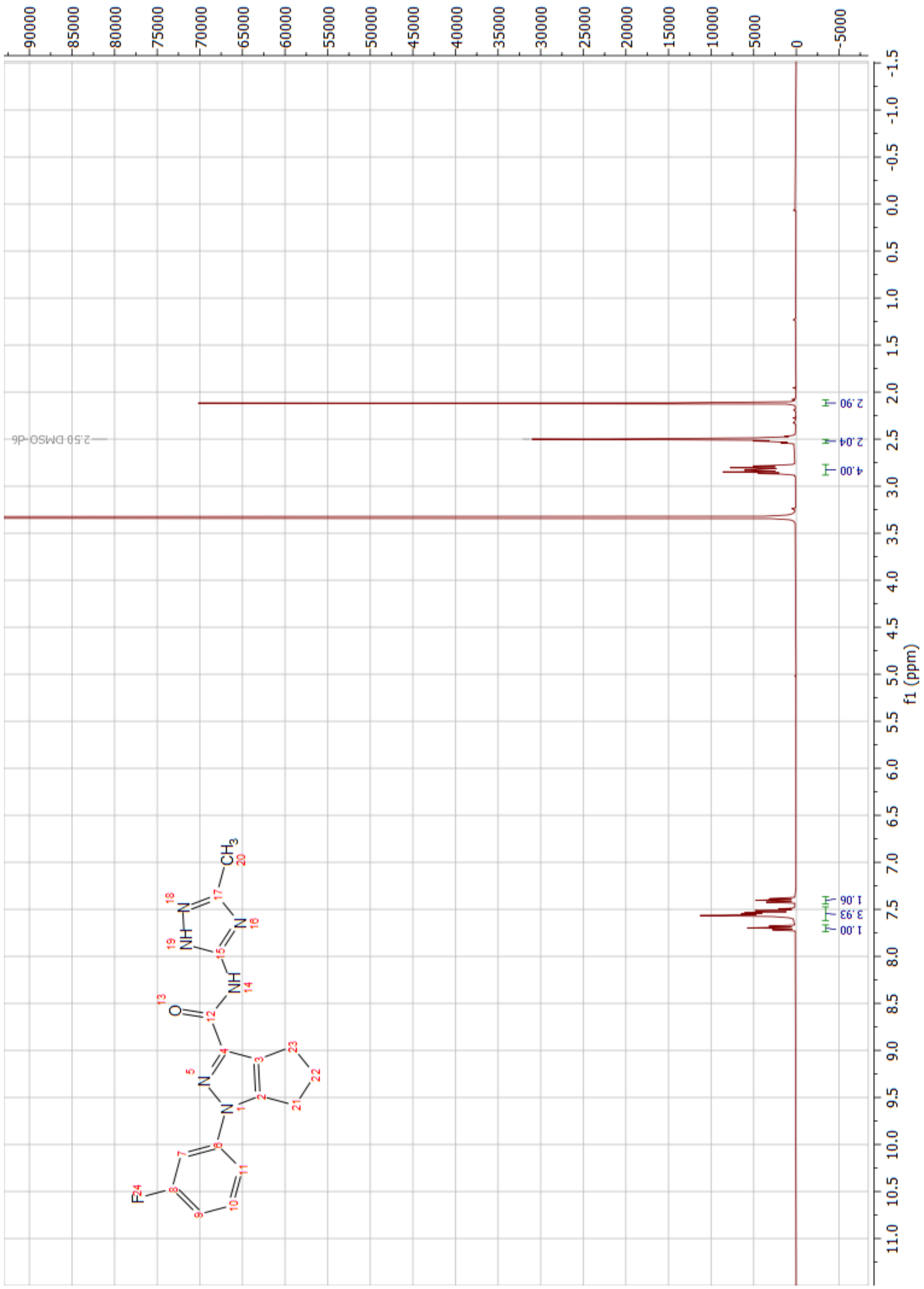
2.50 DMSO-d6
2.90
2.04
4.00
1.06
3.93
1.00
f1 (ppm)
CH3
NH
N
O

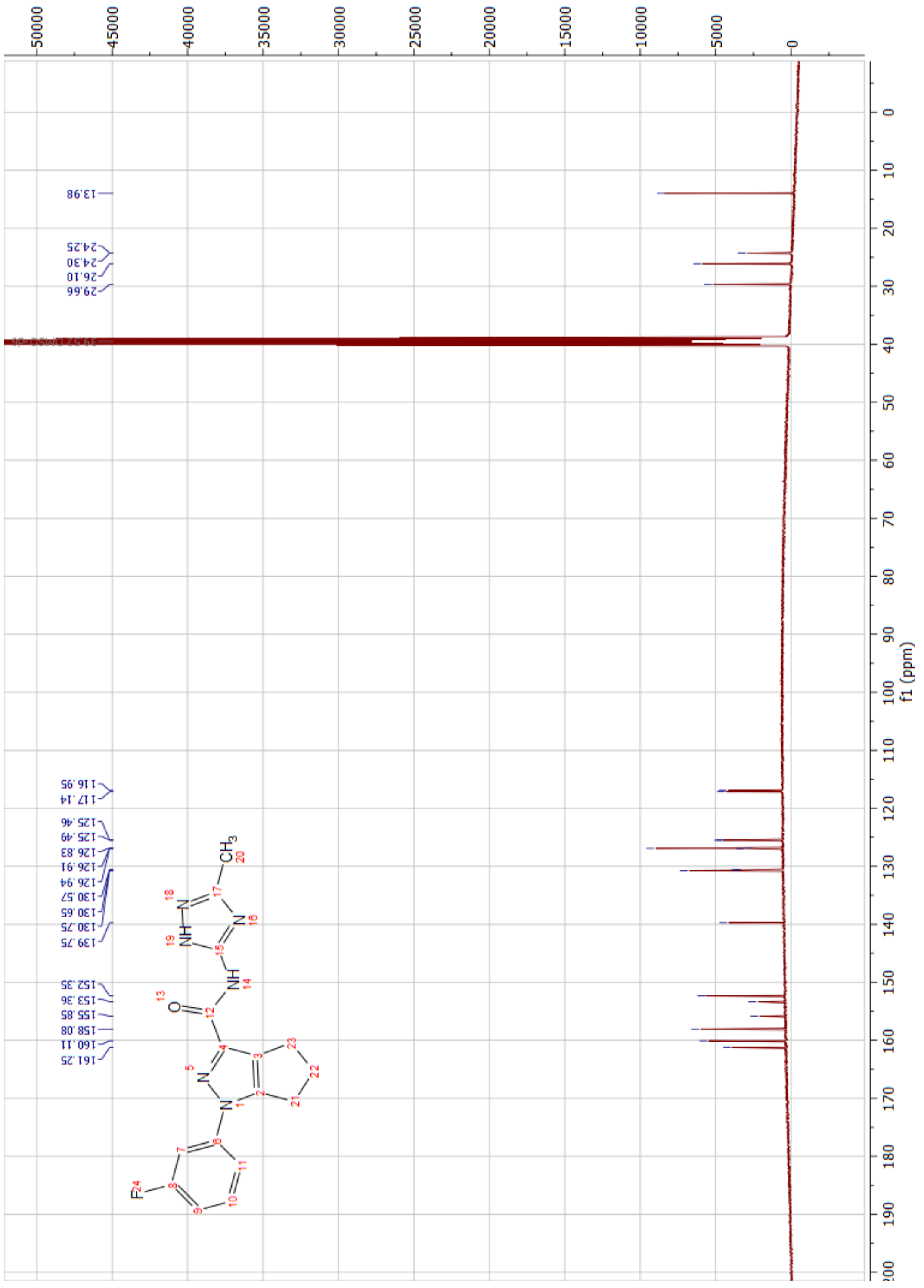

Figure S 14: ^{1}H (400 MHz) and ^{13}C NMR (101 MHz) NMR (DMSO) of 1-(3-fluorophenyl)-N-(3-methyl-1H-1,2,4-triazol-5-yl)-5,6-dihydro-4H-cyclopenta[d]pyrazole-3-carboxamide (**19**).

1.3 Protocol Captures

1.3.1 RosettaGPCR homology modeling of PAR4

Sequence alignments for homology modeling

```
>PAR4
----------------------RALLLGWVPTRLVPALYGLVLVVGLPANGLALWVLATQ-------AP-R--------
LPSTMLLMNLAAADLLLAL-ALPPRIAYHLR-GQ-----------RWPF--------
GEAACRLATAALYGHMYGSVLLLAAVSLDRYLALVH----------------PLRARALR-----------
GRRLALGLCMAAWLMAAALALPLTLQ--RQTFRL--AR------------------------------------------
--SD-RVLCHD---------------------------------------------------------------------
------------------------------------ALP-----------------------------------------
LDAQ----ASHWQPAFTCLALLGCFLPLLAMLLCYGATLHTLAASGR---------------------------------
--------------------------------------------------------------------------------
--------------------------------------------------------------------------------
-------------------------RYGHALRLTAVVLASAVAFFVPSNLLLLLHYSDPSP-S-----------AW----
-GNLYGAYVPSLALSTLNSCVDPFIYYYVSAEFRDKVRAGLFQRSPGDTVASKAS
>5ndd_
----------------------ASVLTGKLTTVFLPIVYTIVFVVALPSNGMALWVFLFR-------TKKK--------
APAVIYMANLALADLLSVI-WFPLKIAYHIH-GN-----------NWIY--------
GEALCNVLIGFFYANMYCSILFLTCLSVQRAWEIVN----------------PMGHSRK------------
KANIAIGISLAIWLLILLVTIPLYVV--KQTIFI--PA------------------------------------------
--LQ-ITTCHD---------------------------------------------------------------------
------------------------------------VLP-----------------------------------------
EQLL----VGDMFNYFLSLAIGVFLFPAFLTASAYVLMIRAL--------------------------------------
--------------------------------------------------------------------------------
--------------------------------------------------------------------------------
--------------------ENSEKKRKRAIKLAVTVAAMYLICFTPSNLLLVVHYFLIKS-Q-----------GQ----
-SHVYALYIVALCLSTLNSCIDPFVYYFVSHDFRDHAKNAL--------------
>3vw7_
----------------------SGYLTSSWLTLFVPSVYTGVFVVSLPLNIMAIVVFILK-------MKVK--------
KPAVVYMLHLATADVLFVS-VLPFKISYYFS-GS-----------DWQF--------
GSELCRFVTAAFYCNMYASILLMTVISIDRFLAVVY----------------PM-----R-----------
TLGRASFTCLAIWALAIAGVVPLLLK--EQTIQV--PG------------------------------------------
--LG-ITTCHD---------------------------------------------------------------------
------------------------------------VLS----------------------------------------
ETLLE----GYYAYYFSAFSAVF-FFVPLIISTVCYVSIIRCLSSSA---------------------------------
--------------------------------------------------------------------------------
--------------------------------------------------------------------------------
----------------------ANRSKKSRALFLSAAVFCIFIICFGPTNVLLIAHYSFLSH-TS----------TT---
--EAAYFAYLLCVCVSSISCCIDPLIYYYASSEC---------------------
```

```
>5lwe_
---------------------------RQFASHFLPPLYWLVFIVGALGNSLVILVYWYC-------ARAK--------
TATDMFLLNLAIADLLFLV-TLPFWAIA------------------------------
TFMCKVVNSMYKMNFYSCVLLIMCICVDRYIAIAQ----------------AMRAHTWREK---------
RLLYSKMVCFTIWVLAAALCIPEILY------------------------------------------------------
--------CT----------------------------------------------------------------------
--------------------------------------------------------------------------------
--------TKLKSAVLALKVILGFFLPFVVMACCYTIIIHTLIQAKK---------------------------------
--------------------------------------------------------------------------------
--------------------------------------------------------------------------------
------------------------SSKHKALKATITVLTVFVLSQFPYNCILLVQTIDAYA-MFI----------SN-
CAVSTAIDICFQVTQAIAFFHSCLNPVLYVFVGERFRRDLVKTLKNLGAISQAAAHH-
>5vbl_
----------------------CEYTDWKSSGALIPAIYMLVFLLGTTGNGLVLWTVFRS-------SREKR-------
RSADIFIASLAVADLTFVV-TLPLWATYTYR-DY-----------DWPF--------
GTFFCKLSSYLIFVNMYASAFCLTGLSFDRYLAIVR----------------PVANARLR-----------
LRVSGAVATAVLWVLAALLAMPVMVL--RTTGDL--EN------------------------------------------
--TN-KVQCYM---------------------------------------------------------------------
------------------------------------DYS--------------------------------------
MVATVSSE----WAWEVGLGVSSTTVGFVVPFTIMLTCYFFIAQTIA---------------------------------
--------------------------------------------------------------------------------
--------------------------------------------------------------------------------
------------------------------RRRLLSIIVVLVVTFALCKMPYHLVKTLYMLGSLL-H------------
WP-CDFDLFLMNIFPYCTCISYVNSCLNPFLYAFFDPRFRQACTSMLLMGQSR--------
>6c1r_
------------------------SNTLRVPDILALVIFAVVFLVGVLGNALVVWVTAFE-------AK-R--------
TINAIWFLNLAVADFLSCL-ALPILFTSIVQ-HH-----------HWPF--------
GGAACSILPSLILLNMYASILLLATISADRFLLVFK----------------PIWCQNFR-----------
GAGLAWIACAVAWGLALLLTIPSFLY--RVVREE--YF------------------------------------------
--PP-KVLCGV---------------------------------------------------------------------
-------------------------------------------------------------------------------
DYSHD----KRRERAVAIVRLVLGFLWPLLTLTICYTFILLRTWSRR---------------------------------
--------------------------------------------------------------------------------
--------------------------------------------------------------------------------
---------------------------STKTLKVVVAVVASFFIFWLPYQVTGIMMSFL----EPS--------SPTF---
--LLLKKLDSLCVSFAYINCCINPIIYVVAGQGF-----KSLPSLLRNVLTEES--
```

1.3.2 Running RosettaGPCR

```
> $ROSETTA-3.10/main/source/bin/rosetta_scripts.default.linuxgccrelease @ flags
```

flags

```
# i/o
-in:file:fasta target.fasta
-nstruct 10
-parser:protocol rosetta_cm.xml
-out:pdb_gz
-in:file:extra_res_fa BMS.fa.params
-in:file:extra_res_cen BMS.cen.params
-extra_improper_file BMS.fa.tors

# membrane options
-in:file:spanfile span.txt
-membrane:no_interpolate_Mpair
-membrane:Menv_penalties
-rg_reweight .1

# relax options
-default_max_cycles 200
-relax:min_type lbfgs_armijo_nonmonotone
-relax:minimize_bond_angles
-relax:minimize_bond_lengths
-relax:jump_move true
-score:weights stage3_rlx_membrane.wts
-use_bicubic_interpolation
-hybridize:stage1_probability 1.0
-sog_upper_bound 15
-out:level 100
-linmem_ig 10
```

disulf.txt

```
82 161
```

span.txt

```
TM region prediction for target.topo predicted using OCTOPUS
7 303
antiparallel
n2c
  12    32    12    32
  47    67    47    67
  81   111    81   111
 124   144   124   144
 178   198   178   198
 216   236   216   236
 253   273   253   273
```

rosettacm.xml

```
<ROSETTASCRIPTS>
    <TASKOPERATIONS>
    </TASKOPERATIONS>
    <SCOREFXNS>
        <ScoreFunction name="stage1" weights="stage1_membrane.wts"
symmetric="0">
            <Reweight scoretype="atom_pair_constraint" weight="1"/>
        </ScoreFunction>
        <ScoreFunction name="stage2" weights="stage2_membrane.wts"
symmetric="0">
            <Reweight scoretype="atom_pair_constraint" weight="0.5"/>
        </ScoreFunction>
        <ScoreFunction name="fullatom" weights="stage3_rlx_membrane.wts"
symmetric="0">
            <Reweight scoretype="atom_pair_constraint" weight="0.5"/>
        </ScoreFunction>
        <ScoreFunction name="membrane"
weights="membrane_highres_Menv_smooth" symmetric="0">
            <Reweight scoretype="cart_bonded" weight="0.5"/>
            <Reweight scoretype="pro_close" weight="0"/>
        </ScoreFunction>
    </SCOREFXNS>
    <FILTERS>
    </FILTERS>
    <MOVERS>
        <Hybridize name="hybridize" stage1_scorefxn="stage1"
stage2_scorefxn="stage2" fa_scorefxn="fullatom" batch="1"
stage1_increase_cycles="1.0" stage2_increase_cycles="1.0" linmin_only="1"
realign_domains="0"
```

```
disulf_file="/dors/meilerlab/home/smithst/par4/par4_remodel/rosettagpcr/r
osetta_cm/disulf.txt" add_hetatm="1">
            <Template
pdb="/dors/meilerlab/home/smithst/par4/par4_remodel/rosettagpcr/rosetta_c
m/5ndd_aligned_with_vora.pdb" cst_file="AUTO" weight="1.000"/>
            <Template
pdb="/dors/meilerlab/home/smithst/par4/par4_remodel/rosettagpcr/rosetta_c
m/3vw7_aligned_with_vora.pdb" cst_file="AUTO" weight="1.000"/>
            <Template
pdb="/dors/meilerlab/home/smithst/par4/par4_remodel/rosettagpcr/rosetta_c
m/5lwe_aligned_with_vora.pdb" cst_file="AUTO" weight="1.000"/>
            <Template
pdb="/dors/meilerlab/home/smithst/par4/par4_remodel/rosettagpcr/rosetta_c
m/5vbl_aligned_with_vora.pdb" cst_file="AUTO" weight="1.000"/>
            <Template
pdb="/dors/meilerlab/home/smithst/par4/par4_remodel/rosettagpcr/rosetta_c
m/6c1r_aligned_with_vora.pdb" cst_file="AUTO" weight="1.000"/>
        </Hybridize>
        <ClearConstraintsMover name="clearconstraints"/>
    </MOVERS>
    <APPLY_TO_POSE>
    </APPLY_TO_POSE>
    <PROTOCOLS>
        <Add mover="hybridize"/>
        <Add mover="clearconstraints"/>
    </PROTOCOLS>
    <OUTPUT scorefxn="membrane"/>
</ROSETTASCRIPTS>
```

1.3.3 Structure optimization for ultra-large screening (ULS) in DOCK

Run initial blastermaster on PAR4 across all RosettaCM outputs

```
# In std_dockprep/ generate initial blastermaster using defaults
egrep ATOM PAR4_BMS_...pdb > rec.pdb
egrep HETATM PAR4_BMS...pdb > xtal-lig.pdb
egrep CONECT PAR4_BMS...pdb >> xtal-lig.pdb
source trunk
$DOCKBASE/protein/blastermaster.py -v -addhOptions='-HIS -FLIPs '

# Run blastermaster in each ES/LD setting
# Use FULL paths!
csh /path/to/script/dockprep_thin_spheres_in_batches.csh /path/to/script/
/path/to/std_dockprep

# In ligands-decoys/ add ligands and decoys files
ls FULLPATH/TO/ACTIVES_DB2 > ligands.sdi
ls FULLPATH/TO/DECOYS_DB2 >> ligands.sdi
cat FULLPATH/TO/ACTIVES/input.ism > actives.smi
cat FULLPATH/TO/DECOYS/input.ism > decoys.smi
awk '{print $2}' actives.smi > actives.names
awk '{print $2}' decoys.smi > decoys.names

# PARAMETER SCANNING
# In script/ edit lig-decoy_enrichment_submit.csh, combine...csh, AUC...csh
files to make sure paths are correct
# Back up in thin_spheres_parameter_scanning/
csh FULLPATH/TO/SCRIPT/lig-decoy_enrichment_submit.csh
csh FULLPATH/TO/SCRIPT/combine...csh
# Remember the only things we're changing in each iteration are the
electrostatics and desolvation grids--NOT vdw. The same percentage of
compounds will be able to be docked each time, as this is only dependent on
VDW grid.
# In INDOCK, the #mol2 line is commented out (as usual), atom_maximum set to
100 (as usual)
# With bump_maximum at 10, only 12.5% of compounds were able to be docked so I
increased the bump_maximum from 10 --> 100 in INDOCK.
# bump maximum --> 100, 39.4% of compounds were being docked.
```

PAR4 ligand and decoy preparation

Actives were taken from ChEMBL (PAR4_CHEMBL_actives.smi, n=439) and actives from current Hamm series (PAR4_matt_cmpds_actives_only.smi, n=12) where IC50 < 200 nM. Cat'd these together and full list is located in PAR4_actives_withname.csv to be used for clustering, etc.

PAR4 actives clustering

```
source /nfs/soft/dock/versions/dock37/DOCK-3.7-trunk/env.csh
mkdir /scratch/shannon/
awk '{print $1}' PAR4_actives_withname.csv > label.txt
python /nfs/home/tbalius/zzz.scripts/tanimoto_cal_axon.py -one
PAR4_actives_withname.csv tanimoto.similarity
python
~tbalius/zzz.github/ChemInfTools/utils/teb_chemaxon_cheminf_tools/heatmap_
matrix_cluster.py tanimoto.similarity.tanimoto.matrix label.txt 0.5
complete 1
#0.5 = 1-Tc. 0.5 is typical place to start. Adjust as necessary to obtain
desired number of clusters. Generally ~20-30 seems to be good since we
generate 50 decoys/active.
# 46 cluster centers were generated here and we used these to get decoys
on the DUD-E server using SMILES strings.
```

Generating decoys based on actives

```
# All scripts for this are located in
/mnt/nfs/home/rstein/zzz.scripts/new_DUDE_SCRIPTS/
# source python and Jchem
source /nfs/soft/python/envs/complete/current/env.csh
source /nfs/soft/jchem/current/env.csh
# In dir, need one file for actives smiles looking like this: [SMILES NAME]
# I just catted the actives that came out of the ligand setup (supposed to work
without having protonated SMILES if you designate PROTONATE YES in
decoy_generation.in, but it wasn't working. The bash script it points to was
giving the error: STORE_PROTOMERS NOT FOUND and yielded no output.
cat ~/par4/par4_ligands/dude-
decoys/actives/jobs/x/outputs/x/finished/*/protomers.xls | sed '/name/d' | awk
'{print $1,$2} > actives.smi
# Just put ligands into appropriate sub-directories in {NEW_DIR_NAME} aka
"ligands_dir" here.
python
/mnt/nfs/home/rstein/zzz.scripts/new_DUDE_SCRIPTS/0000_protonate_setup_dirs.py
actives.smi {NEW_DIR_NAME}
# Submit to get ZINC15 decoys SMILES. Number of decoys to generate set to default
20-50 with Tc_max = 0.8. Just look in decoy_generation.in
# Takes some time--only about 15 minutes for 46 actives.
python
/mnt/nfs/home/rstein/zzz.scripts/new_DUDE_SCRIPTS/0001_qsub_generate_decoys.py
{NEW_DIR_NAME}
#
python
/mnt/nfs/home/rstein/zzz.scripts/new_DUDE_SCRIPTS/0002_qsub_filter_decoys.py
{NEW_DIR_NAME}
python
/mnt/nfs/home/rstein/zzz.scripts/new_DUDE_SCRIPTS/0003b_write_out_ligands_decoys.
py {NEW_DIR_NAME} ligands-decoys/
# Now have ligands-decoys/decoys/ dir
# $ph set to 7.4
# NOTE: for 2250 decoys, this took overnight!
${DOCKBASE}/ligand/generate/build_database_ligand.sh -H $ph decoy_protomers.smi -
-pre-tautomerized --no-db
# Plot properties (MW, HBD, HBA, N_rots, logP, charge) of actives vs. decoys
python /mnt/nfs/home/rstein/zzz.scripts/new_DUDE_SCRIPTS/0004_plot_properties.py
{NEW_DIR_NAME}
```

Generating ligand files for DOCK:

```
source /nfs/soft/dock/versions/dock37/DOCK-3.7-trunk/env.csh
After generating decoys using DUD-E server, we generate ligand files for DOCK:
*MAKE SURE THE LIGANDS ARE NAMED WITH AT LEAST 3 DIGITS! Use either ChEMBL name
or arbitrary names starting from 100*
{code:title=Creating ligand files for decoys and actives}
# expand downloaded file. in decoys/ :
# take the decoys files, remove first "ligand" line and extract first 2 columns
cat decoys.P*.picked | sed '/ligand/d' | awk '{print $1,$2}' > decoys.smi
# copy setup and run command files from Elissa's dir
cp /nfs/ex9/work/elisfink/scripts/ligand-prep/cmd .
cp /nfs/ex9/work/elisfink/scripts/ligand-prep/setup .
source setup
csh cmd decoys.smi
```

```
# Do the same prep for actives in dude_decoys/ :
mkdir actives/
cd actives/
awk '{print $1,$2}' ../ligands.charge > actives.smi
# using same cmd, setup scripts
source setup
csh cmd actives.smi
```

Enrichment docking control

```
# link dockfiles/ files
ls -s ../dockfiles .
# need INDOCK output from initial blastermaster here.
cp ../INDOCK .
# Make sure mol2_score_maximum line is commented out. This makes sure that
the score is still recorded even if it's awful.
# set atom_maximum from 25 --> 100.
# Get paths to both actives and decoys to run DOCK in parallel
ls /mnt/nfs/ex9/work/shannon/par1/par1_ligands/dude-
decoys/actives/jobs/x/outputs/x/finished/*/0.db2.gz > enrichment.sdi
ls /mnt/nfs/ex9/work/shannon/par1/par1_ligands/dude-
decoys/actives/jobs/x/outputs/x/finished/CHEMBL*/0.db2.gz >> enrichment.sdi

# Similar to above, we don't want to just loop across all models to run the
control. Instead, submit to cluster by:
bash qsub_dock.csh # links to qsub_dock.csh --> make sure leading 0s match
up and make sense in both files!

# point to names of actives and decoys
egrep actives enrichment.sdi | awk -F '/' '{print $(NF-1)}' > ligands.names
egrep -v actives enrichment.sdi | awk -F '/' '{print $(NF-1)}' >
decoys.names

# Again, this needs to be run in 3 parts based on leading 0s. Edit in
foreach line and the workdir line.
csh enrichment_calcs.csh
# This goes into each directory specified, calculates enrichments using
ligands.names and decoys.names
```

Receptor file used for ULS: S_28_0004 in /mnt/nfs/ex9/work/shannon/par4/rec_files/bms_par4_model/rosettacm_outputs/ (see https://confluence.csb.vanderbilt.edu/display/MeilerLab/PAR4+enrichment+testing)

1.3.4 Post-ULS filtering

Get distribution of scores.

The top ~0.4% of compounds contained scores less than -40, which leaves 480214 poses to analyze. -40 was also the suggested cutoff from the Shoichet lab protocol so I'm going with this. Using the same output from Screen Setup and Submission as above, but the Cluster Results and Filter for Novelty is where we're changing things this time.

Remove compounds that are similar to known actives

```
## Located in /mnt/nfs/ex9/work/shannon/par4/uls/uls-1/leadlike/docking/
mkdir best_first_clustering_100000_1.0/
cd best_first_clustering_100000_1.0/

# cluster DOCKed compounds by tanimoto, I believe *THIS IS WHERE WE DON'T
CLUSTER* --EITHER SET TC=0/1 (CAN'T EVER REMEMBER WHICH DIRECTION IT GOES)
THEN USE THE SAME PROTOCOL SO WE KEEP CONSISTENT. WE WANT ALL THE -40
COMPOUNDS STILL.
#csh
/mnt/nfs/home/jklyu/zzz.script/large_scale_docking/cluster_analysis/best_firs
t_clustering_new.csh 100000 **1.0**
# Instead of the above step, just took the ZINCIDs
from ../best_first_clustering_100000_0.5/ and made the cluster_head* files
using all 100000.

# Remove compounds similar to known actives
mkdir filtering
cd filtering/
ln -s ../cluster_head.zincid .

# I've already done this part for the knowns. Just
copy ../../best_first_clustering_100000_0.5/filtering/knowns* .
##=========================================================
# Copy the smiles and fingerprints of the active molecules to compare to the
cluster heads.
#awk '{print $1}' /mnt/nfs/ex9/work/shannon/par4/par4_ligands/dude-
decoys/actives/actives.smi > knowns_list.smi
# Get Fingerprints for actives--followed protocol used to do this for docked
compounds. Not sure if this is right because it's a nxn matrix, unlike in the
example. But the .group file generation works from this. And not sure how to
read this.
#python
/mnt/nfs/home/jklyu/zzz.github/ChemInfTools/utils/teb_chemaxon_cheminf_tools/
generate_chemaxon_fingerprints.py knowns_list.smi knowns_uint16

# Generate .count file--not sure what this does
#/mnt/nfs/home/jklyu/zzz.github/ChemInfTools/utils/convert_fp_2_fp_in_16unit/
convert_fp_2_fp_in_uint16 knowns_uint16.fp knowns_list.smi knowns_list
##=========================================================

## Cluster DOCKed compounds
```

```
# Generate smiles strings for cluster heads
python
/mnt/nfs/home/jklyu/zzz.github/ChemInfTools/utils/teb_chemaxon_cheminf_tools/
run.getstuff.from.postgreSQL.zinc_faster.py uncluster_head.zincid
uncluster_head.smi

# Generate molecular fingerprints for the cluster heads
python
/mnt/nfs/home/jklyu/zzz.github/ChemInfTools/utils/teb_chemaxon_cheminf_tools/
generate_chemaxon_fingerprints.py uncluster_head.smi uncluster_head
/mnt/nfs/home/jklyu/zzz.github/ChemInfTools/utils/convert_fp_2_fp_in_16unit/c
onvert_fp_2_fp_in_uint16 uncluster_head.fp uncluster_head.smi uncluster_head

# Calculate the similarity between the cluster heads and the active
molecules. The similarity used here is the Tanimoto coefficient:
/mnt/nfs/home/jklyu/zzz.github/ChemInfTools/utils/cal_Tc_matrix_uint16/cal_Tc
_matrix_uint16 uncluster_head_uint16.fp uncluster_head.smi
uncluster_head_uint16.count knowns_uint16.fp knowns_list.smi
knowns_uint16.count Max_Tc_col

# Format the previous output file by adding a space between the ZINC ID and
TC value:
awk -F "," '{print $1 " " $2}' Max_Tc_col_max_TC.col > Max_Tc_col_space

# Save the cluster head smiles strings:
awk ‘{print $1}’ uncluster_head.smi > uncluster_head_no_id.smi

# Combine cluster heads smiles, ZINC ID and TC value:
paste -d " " uncluster_head_no_id.smi Max_Tc_col_space >
uncluster_head.results
cat uncluster_head.results | awk '{if($3<0.36){print $1 " " $2 " " $3}}' >
uncluster_heads.tc.to.knowns.less.than.0.35.list
awk '{print $2}' uncluster_heads.tc.to.knowns.less.than.0.35.list >
uncluster_heads.less.than.0.35.zinc_id_list

#Once compounds have been clustered and filtered, you can extract poses of
the remaining compounds.
#Within the filtering directory:

python
/mnt/nfs/home/jklyu/zzz.script/large_scale_docking/DOCK/rerank_extract_file.p
y ../../extract_all.top100000.sort.uniq.txt
uncluster_heads.less.than.0.35.zinc_id_list .
```

```
mv extract_all.sort.uniq.re.txt
extract_all.top100000.uncluster.head.filt.0.35.txt
sort -nk 22 extract_all.top100000.uncluster.head.filt.0.35.txt >
extract_all.top100000.uncluster.head.filt.0.35.sort.txt

# Get poses using script below. This is because some files aren't gzipped property and
stops extraction. Output from this is poses-
extract_all.top100000.uncluster.head.filt.0.35.sort.txt.sdf
bash get_poses.sh extract_all.top100000.uncluster.head.filt.0.35.sort.txt

#Copy the necessary files to your Mac to review docked compounds. You will need: the
rec.crg.pdb from  /working and the poses.mol2 from /run1. You can also compare binding
of new docked ligands to the original xtal-lig.pdb from the crystal structure.
```

PAINS/Lip. other filters to remove "bad" compounds

```
## Located in ~/par4/BKS_screen/run1_leadlike/unclustered_posesthatscoredlessthann40/
# Parse compounds in dataset that contain greater than 1 Lipinski violation
and do not pass a PAINS filter test (defined in RDKIT)
# For the record, I haven't looked in depth at the differences between
PAINSA/B/C, but it's in this paper:
https://pubs.acs.org/doi/pdf/10.1021/jm901137j with a follow-up in
https://www.ncbi.nlm.nih.gov/pmc/articles/PMC5778390/pdf/cb7b00903.pdf .
~/anaconda3/envs/my-rdkit-env/bin/python
~/scripts/rdkit_scripts/check_lipinski.py poses-
extract_all.top100000.cluster.head.filt.0.35.sort.0.sdf

#OUPUTS:
# Compounds that failed Lipinski trial: fails_lip.sdf/.png
# X/TOTAL contained 1 Lipinski violation. There were none that had greater
than 1.
# Compounds that failed PAINS(A/B/C) filter: fails_pains(A/B/C).sdf/.png,
reasons listed in fails_pains(A/B/C).txt
# 704 failed PAINS_A
# X failed PAINS_B
# X failed PAINS_C
```

Automate TM depth and pick out a lot more compounds that dig deeper down

```
## Located in ~/par4/BKS_screen/run1_leadlike/unclustered_posesthatscoredlessthann40/
# This uses the mp_transform application in Rosetta
(https://www.rosettacommons.org/docs/latest/application_documentation/membran
e_proteins/RosettaMP-App-MPTransform) to transform the PAR4 receptor with its
docked ligand pose into the membrane coordinate frame using the same span
file that we made previously for the RosettaCM protocol. Once in the membrane
coordinate frame, we simply average (not weighted average) the ligand
coordinates to obtain a centroid and the z-coordinate gives us a measure of
the TM depth.

# Saved poses-extract_all.top100000.cluster.head.filt.0.35.sort.0.sdf into
individual PDBs in ligand_pdbs/ dir.
#ls ligand_pdbs/*.mol > listof_ZINCIDs
#split -l 1700 listof_ZINCIDs
#bash TM_depth.sh xa*
# run using sbatch run_TM_depth.slurm
# run in
~/par4/BKS_screen/run1_leadlike/unclustered_posesthatscoredlessthann40/

ligand_file=$1

for ligand in `awk '{print $1}' ./${ligand_file} `; do
    python2.7 /dors/meilerlab/apps/rosetta/rosetta-
3.10/main/source/scripts/python/public/molfile_to_params.py -n ${ligand} -p
${ligand} --mm-as-virt --long-names --chain
X ./ligand_pdbs/${ligand}_fromdock.sdf
    mv ${ligand}* ligand_pdbs/
    cat ./input_files_for_docking/rec.pdb ./ligand_pdbs/${ligand}_0001.pdb >
./ligand_pdbs/rec_${ligand}.pdb
    /dors/meilerlab/apps/rosetta/rosetta-
3.8/main/source/bin/mp_transform.default.linuxgccrelease -
in:file:s ./ligand_pdbs/rec_${ligand}.pdb -mp:setup:spanfiles ./span.txt -
in:file:extra_res_fa ./ligand_pdbs/${ligand}.params
    mv rec_${ligand}_0001.pdb ligand_pdbs/
    egrep 'ZIN X' ligand_pdbs/rec_${ligand}_0001.pdb >
ligand_pdbs/rec_${ligand}_0001_ligandonly.pdb
    python2.7 ~/docking_benchmark/scripts/get_centroid.py
ligand_pdbs/rec_${ligand}_0001_ligandonly.pdb
ligand_pdbs/rec_${ligand}_0001_ligandonly
```

```
# clean-up so all we have left is the centroid coordinate and the starting
molfile (this will be used to prep for docking).
rm ./ligand_pdbs/rec_${ligand}*[0-
9].pdb ./ligand_pdbs/${ligand}*.pdb ./ligand_pdbs/${ligand}*.params ./ligand_
pdbs/rec_${ligand}*ligandonly.pdb
done

# Using the membrane transformed centroid coordinate
(ligand_pdb/rec_${ligand}_0001_ligandonly.center.pdb), I can choose ligands
whose centroids are closer to TM z-axis=0.
#echo "z_coord" > z_coord.csv
#awk '{print $9}' ligand_pdbs/*center.pdb >> z_coord.csv
#python ./histogram.py z_coord.csv z_coord.png
```

Take compounds where they're more buried in comparison to BMS (n=N)

```
for i in `awk '{print $1}' ./listof_ZINCIDs `; do
    limit=12
    depth=`awk '{print $9}' ./ligand_pdbs/rec_${i}_0001_ligandonly.center.pdb
| awk -F '.' '{print $1}'`
    if [ "$depth" -le "$limit" ]; then
            echo $i >> listof_ZINCIDs_buried
    fi
done

# number below 14: 7006 (7.0%)
# number below 13: 6236 (6.3%)
# number below 12: 5773 (5.8%)
```

Distribution of ligand centroid z-coordinates. Red line designates depth that we believe BMS binds based on docking simulations. Membrane center at (0,0,0).

Strain Analysis and clustering

~/par4/BKS_screen/run1_leadlike/unclustered_posesthatscoredlessthann40/below14_TMdepth/deepTM_strainoutputs_BJB/

4638/7006 (66.2%) passed the strain analysis with TEU threshold=1.8 (pass_strain.id) These resulting compounds then underwent Tanimoto clustering to extract out cluster heads Tc=0.5 (cluster_head.zincid, n=2101) and Tc=0.35 (cluster_head_0.35.zincid, n=840).

Visual analysis

From Brian Bender/Shoichet: "Things to look for in visual analysis: unsatisfied H-bond donors are bad (hydroxlys worse than structural NHs); unsatisfied Hbond acceptors more than 3 or 4; high number of rotatable bonds; axial instead of equatorial moieties on 6-carbon acyl rings; suspicious missed protonation on nitrogens; etc. Once you do a visual culling of hits (using ViewDock in Chimera) send me a chimera session with up to 500 hits and I can help pick from there."

Interface Analyzer

~/par4/BKS_screen/run1_leadlike/unclustered_posesthatscoredlessthann40/below14_TMdepth/deepTM_strainoutputs_BJB/interfaceanalyzer/

I have looked at all the compounds for the above criteria and made a list accordingly. I also have run Rosetta's InterfaceAnalyzer mover to look specifically for unsatisfied hydrogen bonds and the number of hydrogen bonds at the interface. I did allow repacking and final minimization using the PackRotamersMover and FinalMiniimzer.

run_interfaceanalyzer.sh

```
#!/bin/bash

ZINCID=$1
here=`pwd`

# run BCL cleaner/protonate
bcl.exe molecule:Filter -add_h -neutralize -defined_atom_types -3d -
input_filenames ${here}/../pass_strain_files/${ZINCID}.sdf -output_matched
${ZINCID}_clean.sdf -output_unmatched ${ZINCID}_unclean.sdf -message_level
Debug
# run molfiletoparams
python2.7 /dors/meilerlab/apps/rosetta/rosetta-
3.10/main/source/scripts/python/public/molfile_to_params.py -n ${ZINCID} -p
${ZINCID} --mm-as-virt --long-names ${ZINCID}_clean.sdf --chain X


/dors/meilerlab/apps/rosetta/rosetta-
3.10/main/source/bin/rosetta_scripts.linuxgccrelease \
    @ ${here}/interfaceanalyzer.flags \
    -parser:protocol ${here}/interfaceanalyzer.xml \
    -s "${here}/rec.pdb ${here}/${ZINCID}_0001.pdb" \
    -in:file:native "${here}/rec.pdb ${here}/${ZINCID}_0001.pdb" \
    -extra_res_fa ${here}/${ZINCID}.params \
    -out:path:all ${here}/output_files/ \
    -out:file:scorefile rec_${ZINCID}_ia.sc \

rm ${ZINCID}*
```

Interface_analyzer.xml

```
<ROSETTASCRIPTS>
```

```
    <SCOREFXNS>
        <ScoreFunction name="ligand_soft_rep" weights="ligand_soft_rep">
            <Reweight scoretype="fa_elec" weight="0.42"/>
            <Reweight scoretype="hbond_bb_sc" weight="1.3"/>
            <Reweight scoretype="hbond_sc" weight="1.3"/>
            <Reweight scoretype="rama" weight="0.2"/>
        </ScoreFunction>

        <ScoreFunction name="hard_rep" weights="ligandprime">
            <Reweight scoretype="fa_intra_rep" weight="0.004"/>
            <Reweight scoretype="fa_elec" weight="0.42"/>
            <Reweight scoretype="hbond_bb_sc" weight="1.3"/>
            <Reweight scoretype="hbond_sc" weight="1.3"/>
            <Reweight scoretype="rama" weight="0.2"/>
        </ScoreFunction>
    </SCOREFXNS>
    <TASKOPERATIONS>
        <DetectProteinLigandInterface name="repack_interface" cut1="6.0" cut2="8.0"
cut3="10.0" cut4="12.0" design="0" design_to_cys="0"/>
    </TASKOPERATIONS>
    <LIGAND_AREAS>
        <LigandArea name="docking_sidechain" chain="X" cutoff="6.0"
add_nbr_radius="true" all_atom_mode="true" minimize_ligand="10"/>
        <LigandArea name="final_sidechain" chain="X" cutoff="6.0"
add_nbr_radius="true" all_atom_mode="true"/>
        <LigandArea name="final_backbone" chain="X" cutoff="7.0"
add_nbr_radius="false" all_atom_mode="true" Calpha_restraints="0.3"/>
    </LIGAND_AREAS>

    <INTERFACE_BUILDERS>
        <InterfaceBuilder name="side_chain_for_docking"
ligand_areas="docking_sidechain"/>
        <InterfaceBuilder name="side_chain_for_final"
ligand_areas="final_sidechain"/>
        <InterfaceBuilder name="backbone" ligand_areas="final_backbone"
extension_window="3"/>
    </INTERFACE_BUILDERS>

    <MOVEMAP_BUILDERS>
        <MoveMapBuilder name="docking" sc_interface="side_chain_for_docking"
minimize_water="true"/>
        <MoveMapBuilder name="final" sc_interface="side_chain_for_final"
bb_interface="backbone" minimize_water="true"/>
    </MOVEMAP_BUILDERS>
```

```
    <SCORINGGRIDS ligand_chain="X" width="30.0">
        <ClassicGrid grid_name="vdw" weight="1.0"/>
    </SCORINGGRIDS>

    <MOVERS>
    <PackRotamersMover              name="pack_rots"              scorefxn="hard_rep"
task_operations="repack_interface"/>
        <FinalMinimizer name="final" scorefxn="hard_rep" movemap_builder="final"/>
        <InterfaceScoreCalculator  name="add_scores"  chains="X"  scorefxn="hard_rep"
compute_grid_scores="0"/>
    <InterfaceAnalyzerMover      name="interface_analyzer"       scorefxn="hard_rep"
pack_separated="True" pack_input="False" packstat="True" ligandchain="X" />

        <ParsedProtocol name="high_res_dock">
        <Add mover_name="pack_rots"/>
            <Add mover_name="final"/>
        </ParsedProtocol>

        <ParsedProtocol name="reporting">
            <Add mover_name="add_scores"/>
        <Add mover_name="interface_analyzer"/>
        </ParsedProtocol>
    </MOVERS>

    <PROTOCOLS>
        <Add mover_name="high_res_dock"/>
        <Add mover_name="reporting"/>
    </PROTOCOLS>


</ROSETTASCRIPTS>
```

1.3.5 RosettaLigand docking

prep_ligand.sh

```
#!/bin/bash
here=`pwd`
cd $here/ligand_files/

bash ~/scripts/small_molecule/corina_gen3d.sh BMS_study.sdf BMS_study.corina.sdf
bcl.exe molecule:Filter -add_h -neutralize -defined_atom_types -input_filenames
BMS_study.corina.sdf -output_matched BMS_study.corina.cleaned.sdf -output_unmatched
BMS_study.corina.uncleaned.sdf -message_level Debug
python ~/scripts/small_molecule/split_sdf.py BMS_study.corina.cleaned.sdf
rename .sdf .corina.cleaned.sdf V*sdf

for i in V*corina.cleaned.sdf ; do
	for i in V*corina.cleaned.sdf ; do bcl.exe molecule:ConformerGenerator -
ensemble_filenames ${i} -conformers_separate_files ${i%.sdf}.confs.sdf -
max_iterations 8000 -generate_3D -conformation_comparer
"bcl::chemistry::ConformationComparisonInterface" 0.0 -cluster; done
	/dors/meilerlab/apps/rosetta/rosetta-
3.13/main/source/scripts/python/public/molfile_to_params.py -n
${i%.corina.cleaned.sdf} -p ${i%.corina.cleaned.sdf} --mm-as-virt --conformers-in-
one-file ${i%.sdf}.confs.sdf_0.sdf.gz --chain X
done

cd $here/
```

run_docking.sh

```
here=`pwd`
ROSETTA="/dors/meilerlab/apps/rosetta/rosetta-3.13/"

REC=$1 ## mPAR4_files/model_6_score_0001 BMS_analog01
LIG=$2
mkdir -p $here/output_files/${LIG}/

# Run docking
$ROSETTA/main/source/bin/rosetta_scripts.default.linuxgccrelease \
-parser:protocol $here/dock.xml \
-s "$here/${REC}.pdb $here/ligand_files/${LIG}.pdb" \
-in:file:extra_res_fa $here/ligand_files/${LIG}.params \
-out:path:all $here/output_files/${LIG}/ \
-out:file:scorefile ${LIG}.sc \
-nstruct 200 \
-parser:script_vars startfrom="$here/ligand_files/A8.native.center.pdb" \
-mistakes:restore_pre_talaris_2013_behavior \
-score:analytic_etable_evaluation true \
-out:levels all:error \
-out:pdb_gz

cd $here/output_files/${LIG}/
tar -czf ${LIG}_outputs.tar.gz *.pdb.gz --remove-files
cd $here/
```

run_analysis.sh

```
#!/bin/bash
here=`pwd`

input_file=$1 ## listof_ligands
for LIG in `awk '{print $1}' $here/$input_file `; do ## iterate through each of the ligands sequentially
	#rm $here/*${LIG}*txt
	cd $here/output_files_condensed/${LIG}/ ## go to output directory
	#tar -xzf ${LIG}_outputs.tar.gz ## Untar all the outputs
	#python ~/scripts/small_molecule/contacttable.py ${LIG}.sc -g $here/ligand_files/${LIG}.groupings -p $here/ligand_files/${LIG}.params -r 2.5 -z > ${LIG}.contacttable ## Create contact table file.
	score_col=`bash ~/scripts/bash/get_column_from_scorefile_by_name.sh interface_delta_X ${LIG}.sc` ## figure out which column "interface_delta_X" is in.
	num_clusters=`egrep "=== CLUSTER:" ${LIG}.contacttable | tail -n1 | awk '{print $3}'`
	echo "Cluster Rep Population" > $here/${LIG}_cluster_info.csv
	for i in `seq 1 ${num_clusters}` ; do
		rep=`egrep -A3 "=== CLUSTER: ${i} ===" ${LIG}.contacttable | egrep ${LIG}`
		num_models=`egrep -A3 "=== CLUSTER: ${i} ===" ${LIG}.contacttable | egrep "total models" | awk -F ':' '{print $NF}'`
		echo $i $rep $num_models
		echo $i $rep $num_models >> $here/${LIG}_cluster_info.csv
		echo "RMSD and interface scores wrt ${rep}" > $here/${rep}.rmsds_scores.txt
		python ~/scripts/small_molecule/calculate_ligand_rmsd.py S_*${LIG}*.pdb.gz -n ${rep}.pdb.gz -c X -o $here/${rep}.rmsds.txt
		echo "Pose interface_delta_X" > $here/${rep}.scores.txt
		for pdb in `tail -n+2 $here/${rep}.rmsds.txt | awk '{print $1}' | sed 's/.pdb.gz//g'` ; do
			score=`egrep ${pdb} ${LIG}.sc | awk -v score_col=$score_col '{print $score_col}'`
			echo $pdb $score >> $here/${rep}.scores.txt
		done
		paste $here/${rep}.rmsds.txt $here/${rep}.scores.txt >> $here/${rep}.rmsds_scores.txt
	done ## clusters
	rm *_${LIG}_*.pdb.gz
	cd $here/
```

```
      python ~/scripts/docking_analyses/native_pose_recovery_metrics.py -
scorefiles ${clusters_to_plot} -plot ${LIG}.png -summary ${LIG}.csv -x_term
lig_sc_rms -y_term interface_delta_X
done  ## ligands
```

1.3.6 Ligand similarity searching using BCL

align_to_6G3R.sh

```
#!/bin/bash
here=`pwd`

input_file=$1

bcl.exe molecule:Compare ${input_file} $here/../S_0473_6G3R_0029_ligand.sdf -
output ${input_file%sdf}_aligned.output -add_h -message_level Standard -method
"PsiFlexField
             (
                    output_aligned_mol_a =
${input_file%.sdf}_alignedto6G3Rdockedpose_A,
                    output_aligned_mol_b =
${input_file%.sdf}_alignedto6G3Rdockedpose_B,
                    rigid_mol_b = true
```

Common_substructure_searching.sh

```
#!/bin/bash

export BCL="/dors/meilerlab/apps/Linux2/x86_64/bcl/4.0.0/bcl.exe"
here=`pwd`
path_to_libraries="/hd0/smithst/smallmolecule_libraries"
tc_threshold="0.90" ## start at 0.75 because that's what I did for A8
#comp_type="CommonFragmentsTanimoto-rigid"
#comp_type="CommonFragmentsTanimoto-ECFPFragments"
comp_type="LargestCommonDisconnectedSubstructureTanimoto"
listof_libs=$1
#lib="VanderbiltDiscoveryCollection.clean.sdf.gz"

if [ "$listof_libs" == "debug" ]
	then
	echo "RUNNING ON SHORT DEBUG LIBRARY TAKEN FROM FIRST TIME COMPOUNDS OF
$path_to_libraries/Enamine_GPCR_Library_plated_54080cmds_20200524.clean.sdf.gz !!"
	listof_libraries="$here/listof_libraries_debug"
else
	listof_libraries="$here/listof_libraries"
fi

cd $here/
mkdir -p $comp_type/
cd $comp_type/

LIG="6G3R"
#for LIG in `awk '{print $1}' $here/listof_ligands`; do ## 6G3R 6G3T
	mkdir -p ./${LIG}_matches/
	bash ~/scripts/small_molecule/corina_gen3d.sh $here/${LIG}.sdf
$here/${LIG}.corina.sdf
	$BCL molecule:Filter -add_h -neutralize -defined_atom_types -3d -
input_filenames $here/${LIG}.corina.sdf -output_matched
$here/${LIG}.corina.cleaned.sdf -output_unmatched
$here/${LIG}.corina.uncleaned.sdf -message_level Debug
#	for lib in `awk '{print $1}' $listof_libraries`; do
		library_path="$path_to_libraries/$lib"
		if [ "$lib" == "VanderbiltDiscoveryCollection.clean.sdf.gz" ]
			then prop="VU_NUMBER"
		elif [ "$lib" ==
"Enamine_GPCR_Library_plated_54080cmds_20200524.clean.sdf.gz"  ] || [ "$lib" ==
"debug_library.clean.sdf.gz" ]
			then prop="Catalog_ID"
```

```
            elif [ "$lib" == "LC_all_uniq.clean.sdf.gz" ] || [ "$lib" ==
"LC_GPCR_2D_Similarity_Focused_Library_Allosteric_Subset.clean.sdf.gz" ] ||
[ "$lib" == "LC_GPCR_Receptor_Based_Targeted_Library.clean.sdf.gz"  ]
                    then prop="IDNUMBER"
            fi
            #bcl.exe molecule:Compare ${LIG}.corina.cleaned.sdf ${library_path}
-scheduler PThread 8 -method LargestCommonSubstructureTanimoto -output
${LIG}_${lib}.csv -bcl_table_format
            #bcl.exe molecule:Compare $here/${LIG}.corina.cleaned.sdf
${library_path} -scheduler PThread 8 -method 'CommonFragmentsTanimoto((Rigid),atom
comparison=Identity,bond comparison=Identity)' -output
${LIG}_${lib}.${comp_type}.csv -bcl_table_format
            ${BCL} molecule:Compare $here/${LIG}.corina.cleaned.sdf
${library_path} -method ${comp_type} -output ${LIG}_${lib}.${comp_type}.csv -
bcl_table_format

            blah=`wc -l ${library_path%.clean.sdf.gz}.txt | awk '{print $1}'`
            num_in_library=`expr $blah - 1`
            num_in_output=`tail -n1 ${LIG}_${lib}.${comp_type}.csv | tr '\t'
'\n' | tail -n+2 | sed '/^$/d' | wc -l`

            if [ $num_in_library != $num_in_output ]
            then
                    echo "${LIG}_${lib} HAS A PROBLEM!! library: $num_in_library,
output: $num_in_output"
            else
                    tail -n1 ${LIG}_${lib}.${comp_type}.csv | tr '\t' '\n' | tail
-n+2 | sed '/^$/d' > ${LIG}_${lib}.tmp
                    tail -n+2 ${library_path%.clean.sdf.gz}.txt >
${lib%.clean.sdf.gz}.txt.tmp
                    paste ${lib%.clean.sdf.gz}.txt.tmp ${LIG}_${lib}.tmp | tail -
n+2 | sort -r -nk2 > ${LIG}_${lib}.${comp_type}.combined.csv
                    rm ${lib%.clean.sdf.gz}.txt.tmp  ${LIG}_${lib}.tmp
                    awk -v tc=$tc_threshold '$2 >= tc'
${LIG}_${lib}.${comp_type}.combined.csv >
ligands_from_${lib}.${comp_type}.withgreaterthan${tc_threshold}similarity_to_${LIG
}.csv
                    num_matches=`wc -l
ligands_from_${lib}.${comp_type}.withgreaterthan${tc_threshold}similarity_to_${LIG
}.csv | awk '{print $1}'`
                    echo "Found $num_matches for ${LIG} in ${lib}....."
                    for match in `awk '{print $1}'
```

```
ligands_from_${lib}.${comp_type}.withgreaterthan${tc_threshold}similarity_to_${LIG
}.csv `; do
                          #bcl.exe molecule:Filter -input_filenames
${library_path} -output_matched ${match}.sdf -property_has_string ${prop} ${match}
                          cat ${library_path%.sdf.gz}/${match}.clean.sdf >>
${LIG}_matches/ligands_from_${lib}.${comp_type}.withgreaterthan${tc_threshold}simi
larity_to_${LIG}.sdf
                    done ## match
             fi

#      done #lib
#done #ligand
```

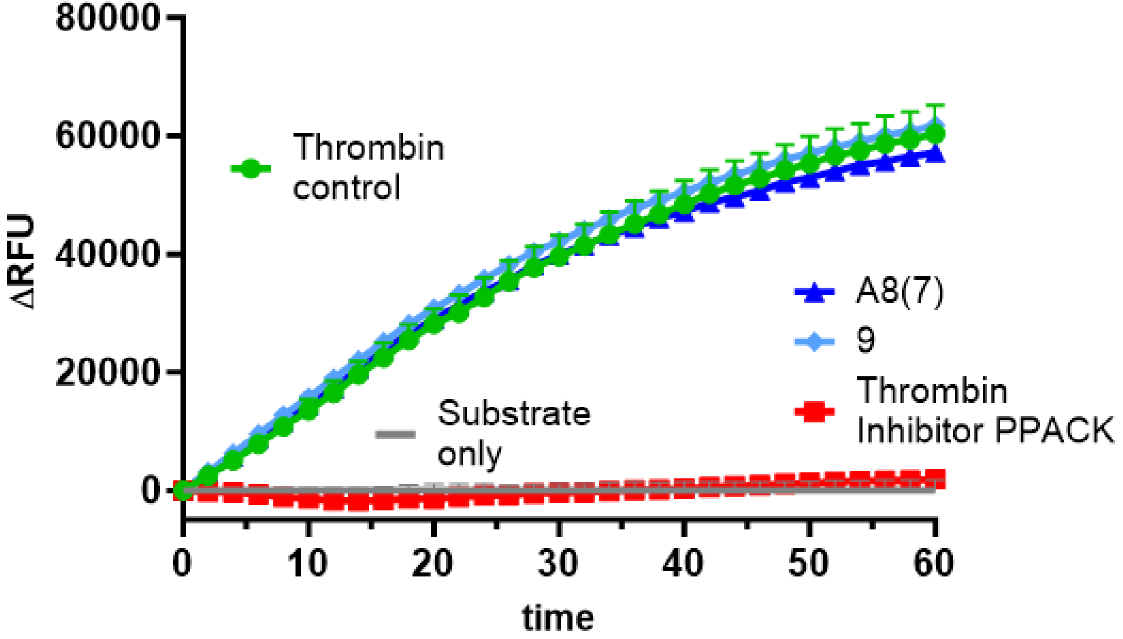

Supplemental Figure S1 *Hit compound 7 and analog* ***9*** *do not inhibition thrombin in an in vitro fluorescence assay of thrombin enzymatic activity.*

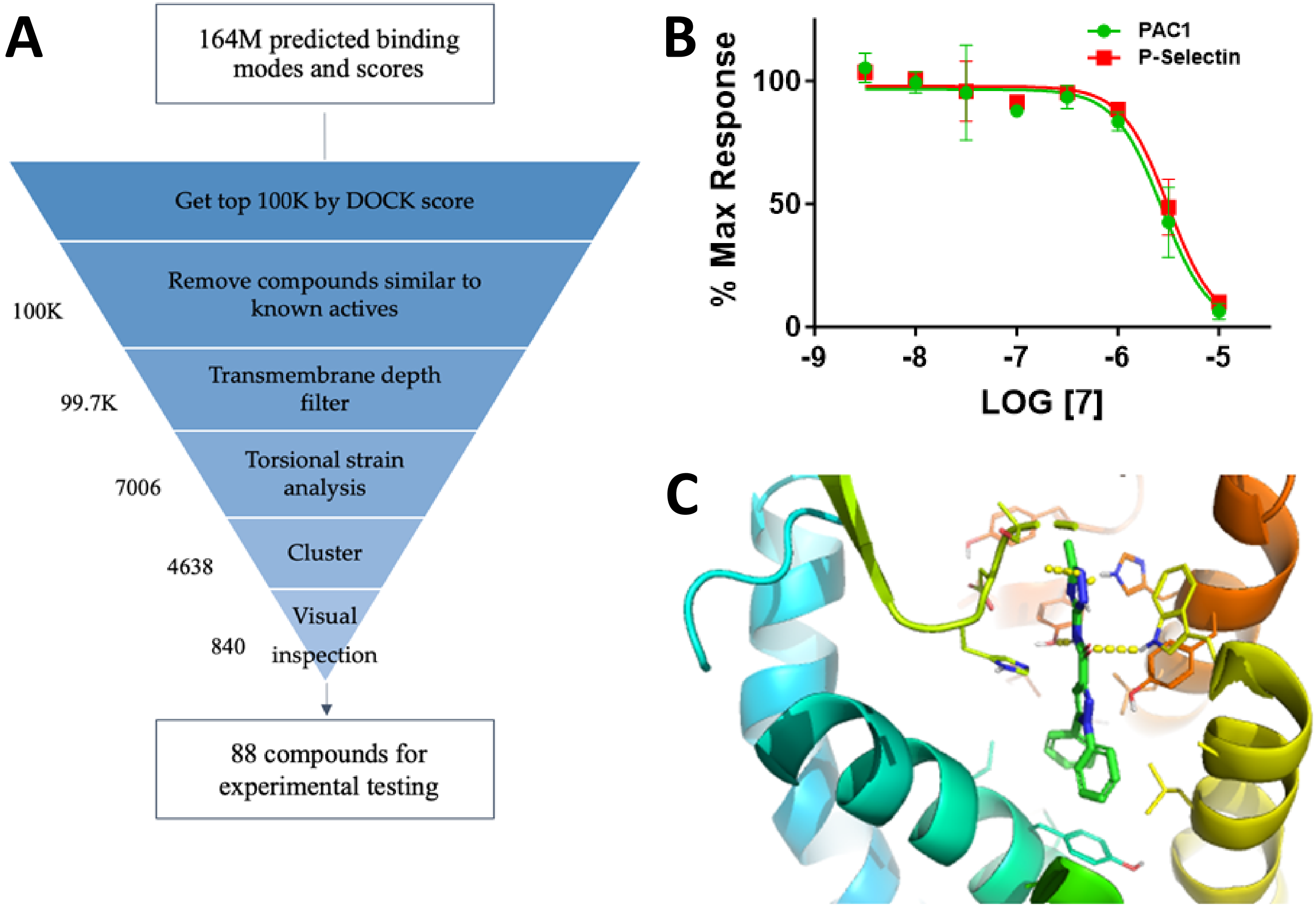

Supplemental Figure S2: *ULS identification of initial hit compound 7 **.** A) Post-screen filtering steps; B) Predicted binding mode of 7 in PAR4 model; C) Dose response curves of purchase 7 and re-synthesized 7.*

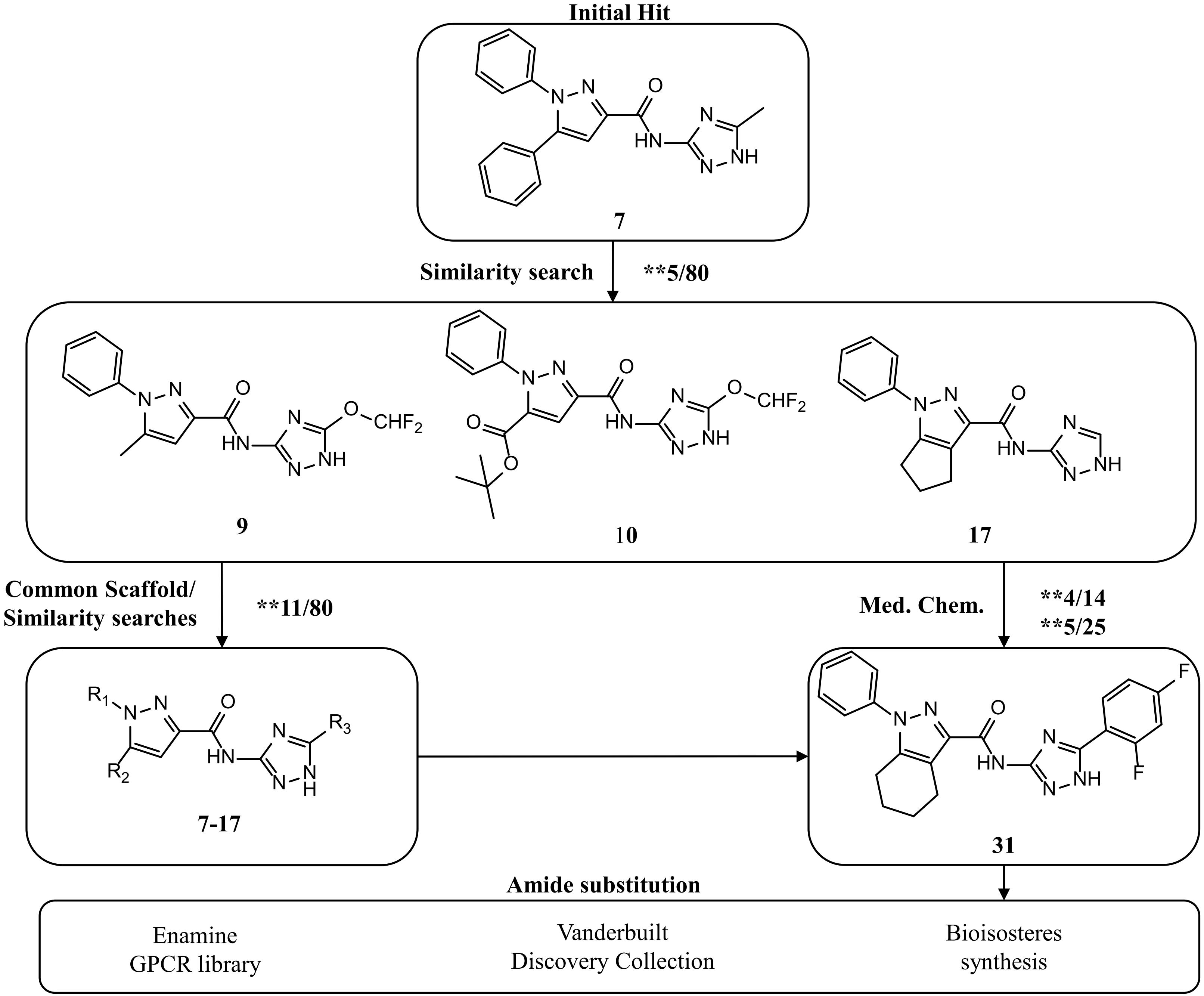

Supplemental Figure S3*: Overview of lead-optimization and hit rates at each stage. * <50% max response tested at 10uM; ** and *** indicate number of compounds <10% max response tested at 10uM and 1 uM, respectively.*

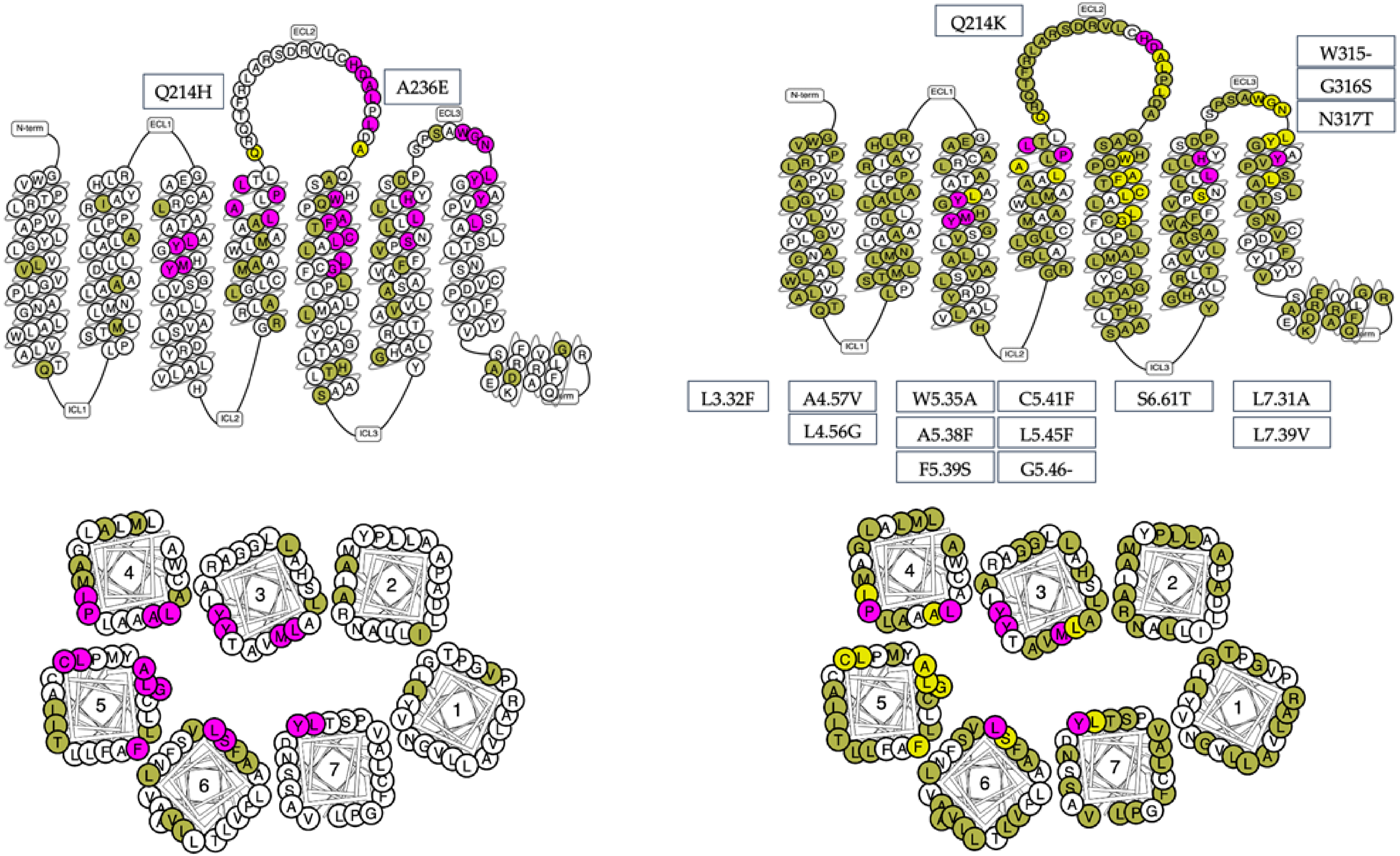

Supplemental Figure S4: *Side and top-down representation of variance between human PAR4 and mouse PAR4 (left) and human PAR1 (right). Residues within 5Å of predicted* ***31*** *binding mode shown in pink, variant residues in brown, and variant residues in the binding site in yellow.*

Compound	Cl_{int} (mL/min/kg)				Estimated % Contribution to Metabolism			
	Human		Mouse		Human		Mouse	
	+NADPH	-NADPH	+NADPH	-NADPH	CYPs/FMOs	Esterases	CYPs/FMOs	Esterases
**17**	26.8	12.4	789	27.8	39	61	100	0
**31**	144	15.6	10438	32.3	80	20	88	12
**12**	642	352	9821	6239	4	96	2	98

Supplemental Table S1: *Modified in vitro microsomal clearance assay in the presence and absence of NADPH and estimated contribution to clearance from CYP450s and FMOs, or Esterases*

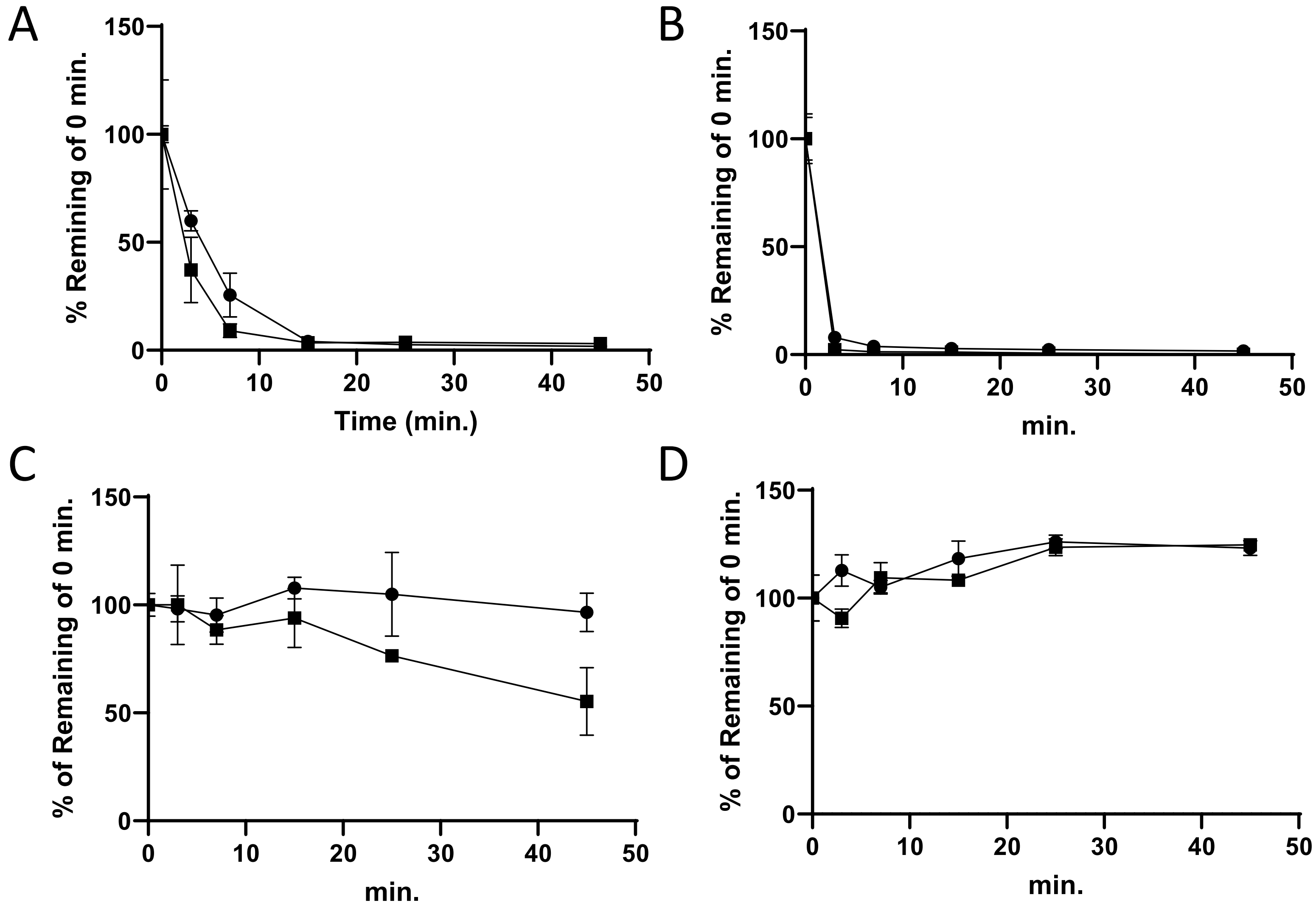

Supplemental Figure S5. *Depletion of* ***12*** *over time in the presence (■) or absence (●) of NADPH. Incubations were carried out in the presence of human (A) or mouse (B) liver microsomes or without microsomes but in either KPI (C) or HP (D) buffer. Values are expressed as percentage of compound remaining at each time compared with time 0 min and represent the mean ± SD (n = 3). Error bars smaller than the symbols are not visible.*

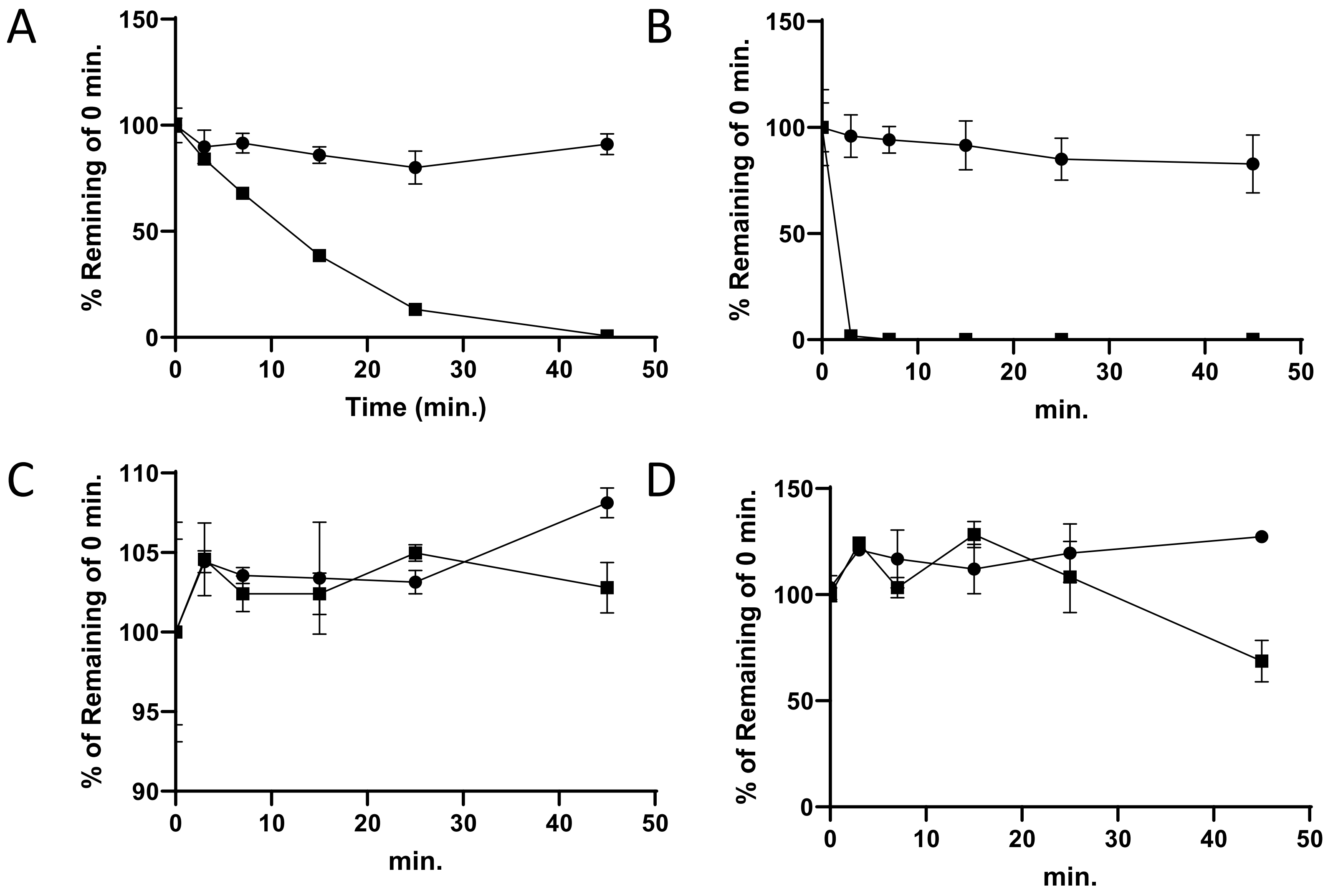

Supplemental Figure S6. *Depletion of* ***31*** *over time in the presence (■) or absence (●) of NADPH. Incubations were carried out in the presence of human (A) or mouse (B) liver microsomes or without microsomes but in either KPI (C) or HP (D) buffer. Values are expressed as percentage of compound remaining at each time compared with time 0 min and represent the mean ± SD (n = 3). Error bars smaller than the symbols are not visible.*

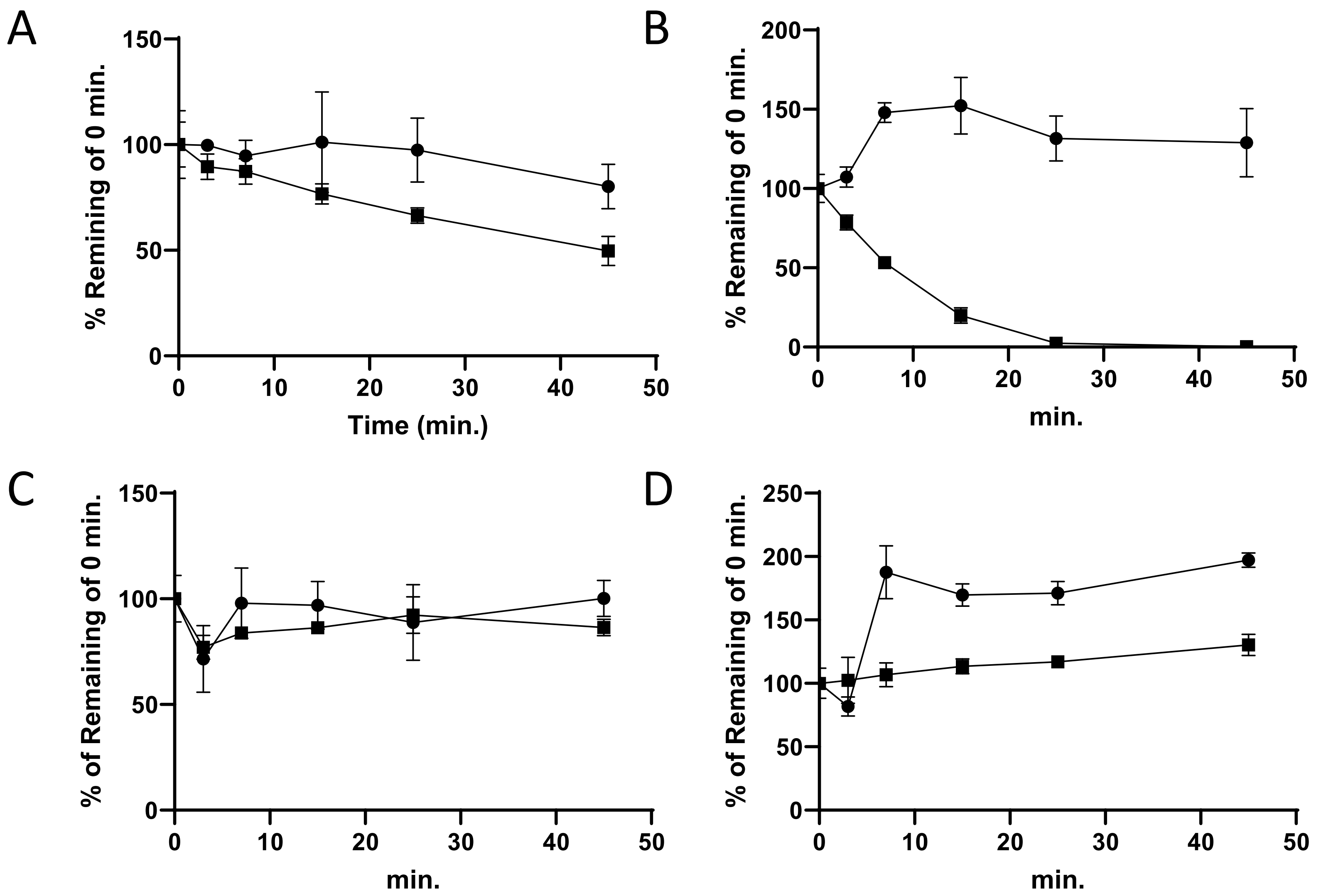

Supplemental Figure S7. *Depletion of* ***17*** *over time in the presence (■) or absence (●) of NADPH. Incubations were carried out in the presence of human (A) or mouse (B) liver microsomes or without microsomes but in either KPI (C) or HP (D) buffer. Values are expressed as percentage of compound remaining at each time compared with time 0 min and represent the mean ± SD (n = 3). Error bars smaller than the symbols are not visible.*
